# Exploration of Regulation of Alcohol Dehydrogenation Reaction of Dibenzimidazole-Based Ruthenium Complexes

**DOI:** 10.3390/molecules30040842

**Published:** 2025-02-12

**Authors:** Shuai Li, Min Jiang, Sander Dekyvere, Peng Wang, Cheng Chen, Francis Verpoort

**Affiliations:** 1 State Key Laboratory of Advanced Technology for Materials Synthesis and Processing, Wuhan University of Technology, Wuhan 430070, China; ls1870881132@163.com (S.L.); 13277236768@163.com (M.J.); sander4a@hotmail.com (S.D.); 331097@whut.edu.cn (P.W.); 2 School of Materials Science and Engineering, Wuhan University of Technology, Wuhan 430070, China; 3 Sanya Science and Education Innovation Park of Wuhan University of Technology, Sanya 572000, China; 4 Joint Institute of Chemical Research (FFMiEN), Peoples Friendship University of Russia (RUDN University), 6 Miklukho-Maklaya Str., 117198 Moscow, Russia; 5 Research School of Chemical and Biomedical Technologies, National Research Tomsk Polytechnic University, Lenin Avenue 30, 634050 Tomsk, Russia

**Keywords:** ruthenium-based complexes, alcohol dehydrogenation, regulatory groups, dibenzimidazole

## Abstract

This work demonstrates the synthesis of a series of Ru(II) complexes with dibenzimidazole-based ligands and investigates the spatial relationship between ligand structure and complexes. It explores the effect of substituent changes on the N-Ru-N bond angle of the complexes, as well as the distance from the Ru center to the aryl group, and utilizes these complexes to catalyze benzyl alcohol dehydrogenation in toluene. It was found that the ligand [Ru]-complex with a spatial configuration of electron donating groups had a larger N-Ru-N bond angle and higher catalytic performance at the ruthenium center, with a yield of 91.2%. Moreover, its distance from the umbrella hydrocarbon was sufficient to allow for the attack of the reactant molecules and the occurrence of catalytic reactions. The reaction mechanism was subsequently derived. This manuscript is expected to provide assistance and inspiration for the development of high-performance catalysts for alcohol dehydrogenation reactions.

## 1. Introduction

The conversion of alcohol compounds into valuable chemical products is of great significance in the high-value-added industry of organic chemistry and the pharmaceutical industry [1,2,3,4]. Due to carboxylic acids being important raw materials and intermediates in the chemical industry, which can be used in the plastic and oil industries, the conversion of alcohols into carboxylic acids is a significant progress that has attracted numerous researchers to explore and study it [5,6,7,8]. Traditional conversion methods typically require the use of toxic or extremely expensive oxidants or reagents and may produce unwanted byproducts such as flammable and explosive mixtures, as well as unstable functional groups that are highly oxidizable [9,10]. Without utilizing the final product, this goes against green and sustainable synthesis strategies.

In recent years, the acceptorless dehydrogenation reactions of alcohols, water, hydroxides, and other substances have become a research focus for obtaining carboxylic acids, as they can achieve substrate dehydrogenation conversion without involving oxidants and additives. Hydrogen gas is the only byproduct, which has the characteristics of green environmental protection and high atomic economic utilization [11,12,13,14]. In 2013, Milstein’s team first reported a catalytic system for PNN Ru complexes, which can achieve the acceptorless dehydrogenation of fatty alcohols to esters without the use of oxidants under alkaline conditions with the help of bipyridine ruthenium complexes, while only releasing a single byproduct, hydrogen gas [15]. Inspired by this work, researchers have explored other metals such as Ag, Pd, Mn, Co, Zn, etc. [16,17,18,19,20,21]. However, due to the high activity of Ru metal in alcohol to carboxylic acid conversion, Ru metal is still one of the research objects in many cases.

In addition, many metal complexes have been developed using ligands such as phosphines, pyridines, amines, and N-heterocyclic carbenes (NHCs) [22,23,24]. Among them, due to the strong σ-donor ligand properties of NHCs, their electrons and spatial distribution can be independently adjusted, and they can replace classic two-electron ligands such as ethers, amines, and phosphines as organic catalysts, which has aroused the research interest of researchers [25,26].

Among these ligands, the unique structures of benzimidazole- or dibenzimidazole-based ligands demonstrate excellent biological activity and electron delocalization ability [27,28]. In addition, dibenzimidazole-based ruthenium metal complexes have rarely been studied for catalytic dehydrogenation reactions. The spatial relationship between the ligand structure and the complex has not been analyzed, and the influence of catalytic performance and structure has not been explored and clarified. Therefore, we synthesized a series of metal ruthenium dibenzimidazole complexes, studied the relationship between their catalytic dehydrogenation performance and their structure, and explored the influence of substituents at different positions on the structure. We found the catalyst with the best catalytic activity, reaching 91.2%, and propose a catalytic mechanism.

## 2. Results and Discussion

### 2.1. Characterization of Ruthenium Complexes

**Figure 1:**
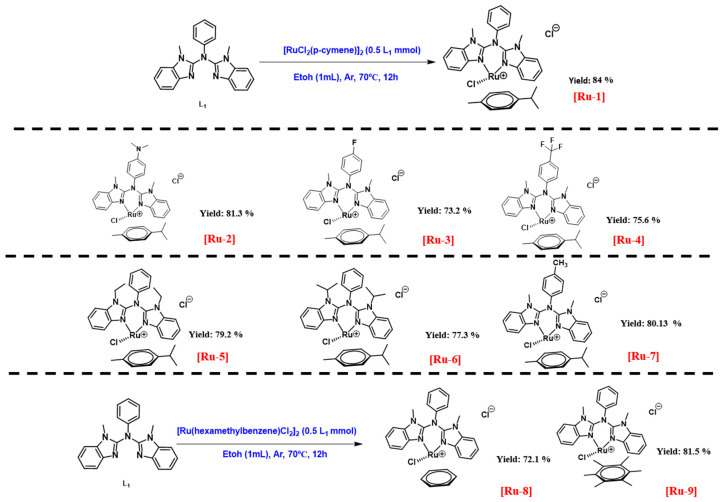
Complex **[Ru-1]**–**[Ru-9]**.

**Figure 2:**
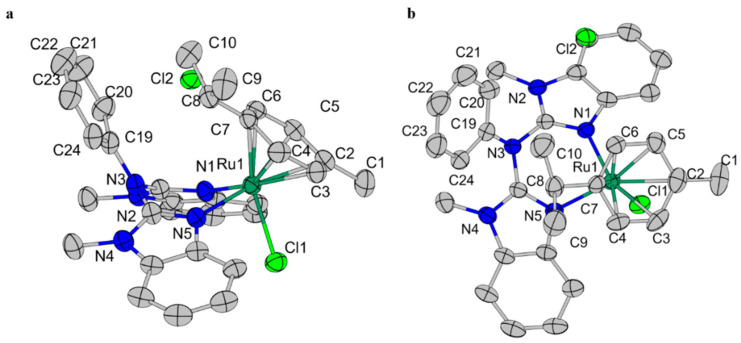
The crystal structure of **[Ru-1]**. (**a**) Vertical coordination plane. (**b**) Aromatic rings stacked vertically downwards. The thermal ellipsoids are drawn with a 50% probability level. For clarity, hydrogen atoms are omitted.

As shown in Figure 1 and Supporting Information, ligand **L_1_** reacted with [Ru(*p*-cymene)Cl_2_]_2_ in refluxed methanol to generate the orange powder complex **[Ru-1]**. The solid-state structure of **[Ru-1]** was confirmed by X-ray single-crystal diffraction studies [29], as shown in Figure 2. The ruthenium ion in the complex coordinated with two N atoms, one Cl atom, and one branched *p*-cymene molecule to form a twisted triangle-tree–piano-chair structure. In order to analyze its structure, we statistically obtained the distance between the center of the partially bonded ethyl ruthenium and the N atom and represented it as r. From a horizontal perspective, r (Co-N^1^) = 2.1124 Å; r (Co-N^5^) = 2.121 Å; r (Ru-Cl^1^) = 2.408 Å; r (Ru Aromatic) = 2.210 Å; and the angle N^1^-Ru-N^5^ = 81.52°. The distance between the ruthenium ion and the aromatic ring of the branched hydrocarbon was replaced by r (Ru Aromatic), and the calculation formula is as follows: r (Ru Aromatic) = (L Ru-C^2^ + L Ru-C^3^ + L Ru-C^4^ + L Ru-C^5^ + L Ru-C^6^ + L Ru-C^7^)/6.

In order to systematically investigate the effects of positional group changes on the overall structure and catalytic performance of ligands, **L_1_** ligand was modified with substituents at C^22^ and C^13^ positions, respectively, to obtain ligands **L_1_**–**L_7_**. These ligands were then coordinated with ruthenium salts ([Ru(*p*-cymene)Cl_2_]_2_, [Ru(phenyl)Cl_2_]_2_, and [Ru(hexamethylbenzene)Cl_2_]_2_) to obtain the stereo-electronic structure of dibenzimidazole-based ligands forming **[Ru-1]**–**[Ru-9]** (Figure 1). As shown in Figure 1, the electron-withdrawing groups -F and -CF_3_ were introduced at the C^22^ position of the clamp-shaped diphenylimidazole ligand in **[Ru-1]**, resulting in **[Ru-3]** and **[Ru-4]**. At the same time, **[Ru-2]** and **[Ru-7]** were obtained, and the electron-donating groups -CH_3_ and-N(CH_3)2_ were used at the same site. To regulate the spatial distribution of dibenzimidazole ligands in **[Ru-1]**, replacing the Me group at the N-terminal of ligand **L_1_** with Et or iPr resulted in **[Ru-5]** or **[Ru-6]**. Additionally, the complexes **[Ru-8]** and **[Ru-9]** were obtained by replacing *p*-cymene with hexamethylbenzene and benzene rings to investigate the effect of ruthenium on ligands, as shown in Figure 2.

**Figure 3:**
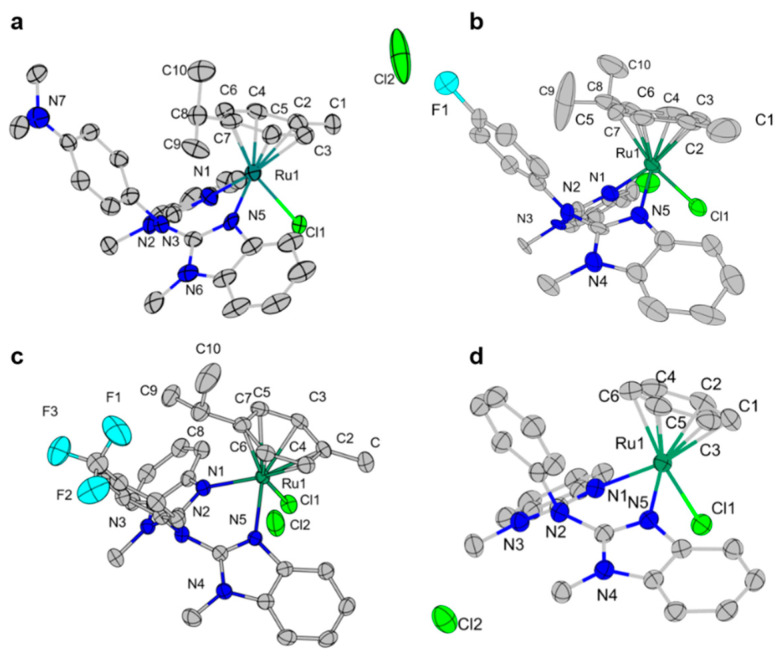
The molecular structures of **[Ru-2]**, **[Ru-3]**, **[Ru-4]**, and **[Ru-9]**. (**a**) **[Ru-2]**, (**b**) **[Ru-3]**, (**c**) **[Ru-4]**, and (**d**) **[Ru-9]**. The thermal ellipsoids are drawn with a 50% probability level. For clarity, hydrogen atoms are omitted.

To our delight, we obtained single crystal structures of **[Ru-2]**, **[Ru-3]**, **[Ru-4]**, and **[Ru-9]** simultaneously and analyzed them (Figure 3); we also present the differences in some structural data in Table 1. We found that the angles of N^1^-Ru-N^5^ in **[Ru-3]** and **[Ru-4]** were 80.85° and 81.30°, respectively, which were smaller than the angle of 81.52° in **[Ru-1]**. The r (Ru-aromatic) values of **[Ru-3]** and **[Ru-4]** were 2.193 Å and 2.196 Å, respectively, which were also lower than **[Ru-1]**’s 2.210 Å. It can be considered that due to the introduction of electron-withdrawing groups at the C^22^ position, the angle and distance between the ruthenium center and the aromatic ring (*p*-cymene, benzene, hexamethylbenzene) decreased. Additionally, due to the stronger electron-withdrawing ability of -F compared to -CF_3_, the angle and r (Ru-aromatic) of **[Ru-3]** were smaller than those of **[Ru-4]**. Meanwhile, the angle of **[Ru-2]** was 82.7°, larger than that of **[Ru-1]**, and its r (Ru-C_aromatic_) was 2.20 Å, which was similar to that of **[Ru-1].** The introduction of N(CH_3_)_2_ increased the angle between the N atom and the ruthenium center, attributed to electron-donating substituents.

After replacing the umbrella hydrocarbon with phenyl, the N^1^-Ru-N^5^ angle of **[Ru-9]** was 82.35° and the distance (r) was 2.181 Å, both of which were smaller than those in **[Ru-1]**. We believe that the benzene ring replacement altered the angle. We consider that the angle between Ru and N reflected the local electron density of ruthenium, while the distance was related to the steric hindrance of ruthenium ions, which affected the catalytic performance of these complexes. For example, a larger angle indicated lower electron density at the Ru center, resulting in a stronger positive charge and enhanced catalytic ability. Conversely, a smaller distance (r) increased steric hindrance, making it more difficult for active molecules to contact the ruthenium center in catalytic reactions, thereby reducing catalytic activity. A comparison of measurement data for five ruthenium complexes is presented in Table 1, with distances expressed in Å and angles in °. The molecular structures of **[Ru-2]**, **[Ru-3]**, **[Ru-4]**, and **[Ru-9]** are shown in Figure 3.

### 2.2. Exploration of Catalytic Performance

To demonstrate the effect of structural modification on the catalytic performance of Ru-series complexes, we studied the catalytic conversion of benzyl alcohol. The optimum reaction conditions were explored using **[Ru-1]** as a model.

In reactions 1, 2, and 3 of Table 2, we screened multiple solvents and selected toluene, meta-xylene, and mesitylene. We found that toluene was the most suitable solvent for this type of catalytic reaction at a reflux temperature. In reactions 1, 4, and 5, we screened KOH, NaOH, and LiOH and found that KOH, with its higher alkalinity, exhibited higher catalytic performance. Additionally, we varied the amount of **[Ru-1]** in reactions 1, 6, 7, 8, and 9 and determined that 0.3% was the optimal amount for catalysis. Increasing the catalyst amount did not yield better results, and the reaction did not proceed without the catalyst. When adjusting the ratio of KOH to **1a**, we found that a ratio of 1:1.5 promoted the catalytic reactions (see entry 10). Furthermore, the reaction did not proceed without the addition of an alkali, indicating that an alkaline condition was essential. We also varied the solvent amount and found that the optimal amount was 0.4 mL (see entry 12).

We found that **[Ru-2]** and **[Ru-7]** had higher conversion rates for benzoic acid compared to **[Ru-1],** see Table 3. We believe that the introduction of electron-donating substituents enhanced the catalytic activity of ruthenium complex centers, thereby improving the yield. Due to the stronger electron donating ability of -N(CH_3_)_2_, **[Ru-2]** exhibited a higher catalytic performance. The yield of benzoic acid in **[Ru-3]** and **[Ru-4]** was 78.6%, which was 83.3% lower than that for **[Ru-1]**. The results indicate that the introduction of electron-withdrawing substituents could reduce the yield of benzoic acid, with **[Ru-3]**, which had stronger electron-withdrawing substituents, showing a lower yield than **[Ru-4]**. Compared to **[Ru-1]**, **[Ru-5]**, and **[Ru-6]**, the carboxylic acid yield of **[Ru-1]** was lower, indicating that replacing ethyl and isopropyl groups at the N^2^ and N^4^ positions reduced its catalytic performance.

Meanwhile, **[Ru-8]** and **[Ru-9]** also exhibited lower catalytic performance, indicating that the substitution of benzene rings and hexamethylbenzene instead of *p*-cymene could reduce catalytic performance. Based on the analysis of single crystal structure data, it was found that the electron-withdrawing substituent at C^22^ position affected the angle of N^1^-Ru-N^5^, thereby impacting the catalytic yield of benzyl alcohol dehydrogenation.

### 2.3. Substrate Range for **[Ru-2]** Catalyzed Catalytic Dehydrogenation

To explore the catalytic range of the ruthenium catalysts, we conducted performance tests on various substrates, see Table 4. By modifying the fourth position of the benzene ring in the benzyl alcohol series with substituents such as a, b, c, d, e, f, and g, we observed that the reactivity of **1d** and **1e** was significantly lower, while the reactivity of **1b**, **1c**, and **1f** was comparable to **1a**. We believe that electron-withdrawing substituents in the substrate decreased the reactivity, whereas substrates with electron-donating substituents had little effect on their reactivity.

We speculate that the large steric hindrance of the substituents affected the interaction between the substrate molecules and the catalyst. This was evident when comparing the reactivity of **1b**, **1g**, and **1h**, where substrates with substituents in the para-position exhibited higher reactivity than those in the meta-position and neighboring positions, respectively. For substrates **1i** and **1j**, which contained naphthalene and thiophene rings, we found their reactivity to be around 70%, similar to the reactivity of the aliphatic ring **1k**, which was also 70.9%. Furthermore, the aliphatic alcohol **1l** exhibited a reactivity similar to **1a**, while **1m** showed relatively lower conversion due to the increased chain length. Additionally, 2-furanmethanol, 3-pyridinmethanol, and 2-pyridinemethanol were investigated, resulting in average to good yields of 48%, 50%, and 86%, respectively, demonstrating the broad substrate scope of the catalyst.

In summary, our Ru series catalysts demonstrated a strong adaptation range across various substrates.

### 2.4. Catalytic Mechanism

**Figure 4:**
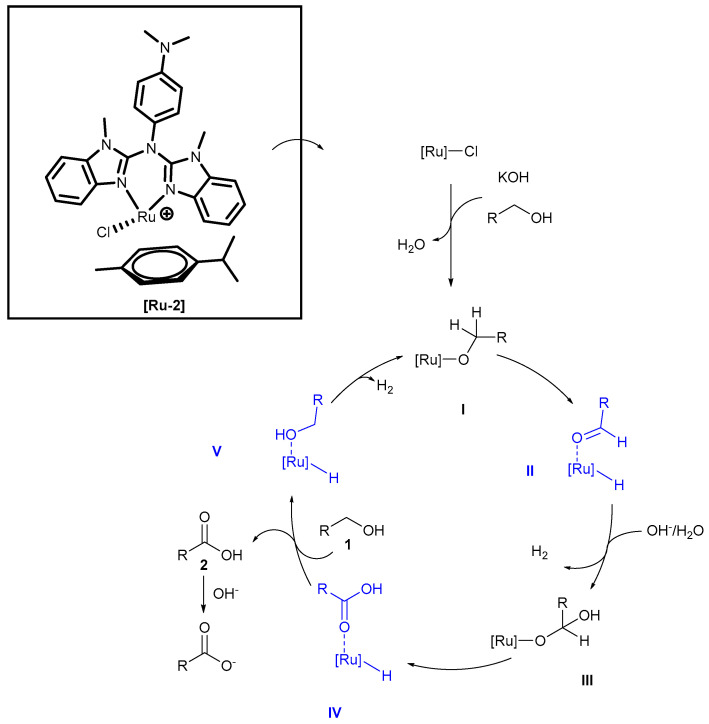
Plausible mechanism for this **[Ru-2]**-catalyzed acid synthesis from alcohols and KOH.

Based on the complex’s structure and the relevant literature [30,31,32], we inferred the potential catalytic mechanism illustrated in Figure 4. Initially, the primary alcohol and KOH reacted with **[Ru-2]** to form the intermediate **I** with the elimination of a water molecule. Through a β-hydride elimination, the intermediate **I** could be converted into intermediate **II**, a Ru hydride bound with the corresponding aldehyde. This was then attacked by H_2_O or OH^−^ to generate dem-diolate, **III**, and one molecule of H_2_. Subsequently, the intermediate **III** underwent another β-hydride elimination, thereby generating the carboxyl-containing species, the intermediate **IV**. The interaction of **IV** with a new primary alcohol led to the release of the carboxylic acid, forming the intermediate **V**. Eventually, the intermediate **V** liberated another molecule of H_2_ and completed the whole catalytic cycle.

## 3. Experiment

### 3.1. Generality

All experiments were conducted in an argon atmosphere using standard Schlenk techniques or in an argon-filled glove box. The NMR spectra were recorded on a Bruker Avance 500 spectrometer (Bruker, Karlsruhe, Germany), with ^1^H NMR at 500 MHz, ^13^C NMR at 126 MHz, and ^19^F NMR at 471 MHz. For NMR analysis, CDCl_3_, DMSO-d_6_, and D_2_O were used as deuterated solvents. The following abbreviations are used to represent multiplicity: s = singlet state; d = doublet state; t = triplet state; q = quadruplet state; p = quintuple state; m = multiplet state; and dd = dual doublet state. All solvents (DMSO, THF, meta-xylene, petroleum ether, ethyl acetate, dichloromethane, toluene-d_8_, CDCl_3_, DMSO-d_6_, D_2_O, etc.), reagents (alcohols 1a-1o KOH, Cs_2_CO_3_, Na_2_SO_4_, [Ru(*p*-cymene)Cl_2_]_2_) and chemicals used for synthesizing the NHC precursors **L_1_**–**L_7_**, as well as column chromatography consumables (silica gel, neutral alumina, silica gel plates, etc.), were purchased from commercial suppliers and were used directly without further processing or purification.

### 3.2. Synthesis

#### 3.2.1. General Synthesis Procedure for Dibenzimidazole Ligands

**Scheme 1:**
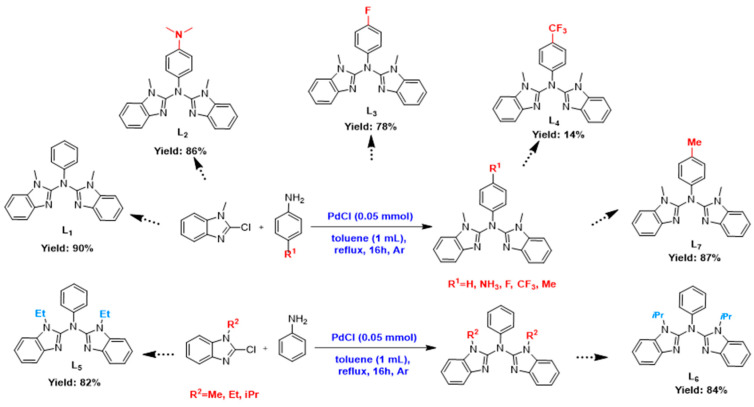
Synthesis of dibenzimidazole ligands **L_1_**–**L_7_**.

Based on our previous work and the literature (Scheme 1), we synthesized dibenzimidazolium ligands (**L_1_**–**L_7_**) as in Scheme 1 [33,34].

##### Synthesis of Ligand (**L_1_**)

Under an argon atmosphere, PdCl_2_ (9.0 mg, 0.05 mmol), 2-chloro-1-methylbenzimidazole (333 mg, 2.0 mmol), Cs_2_CO_3_ (1.034 g, 3.2 mmol), aniline (93.3 μL, 1 mmol), and toluene (5.0 mL) were added to a 25 mL Schlenk flask together with a magnetic stir bar. Then, the mixture was stirred at 120 °C for 16 h. The obtained product was purified by washing it thoroughly with petroleum ether, and the ligand product was a yellow solid with a yield of 90%.

##### Synthesis of Ligands (**L_2_**, **L_3_**, **L_4_**, **L_7_**)

When the aniline was replaced by N, N-dimethyl-1,4-phenylenediamine, 4-fluoroaniline, and 4-trifluoromethylaniline, *p*-toluidine were obtained, according to the synthesis steps of **L_1_**, **L_2_**, **L_3_**, **L_4_**, and **L_7_**.

##### Synthesis of Ligands (**L_5_**, **L_6_**)

When 2-chloro-1-methylbenzimidazole was replaced by 2-chloro-1-ethyl-1H-benzimidazole and 2-chloro-1-isopropyl-1H-benzimidazole, **L_5_** and **L_6_** were obtained according to the synthesis steps of **L_1_**.

#### 3.2.2. General Procedure for Synthesizing **[Ru-1]**, **[Ru-2]**, **[Ru-3]**, **[Ru-4]**, **[Ru-5]**, **[Ru-6]**, and **[Ru-7]**

Based on our previous work and the literature [35], [Ru(*p*-cymene)Cl_2_]_2_ (61.2 mg, 0.1 mmol), **L_1_** (70.6 mg, 0.2 mmol), a stirring rod, and ethanol (6.0mL) were added to a 25 mL Schlenk flask in an argon-filled glove box. After sealing, the flask was placed in an oil bath and refluxed, and the reaction mixture was stirred at 70 °C for 12 h. After cooling the reaction mixture to ambient temperature, the resulting solution was concentrated using a rotary evaporator. A large amount of anhydrous ether was then added to precipitate a pure white complex, **[Ru-1]**, with a yield of 75%. According to the synthesis steps of **[Ru-1]**, the complexes **[Ru-2]**, **[Ru-3]**, **[Ru-4]**, **[Ru-5]**, **[Ru-6]**, and **[Ru-7]** were obtained when ligand **L_1_** was replaced by **L_2_**, **L_3_**, **L_4_**, **L_5_**, **L_6_**, and **L_7_**.

#### 3.2.3. General Procedure for Synthesis of **[Ru-8]** and **[Ru-9]**

In an argon-filled glove box, benzene ruthenium(II) chloride dimer (50.0 mg, 0.1 mmol), **L_1_** (70.6 mg, 0.2 mmol), a stirring magnet, and ethanol (6.0 mL) were added to a 25 mL Schlenk flask. After sealing, the flask was placed in an oil bath, refluxed, and stirred at 70 °C for 12 h. Upon cooling to ambient temperature, the resulting solution was concentrated using rotary evaporation, followed by the addition of a large amount of anhydrous ether to precipitate a pure white complex, **[Ru-8]**, with a yield of 75%. According to the synthesis steps of **[Ru-8]**, the complex **[Ru-9]** was obtained when [Ru(*p*-cymene)Cl_2_]_2_ was replaced by (hexamethylbenzene) ruthenium (II) dichloride.

The supplementary crystallographic data of **[Ru-1]**, **[Ru-2]**, **[Ru-3]**, **[Ru-4]**, and **[Ru-9]** (with the deposition numbers 2409901, 2409640, 2410506, 2409604, and 2409635, respectively) are accessible from the Cambridge Crystallographic Data Centre (CCDC).

### 3.3. X-Ray Crystallography

Single crystal samples were obtained by slowly evaporating petroleum ether into chloroform solutions of **[Ru-1]** (**[Ru-2]** or **[Ru-3]** or **[Ru-4]** or **[Ru-9]**). The diffraction data of these five metal ruthenium complexes were collected by Bruker APEX-II CCD diffractometer (Bruker, Karlsruhe, Germany), using MoK α (λ = 0.71073 Å) radiation. In addition, numerical absorption correction was applied to solve the structure directly, and the anisotropic thermal parameters of all non-hydrogen atoms were refined on F2.

### 3.4. Catalytic Reaction Conditions

Under an argon atmosphere, a 25 mL Schlenk flask was charged with the Ru complex (one of **[Ru-1]**–**[Ru-9]**, 3 μmol), alcohol (1.5 mmol), KOH (101 mg, 1.8 mmol), a stirring magnet, and toluene (0.25 mL). The flask was equipped with a reflux condenser, and the reaction mixture was stirred at a reflux temperature under an argon atmosphere. After the specified reaction time, the mixture was cooled to room temperature. Subsequently, 6 M HCl (3 mL) and water (3 mL) were added, and the reactants along with any unreacted raw materials were extracted using ethyl acetate. The yield was determined by ^1^H NMR analysis.

## 4. Conclusions

In summary, we reported the synthesis of a series of aromatic Ru (II) complexes with dibenzimidazole-based ligands and investigated the spatial relationship between ligand structure and the complexes. We found that changes in substituents significantly affected the N-Ru-N bond angle of the complexes, as well as the distance from the Ru center to the aryl group. These complexes were used to catalyze the dehydrogenation of benzyl alcohol in toluene. The results indicate that the properties of the coordinating dibenzimidazole ligand had a significant impact on the catalytic performance of the studied complexes. Specifically, the complex **[Ru-2]**, coordinated with ligand **L_2_**, featured a spatial configuration with electron-donating groups, resulting in a larger N-Ru-N bond angle and enhanced catalytic activity at the ruthenium center. Moreover, its distance from the umbrella hydrocarbon was sufficient to allow for the attack of the substrate molecules and the occurrence of catalytic reactions. Subsequently, a possible reaction mechanism was proposed. Therefore, the complex **[Ru-2]**, containing **L_2_**, exhibited the highest catalytic performance, and this type of catalyst has a strong substrate adaptability. We hope that this study provides valuable insights and inspiration for the development of high-performance alcohol dehydrogenation catalysts.

## Figures and Tables

**Table 1:** Comparison of measurement data for five ruthenium complexes.

[Ru]	Angle(N^1^-Ru-N^5^) (°)	r(Ru-C_Aromatic_) (Å)	r(Ru-N^1^) (Å)	r(Ru-N^5^) (Å)
**[Ru-1]**	81.52	2.210	2.124	2.121
**[Ru-2]**	82.7	2.20	2.117	2.082
**[Ru-3]**	80.85	2.193	2.111	2.104
**[Ru-4]**	81.30	2.196	2.123	2.111
**[Ru-9]**	82.36	2.181	2.100	2.103

**Table 2:** Conversion of benzyl alcohol using the **[Ru-1]**-based catalytic system.

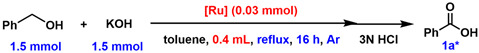						
Entry	Y (mmol)	Solvents	Base	Z (mmol)	X (mL)	1a
1	0.002	toluene	KOH	1.00	1.0	53.0%
2	0.002	m-xylene	KOH	1.00	1.0	39.3%
3	0.002	mesitylene	KOH	1.00	1.0	51.4%
4	0.002	toluene	NaOH	1.00	1.0	33.0%
5	0.002	toluene	LiOH	1.00	1.0	16.6%
6	0	toluene	KOH	1.00	1.0	0.00%
7	0.001	toluene	KOH	1.00	1.0	36.9%
8	0.003	toluene	KOH	1.00	1.0	64.0%
9	0.004	toluene	KOH	1.00	1.0	63.1%
10	0.003	toluene	KOH	1.50	1.0	81.0%
11	0.003	toluene	KOH	0	0.4	0.0%
12	0.003	toluene	KOH	1.50	0.4	85.8%

* To calculate the yield of **1a**, 0.5 mmol of 1,3,5-trimethoxybenzene was used as an internal standard for ^1^H-NMR analysis.

**Table 3:** Conversion of benzyl alcohol of the **[Ru]**-based catalytic system.

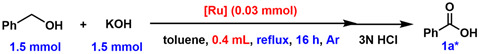			
Entry	[Ru]	Base	1a
1	**[Ru-1]**	KOH	85.8%
2	**[Ru-2]**	KOH	91.2%
3	**[Ru-3]**	KOH	78.6%
4	**[Ru-4]**	KOH	83.3%
5	**[Ru-5]**	KOH	74.2%
6	**[Ru-6]**	KOH	78.3%
7	**[Ru-7]**	KOH	86.1%
8	**[Ru-8]**	KOH	79.2%
9	**[Ru-9]**	KOH	83.6%

* To calculate the yield of **1a**, 0.5 mmol of 1,3,5-trimethoxybenzene was used as an internal standard for ^1^H-NMR analysis.

**Table 4:** Substrate range for **[Ru-2]**-catalyzed catalytic dehydrogenation.

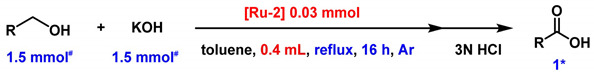
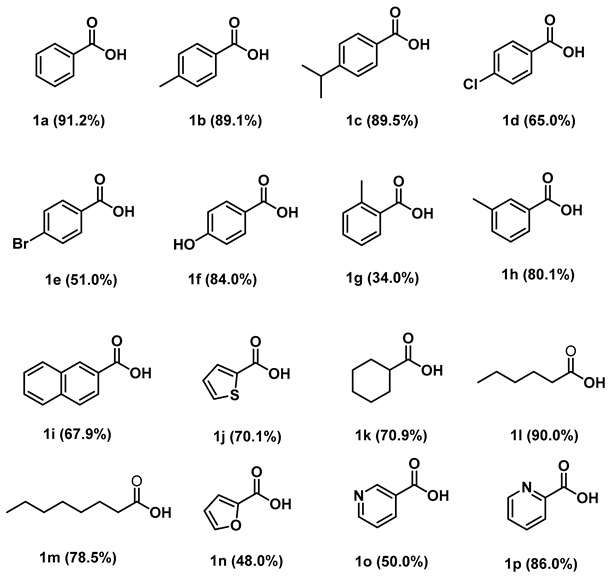

# The reaction substrates were all 1.5 mmol; * to calculate the product yield obtained from the reaction mixture, 0.5 mmol of 1,3,5-trimethoxybenzene was used as an internal standard for ^1^H-NMR analysis. Note that 0.25 mmol of DMF was added as an internal standard for 2-furancarboxylic acid (**1n**) and 3-pyridinic acid (**1o**), while 0.1 mmol DMF was added for 2-pyridinecarboxylic acid (**1p**).

## Data Availability

The data presented in this study are openly available in the Supporting Information of this manuscript.

## Supplementary Materials

The following supporting information can be downloaded at https://www.mdpi.com/article/10.3390/molecules30040842/s1. Figure S1: ^1^H-NMR spectrum of **L_1_**; Figure S2: ^13^C-NMR spectrum of **L_1_**; Figure S3: ^1^H-NMR spectrum of **L_2_**; Figure S4: ^13^C-NMR spectrum of **L_2_**; Figure S5: ^1^H-NMR spectrum of **L_3_**; Figure S6: ^13^C-NMR spectrum of **L_3_**; Figure S7: ^1^H-NMR spectrum of **L_4_**; Figure S8: ^13^C-NMR spectrum of **L_4_**; Figure S9: ^1^H-NMR spectrum of **L_5_**; Figure S10: ^13^C-NMR spectrum of **L_5_**; Figure S11: ^1^H-NMR spectrum of **L_6_**; Figure S12: ^13^C-NMR spectrum of **L_6_**; Figure S13: ^1^H-NMR spectrum of **L_7_**; Figure S14: ^13^C-NMR spectrum of **L_7_**; Figure S15: ^1^H-NMR spectrum of **[Ru-1]**; Figure S16: ^13^C-NMR spectrum of **[Ru-1]**; Figure S17: ^1^H-NMR spectrum of **[Ru-2]**; Figure S18: ^13^C-NMR spectrum of **[Ru-2]**; Figure S19: ^1^H-NMR spectrum of **[Ru-3]**; Figure S20: ^19^F-NMR spectrum of **[Ru-3]**; Figure S21: ^13^C-NMR spectrum of **[Ru-3]**; Figure S22: ^1^H-NMR spectrum of **[Ru-4]**; Figure S23: ^19^F-NMR spectrum of **[Ru-4]**; Figure S24: ^13^C-NMR spectrum of **[Ru-4]**; Figure S25: ^1^H-NMR spectrum of **[Ru-5]**; Figure S26: ^13^C-NMR spectrum of **[Ru-5]**; Figure S27: ^1^H-NMR spectrum of **[Ru-6]**; Figure S28: ^13^C-NMR spectrum of **[Ru-6]**; Figure S29: ^1^H-NMR spectrum of **[Ru-7]**; Figure S30: ^13^C-NMR spectrum of **[Ru-7]**; Figure S31: ^1^H-NMR spectrum of **[Ru-8]**; Figure S32: ^13^C-NMR spectrum of **[Ru-8]**; Figure S33: ^1^H-NMR spectrum of **[Ru-9]**; Figure S34: ^13^C-NMR spectrum of **[Ru-9]**; Figure S35: ^1^H-NMR spectrum of **1a**; Figure S36: ^13^C-NMR spectrum of **1a**; Figure S37: ^1^H-NMR spectrum of **1b**; Figure S38: ^13^C-NMR spectrum of **1b**; Figure S39: ^1^H-NMR spectrum of **1c**; Figure S40: ^13^C-NMR spectrum of **1c**; Figure S41: ^1^H-NMR spectrum of **1d**; Figure S42: ^13^C-NMR spectrum of **1d**; Figure S43: ^1^H-NMR spectrum of **1e**; Figure S44: ^13^C-NMR spectrum of **1e**; Figure S45: ^1^H-NMR spectrum of **1f**; Figure S46: ^13^C-NMR spectrum of **1f**; Figure S47: ^1^H-NMR spectrum of **1g**; Figure S48: ^13^C-NMR spectrum of **1g**; Figure S49: ^1^H-NMR spectrum of **1h**; Figure S50: ^13^C-NMR spectrum of **1h**; Figure S51: ^1^H-NMR spectrum of **1i**; Figure S52: ^13^C-NMR spectrum of **1i**; Figure S53: ^1^H-NMR spectrum of **1j**; Figure S54: ^13^C-NMR spectrum of **1j**; Figure S55: ^1^H-NMR spectrum of **1k**; Figure S56: ^13^C-NMR spectrum of **1k**; Figure S57: ^1^H-NMR spectrum of **1l**; Figure S58: ^13^C-NMR spectrum of **1l**; Figure S59: ^1^H-NMR spectrum of **1m**; Figure S60: ^13^C-NMR spectrum of **1m**; Figure S61: ^1^H-NMR spectrum of **1n**; Figure S62: ^13^C-NMR spectrum of **1n**; Figure S63: ^1^H-NMR spectrum of **1o**; Figure S64: ^13^C-NMR spectrum of **1o**; Figure S65: ^1^H-NMR spectrum of **1p**; Figure S66: ^13^C-NMR spectrum of **1p**; Table S1: Comparison table of different catalysts for alcohol dehydrogenation. Refs. [30,31,36,37,38,39,40] are cited in Supplementary Materials.
