# Supplementary material for: Exploration of Regulation of Alcohol Dehydrogenation Reaction of Dibenzimidazole-Based Ruthenium Complexes
Source: Molecules. 2025 Feb 12;30(4):842. doi: 10.3390/molecules30040842 (PMC11858806; doi:10.3390/molecules30040842)
Supplement: Supplementary file 1 [file molecules-30-00842-s001.zip › molecules-3404063-supplementary.pdf]

Supporting Information

Exploration of the Regulation of Alcohol Dehydrogenation Reaction of Dibenzimidazole-based Ruthenium Complexes

Shuai Li[a b #], Min Jiang[a b #], Sander Dekyvere [a], Peng Wang [a], Cheng Chen[a c *], Francis Verpoort [a d e *]

[a] State Key Laboratory of Advanced Technology for Materials Synthesis and Processing, Wuhan University of Technology, Wuhan 430070, China

[b] School of Materials Science and Engineering, Wuhan University of Technology, Wuhan 430070, China

[c] Sanya Science and Education Innovation Park of Wuhan University of Technology, Sanya, 572000, China

[d] Joint Institute of Chemical Research (FFMiEN), Peoples Friendship University of Russia (RUDN University), 6 Miklukho-Maklaya Str., 117198 Moscow, Russia

[e] National Research Tomsk Polytechnic University, Lenin Avenue 30, 634050 Tomsk, Russia

#: both authors contributed equally

Corresponding Authors: F. Verpoort: francis@whut.edu.cn

Content

1 Synthesis of [Ru] series complexes......1
1.1 Synthesis of [Ru-1] - [Ru-7] 1
1.2 Synthesis of [Ru-8] - [Ru-9] 1
2. Characterization data......2
2.1 Characteristic data of ligands......2
2.2 Characteristic data of [Ru-1] - [Ru-9] (NMR and HR-MS)......12
3 Purified products ^{1}H and ^{13}C NMR spectra 30
3.1 1a......30
3.2 1b......31
3.3 1c......32
3.4 1d......33
3.5 1e......34
3.6 1f 35
3.7 1g......36
3.8 1h......37
3.9 1i 38
3.10 1j 39
3.11 1k......40
3.12 1l 41
3.13 1m 42
3.14 1n......43
3.15 1o......44
3.16......45
1p 45
Table S1.Comparison table of different catalysts catalyzing benzyl alcohol to acid 46

1 Synthesis of [Ru] series complexes

1.1 Synthesis of [Ru-1] - [Ru-7]

Under an argon atmosphere, **L_1** (0.1 mmol), [Ru(p-cymene)Cl_2]$_2$ (0.05 mmol), and anhydrous ethanol (1 mL) were added to a 25 mL Schlenk flask together with a stirring magnet. Then the mixture was stirred at 70℃ for 12 hours. The obtained product was thoroughly washed and purified with petroleum ether, and the **[Ru-1]** product was a yellow solid with a yield of 84%.

Using **L_2, L_3, L_4, L_5, L_6, L_7** as ligands, **[Ru-2] - [Ru-7]** were obtained using the same method.

1.2 Synthesis of [Ru-8] - [Ru-9]

Under an argon atmosphere, **L_1** (0.1 mmol), [Ru(hexamethylbenzene)Cl_2]$_2$ (0.05 mmol), and anhydrous ethanol (1 mL) were added to a 25 mL Schlenk flask together with a stirring magnet. Then the reaction mixture was stirred at 70℃ for 12 hours. The obtained product was thoroughly washed and purified with petroleum ether. The **[Ru-8]** and **[Ru-9]** products were yellow and brown solids with yields of 72.1% and 81.5%, respectively.

2. Characterization data

2.1 Characteristic data of ligands

2.1.1 L_1

^{1}H NMR (500 MHz, DMSO-d_6) δ 7.52 (d, J = 7.9 Hz, 4H), 7.43 (t, J = 7.8 Hz, 2H), 7.32-7.18 (m, 5H), 7.03 (d, J = 7.8 Hz, 2H), 3.42 (s, 6H).

^{13}C NMR (126 MHz, $CDCl_3$) δ 149.3, 143.5, 141.3, 135.1, 129.9, 124.5, 122.7, 122.4, 120.4, 119.7, 109.3, 30.3.

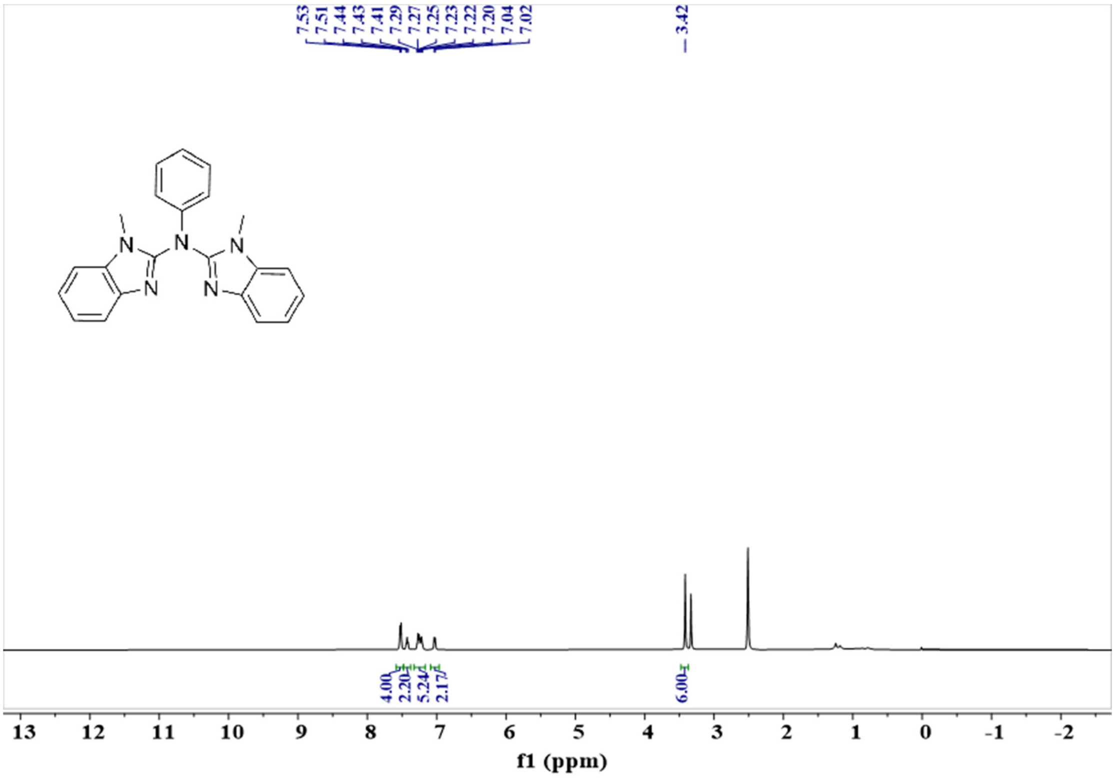

Figure S1 ^{1}H-NMR spectrum of $\mathbf{L_1}$

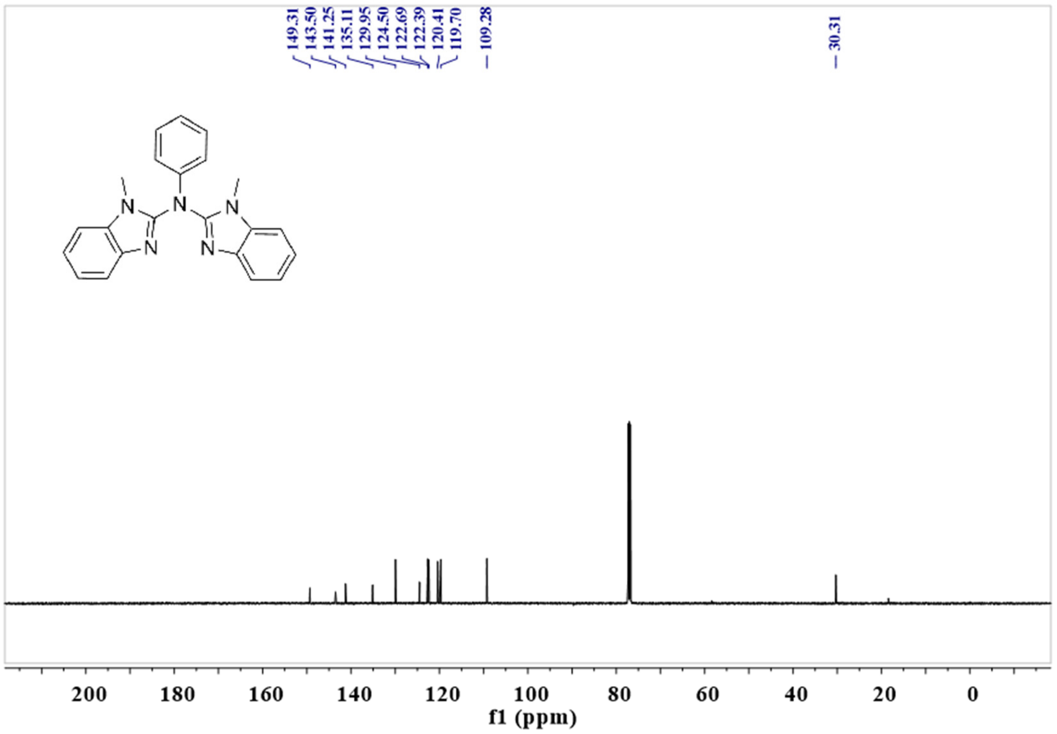

Figure S2 ^{13}C-NMR spectrum of **L₁**

2.1.2 L₂

^{1}H NMR (500 MHz, DMSO-d_6) δ 7.46 (d, *J* = 8.2 Hz, 4H), 7.26-7.14 (m, 4H), 6.99 (d, *J* = 8.8 Hz, 2H), 6.77 (d, *J* = 8.8 Hz, 2H), 3.37 (s, 6H), 2.93 (s, 6H).

^{13}C NMR (126 MHz, $CDCl_3$) δ 150.8, 148.7, 141.4, 135.3, 132.5, 124.3, 122.1, 122.0, 119.4, 113.6, 108.8, 40.7, 30.2.

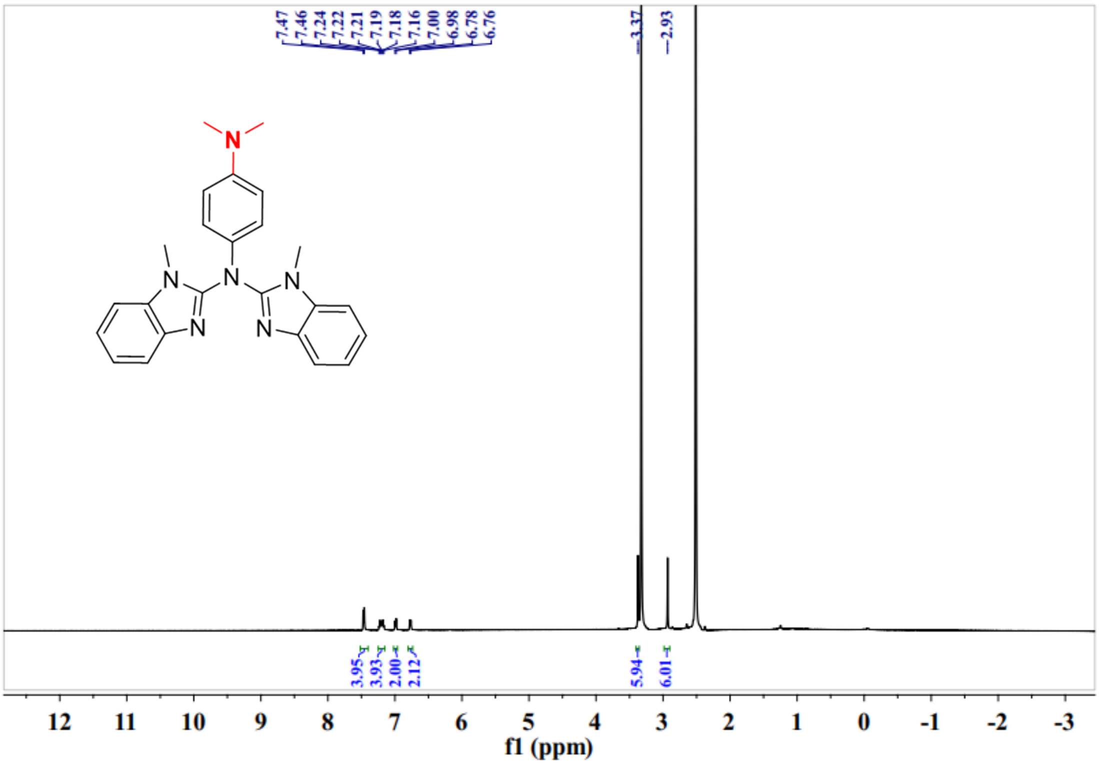

Figure S3 ^{1}H-NMR spectrum of **L$_2$**

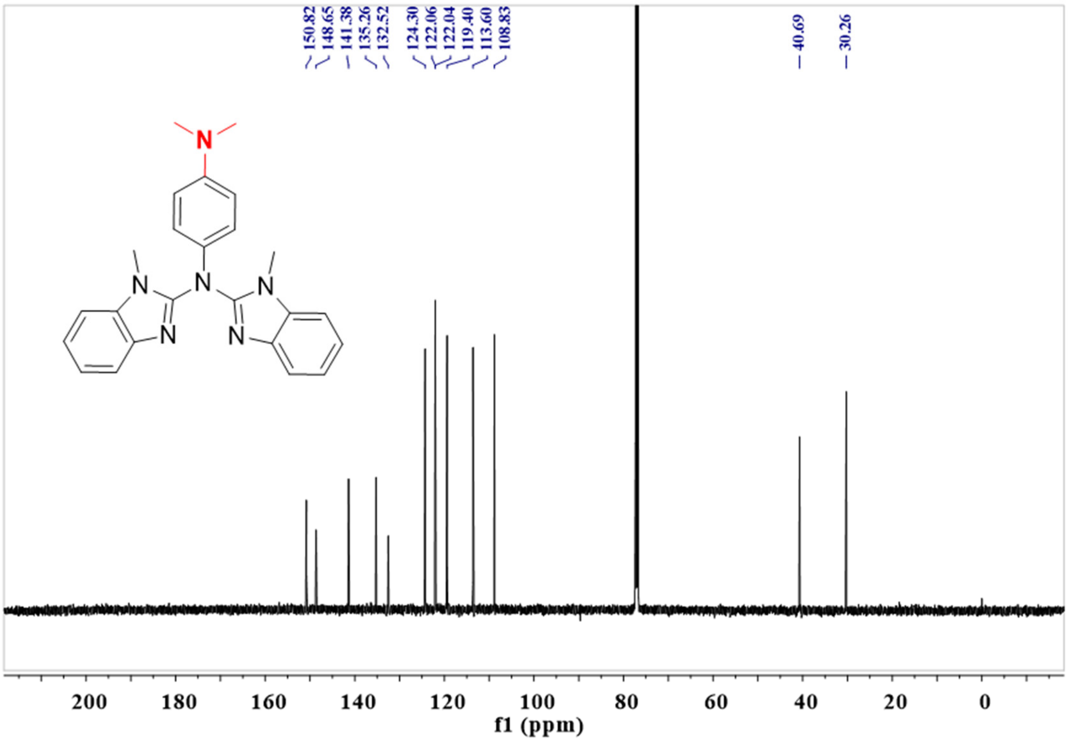

Figure S4 ^{13}C-NMR spectrum of **L$_2$**

2.1.3 L$_3$

^{1}H NMR (500 MHz, DMSO-d$_6$) δ 7.52 (t, *J* = 7.3 Hz, 4H), 7.31 – 7.24 (m, 4H), 7.21 (t, *J* = 7.6 Hz, 2H), 7.14 (dd, *J* = 8.9, 4.7 Hz, 2H), 3.41 (s, 6H).

^{13}C NMR (126 MHz, CDCl$_3$) δ 161.0, 158.9, 149.6, 141.1, 139.4 (d, *J* = 2.9 Hz), 135.0, 123.3 (d, *J* = 8.3 Hz), 122.5 (d, *J* = 29.9 Hz), 119.6, 116.8 (d, *J* = 22.9 Hz), 109.1, 30.2.

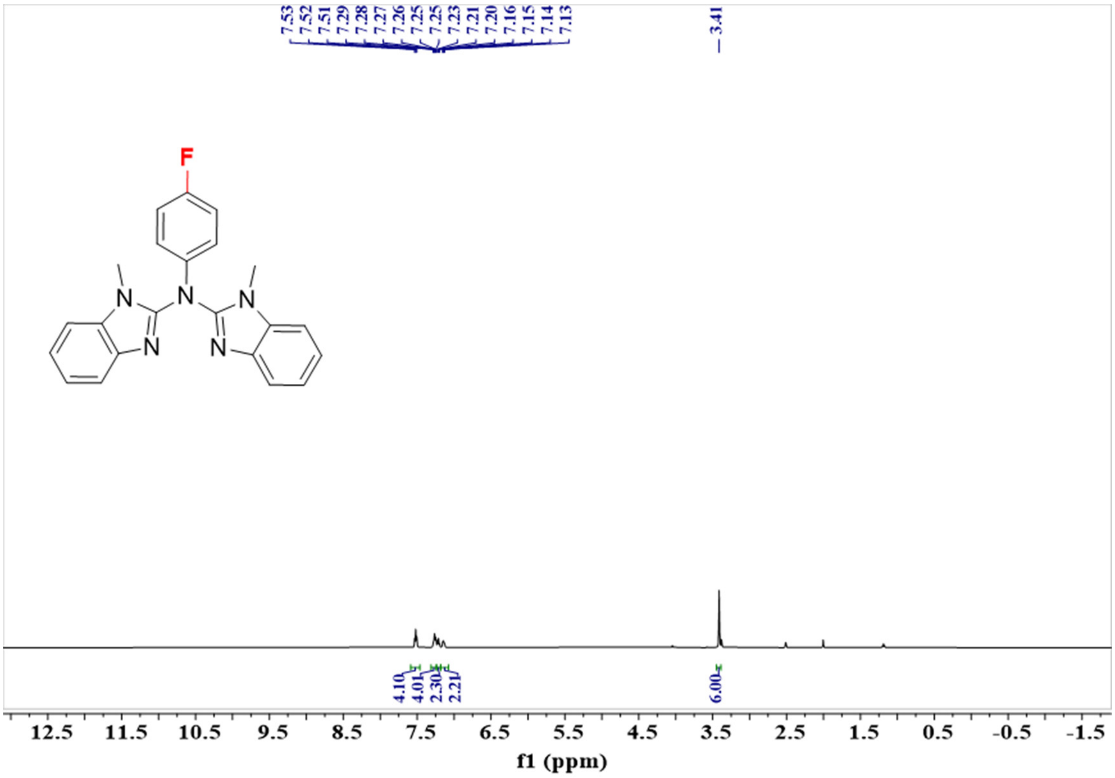

Figure S5 ^{1}H-NMR spectrum of **L$_3$**

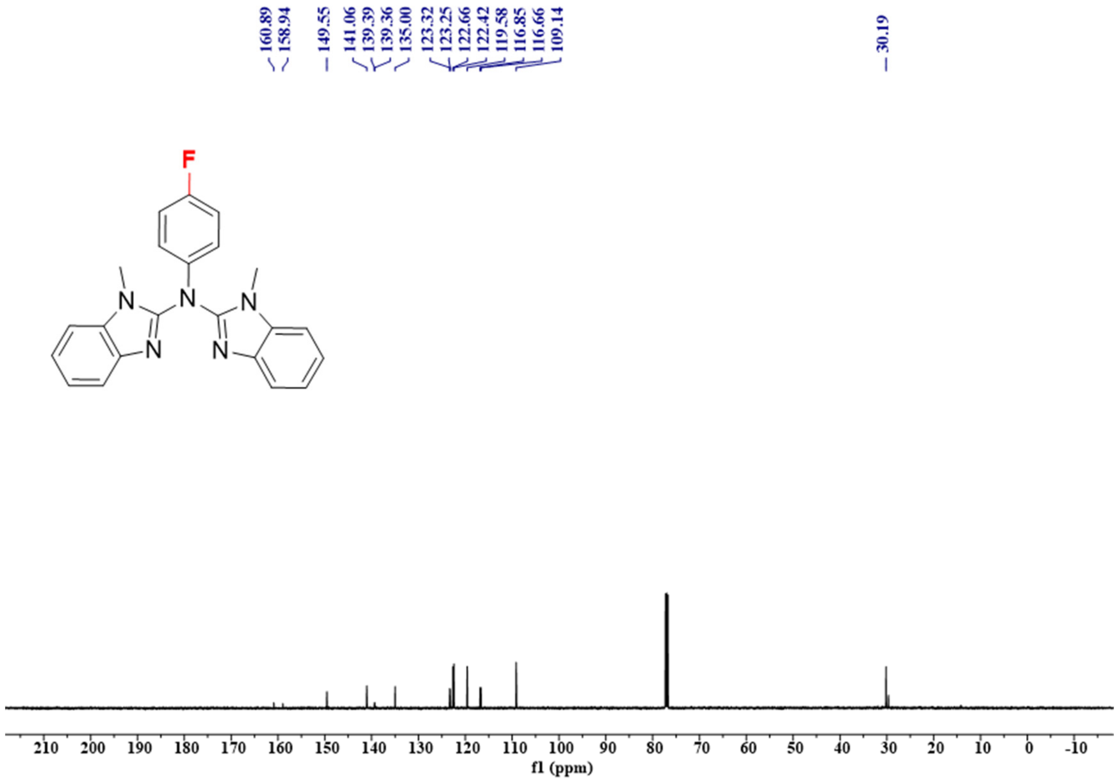

Figure S6 ^{13}C-NMR spectrum of **L$_3$**

2.1.4 L$_4$

^{1}H NMR (500 MHz, DMSO-d$_6$) δ 7.76 (d, *J* = 8.3 Hz, 2H), 7.57 (dd, *J* = 8.0, 3.2 Hz, 4H), 7.31 (t, *J* = 7.7 Hz, 2H), 7.25 (t, *J* = 7.6 Hz, 2H), 7.14 (d, *J* = 8.3 Hz, 2H), 3.49 (s, 6H).

^{13}C NMR (126 MHz, CDCl$_3$) δ 147.9, 146.7, 141.1, 135.0, 127.2 (q, *J* = 3.9 Hz), 125.8 (q, *J* = 32.8 Hz), 124.0 (q, *J* = 288.0 Hz), 123.3, 122.8, 120.0, 118.7, 109.6, 30.3.

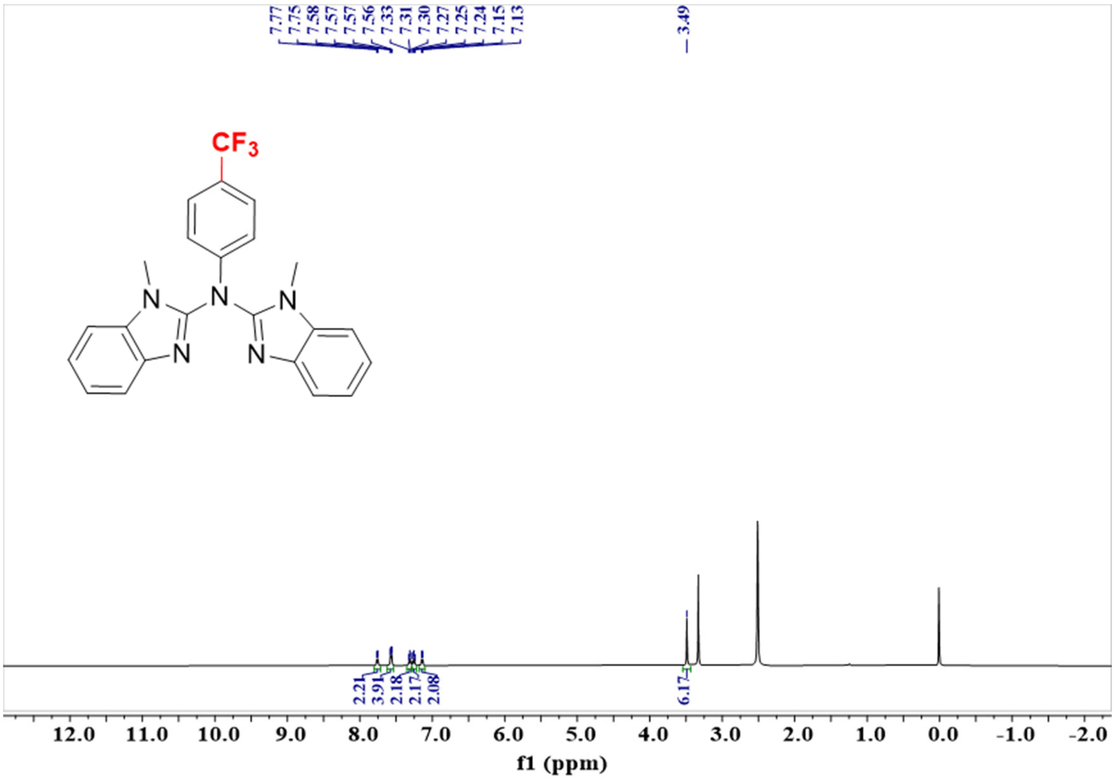

Figure S7 ^{1}H-NMR spectrum of **L$_4$**

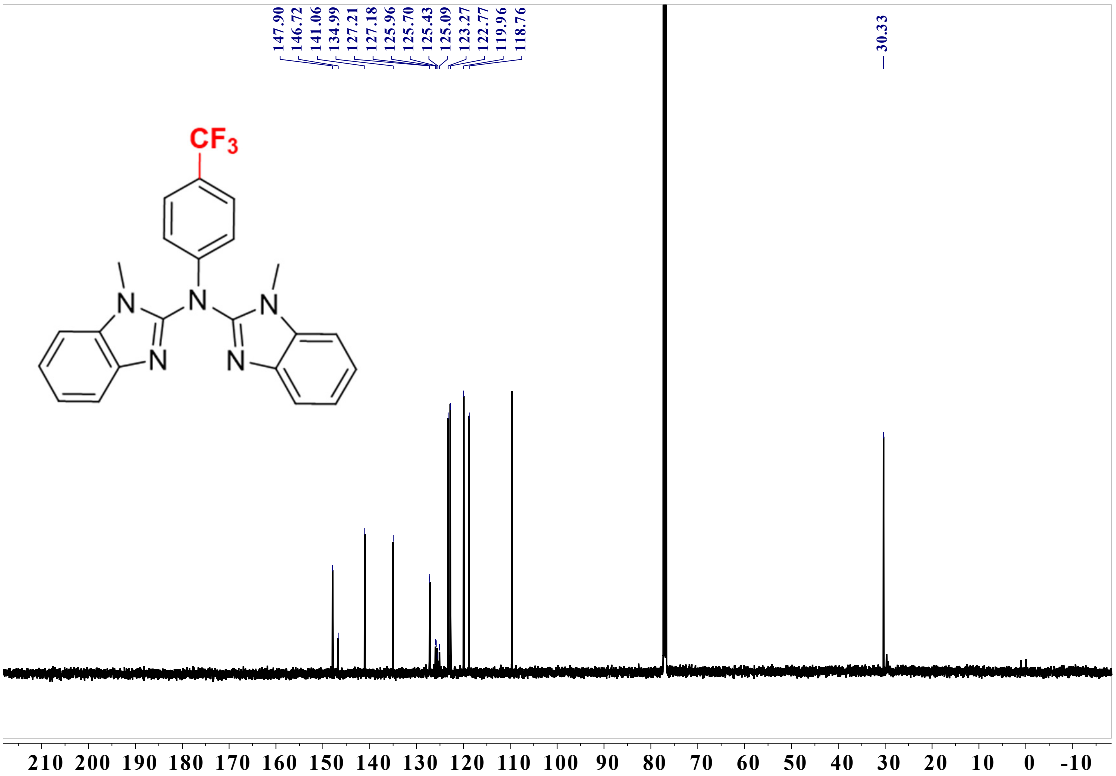

Figure S8 ^{13}C-NMR spectrum of **L$_4$**

2.1.5 L_5

^{1}H NMR (500 MHz, DMSO-d$_6$) δ 7.55 (t, *J* = 8.5 Hz, 4H), 7.42 (t, *J* = 7.8 Hz, 2H), 7.33-7.15 (m, 5H), 7.03 (d, *J* = 7.8 Hz, 2H), 4.00 (q, *J* = 7.2 Hz, 4H), 1.08 (t, *J* = 7.2 Hz, 6H).

^{13}C NMR (126 MHz, CDCl$_3$) δ 148.6, 144.4, 141.5, 134.2, 129.7, 124.3, 122.7, 122.3, 120.3, 119.9, 109.69, 39.0, 14.1.

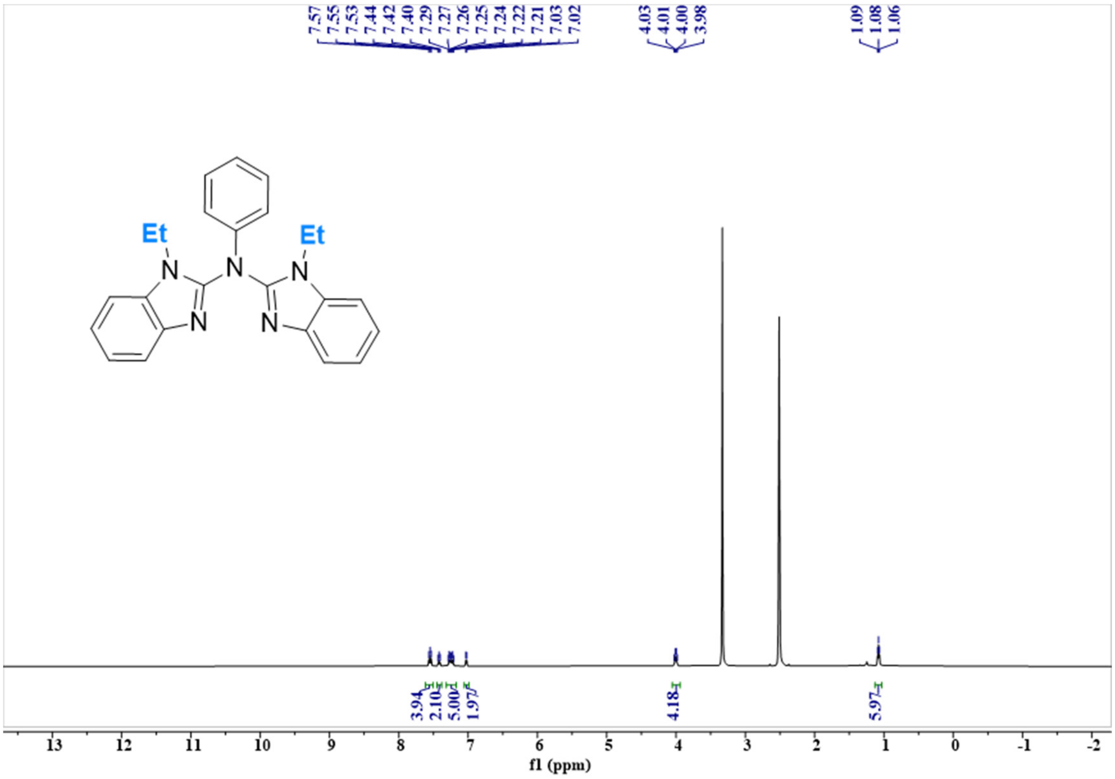

Figure S9 ^{1}H-NMR spectrum of **L_5**

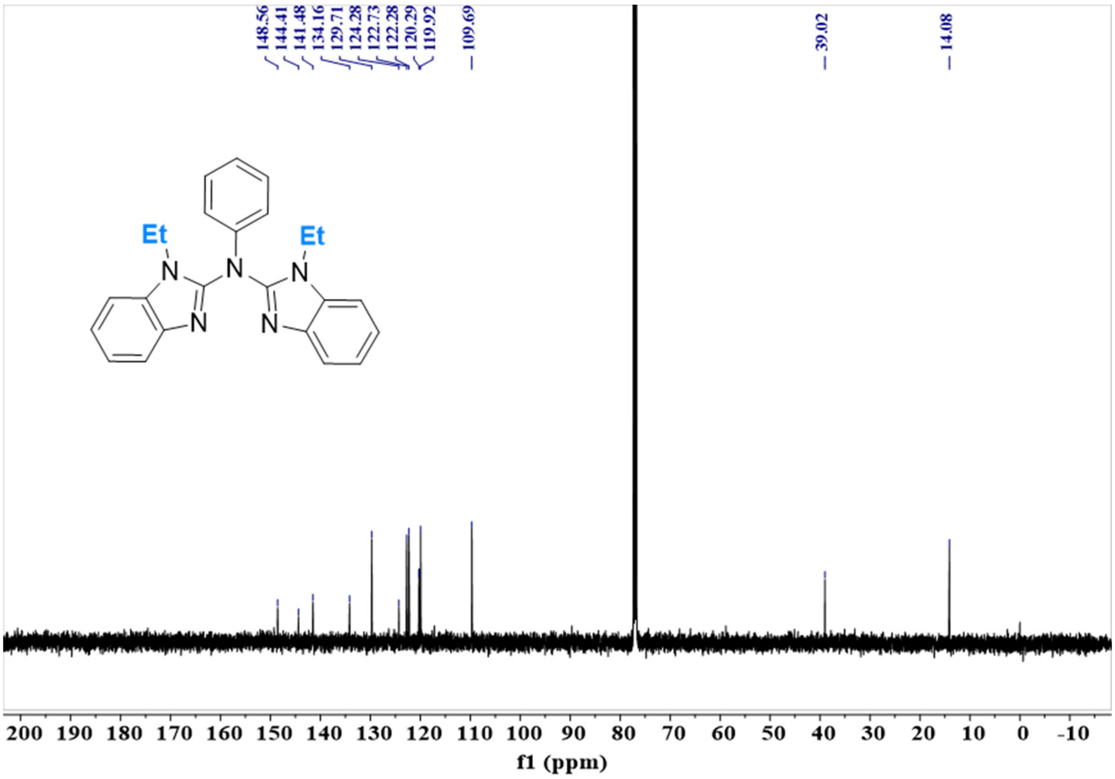

Figure S10 ^{13}C-NMR spectrum of **L_5**

2.1.6 L_6

^{1}H NMR (500 MHz, DMSO-d_6) δ 7.74 (d, *J* = 7.8 Hz, 2H), 7.55 (d, *J* = 7.5 Hz, 2H), 7.39 (t, *J* = 7.8 Hz, 2H), 7.28-7.16(m, 5H), 6.91 (d, *J* = 7.9 Hz, 2H), 4.65 (p, *J* = 6.9 Hz, 2H), 1.40 (d, *J* = 6.9 Hz, 12H).

^{13}C NMR (126 MHz, $CDCl_3$) δ 147.7, 145.1, 142.0, 132.5, 129.5, 123.2, 122.5, 121.9, 120.3, 118.1, 112.1, 48.2, 20.8.

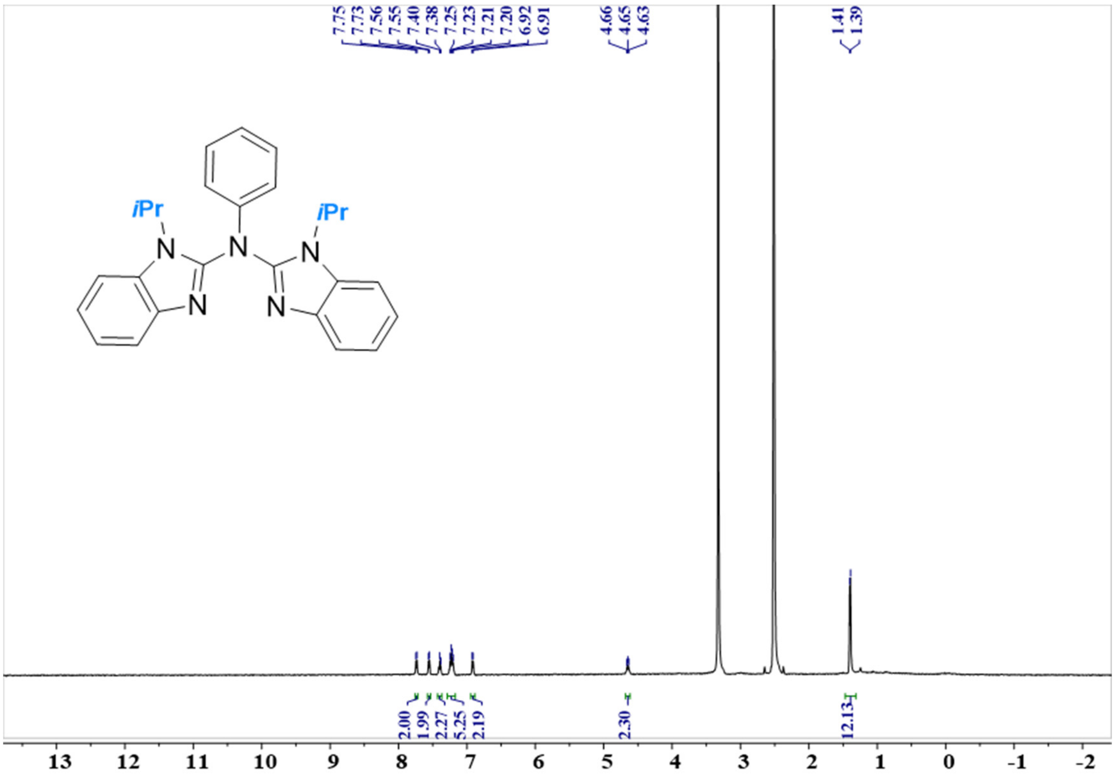

Figure S11 ^{1}H-NMR spectrum of $\mathbf{L_6}$

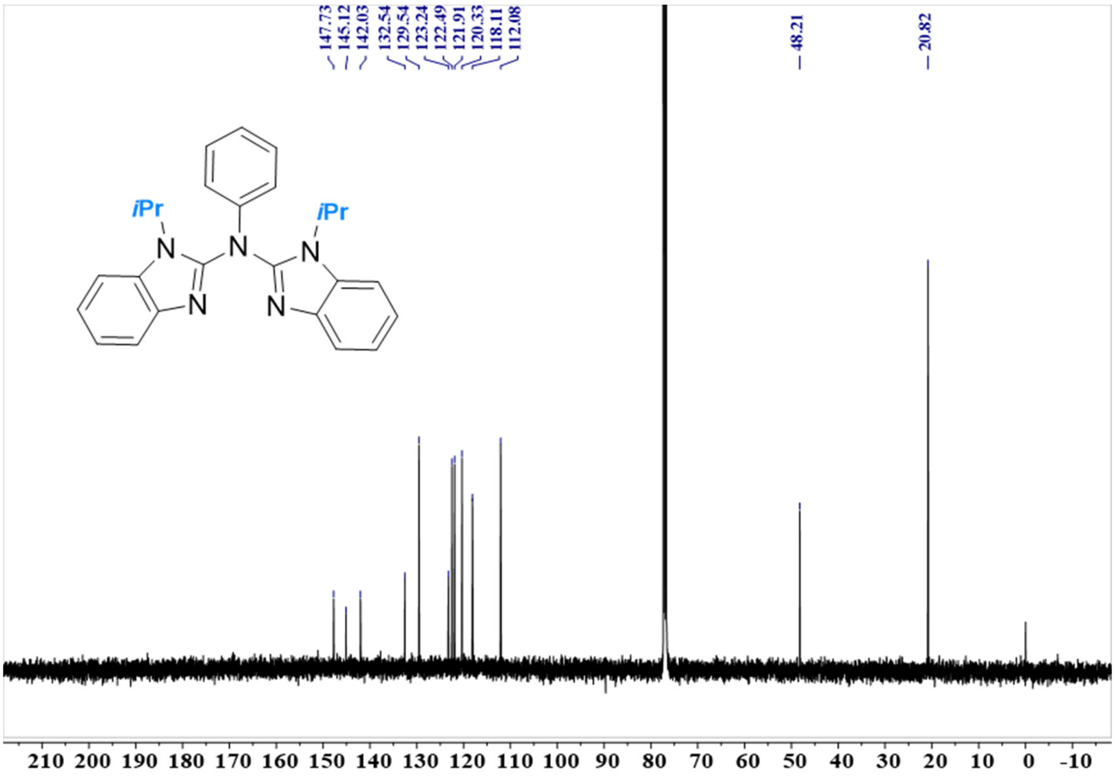

Figure S12 ^{13}C-NMR spectrum of $\mathbf{L_6}$

2.1.7 L$_7$

^{1}H NMR (500 MHz, DMSO-d$_6$) δ 7.50 (d, *J* = 7.9 Hz, 4H), 7.29 – 7.17 (m, 6H), 6.97 – 6.93 (m, 2H), 3.40 (s, 6H), 2.34 (s, 3H).

^{13}C NMR (126 MHz, CDCl$_3$) δ 149.8, 141.2, 140.8, 135.1, 134.7, 130.5, 122.5, 122.3, 121.3, 119.6, 109.1, 30.3, 20.9.

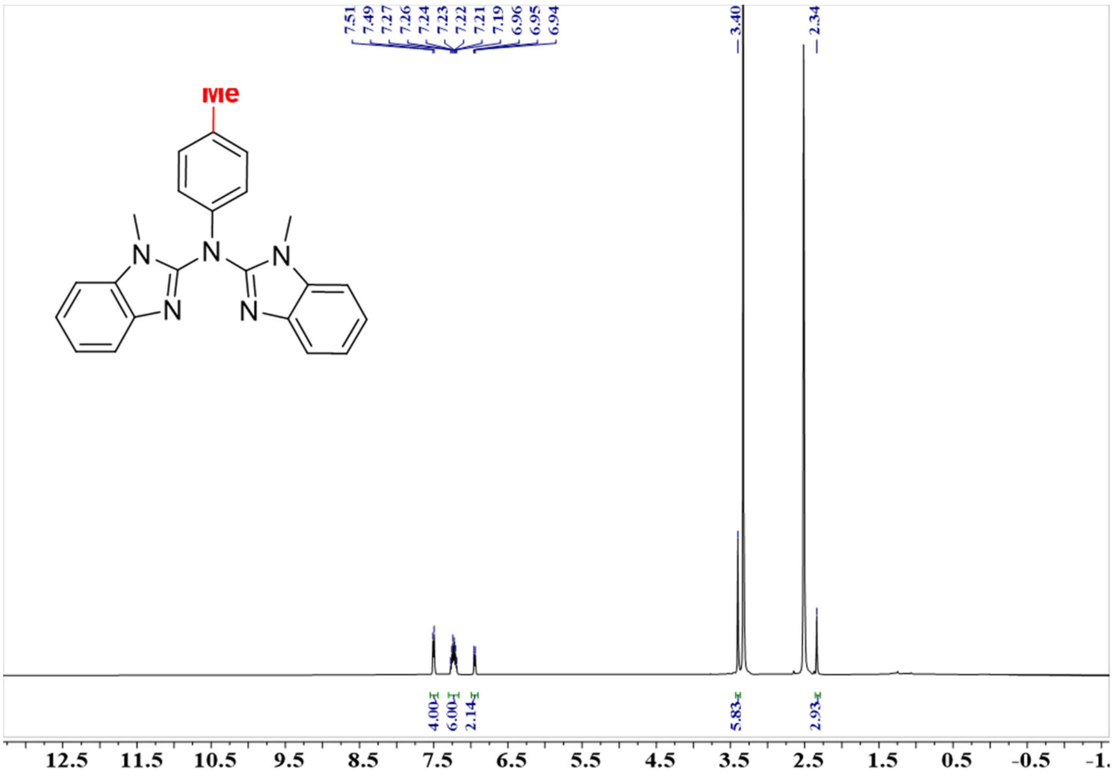

Figure S13 ^{1}H-NMR spectrum of **L$_7$**

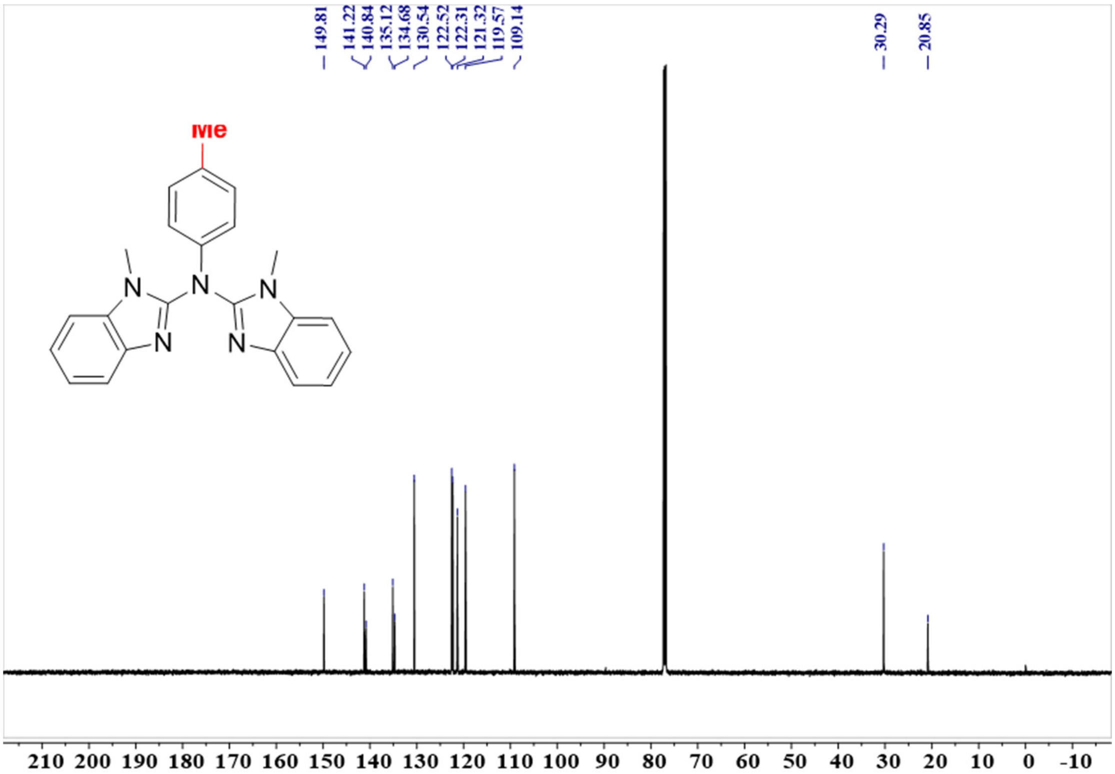

Figure S14 ^{13}C-NMR spectrum of **L**$_7$

2.2 Characteristic data of [Ru-1] - [Ru-9] (NMR and HR-MS)

2.2.1 [Ru-1]

^{1}H NMR (500MHz, CDC1$_3$) δ 7.92 (d, *J*=7.9Hz, 2H), 7.66 (d, *J*=8.0Hz, 2H), 7.62 (t, *J*=7.4Hz, 2H), 7.51 (t, *J*=7.5Hz, 2H), 7.47 (t, *J*=7.5Hz, 2H), 7.32-7.28 (m, 1H), 6.98 (d, *J*=7.9Hz, 2H), 5.68 (d, *J*=5.6Hz, 2H), 5.42 (d, *J*=5.4Hz, 2H), 4.13 (s, 6H), 2.29-2.23 (m, 1H), 1.92 (s, 3H), 1.06 (d, *J*=6.8Hz, 6H).

^{13}C NMR (126 MHz, CDCl$_3$) δ 144.9, 142.3, 139.0, 133.7, 131.5, 125.9, 125.5, 125.0, 119.7, 114.1, 112.2, 106.0, 100.7, 83.0, 82.7, 32.2, 30.9, 30.8, 22.7, 18.8.

HR-MS (ESI positive): m/z calcd. for $C_{32}H_{33}ClN_5Ru$ $[M]^+$: 624.17 ; found: 624.14724.

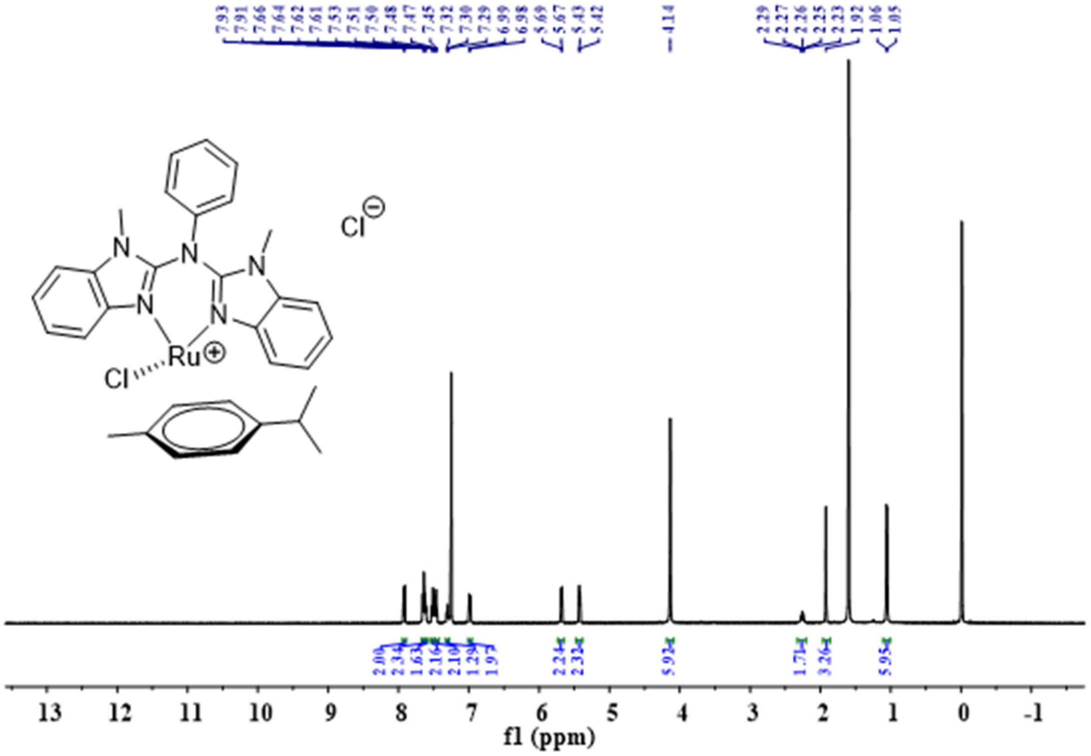

Figure S15 ^{1}H-NMR spectrum of **[Ru-1]**

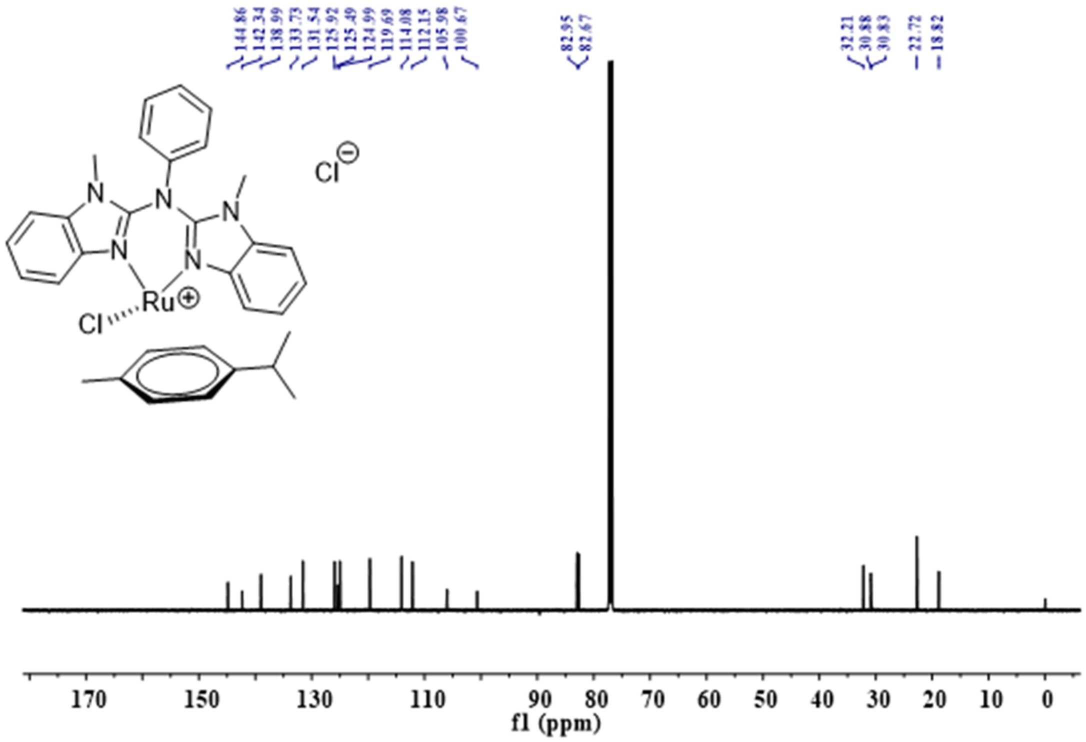

Figure S16 ^{13}C-NMR spectrum of **[Ru-1]**

2.2.2 [Ru-2]

^{1}H NMR (500 MHz, $CDCl_3$) δ 7.92 (d, J = 7.9 Hz, 2H), 7.62 (d, J = 7.9 Hz, 2H), 7.53 – 7.40 (m, 4H), 6.87 (d, J = 9.2 Hz, 2H), 6.81 (d, J = 9.2 Hz, 2H), 5.70 (d, J = 5.9 Hz, 2H), 5.43 (d, J = 5.9 Hz, 2H), 4.08 (s, 6H), 2.95 (s, 6H), 2.34 (dt, J = 13.6, 6.8 Hz, 1H), 1.90 (s, 3H), 1.08 (d, J = 6.9 Hz, 6H).

^{13}C NMR (126 MHz, $CDCl_3$) δ 148.4, 145.6, 139.1, 133.8, 132.0, 125.8, 124.9, 119.7, 115.5, 114.4, 112.0, 105.8, 100.8, 83.3, 82.6, 40.7, 32.1, 30.8, 22.8, 18.9.

HR-MS (ESI positive): m/z calcd. for $C_{34}H_{38}ClN_6Ru$ $[M]^+$: 667.24 ; found: 667.18958.

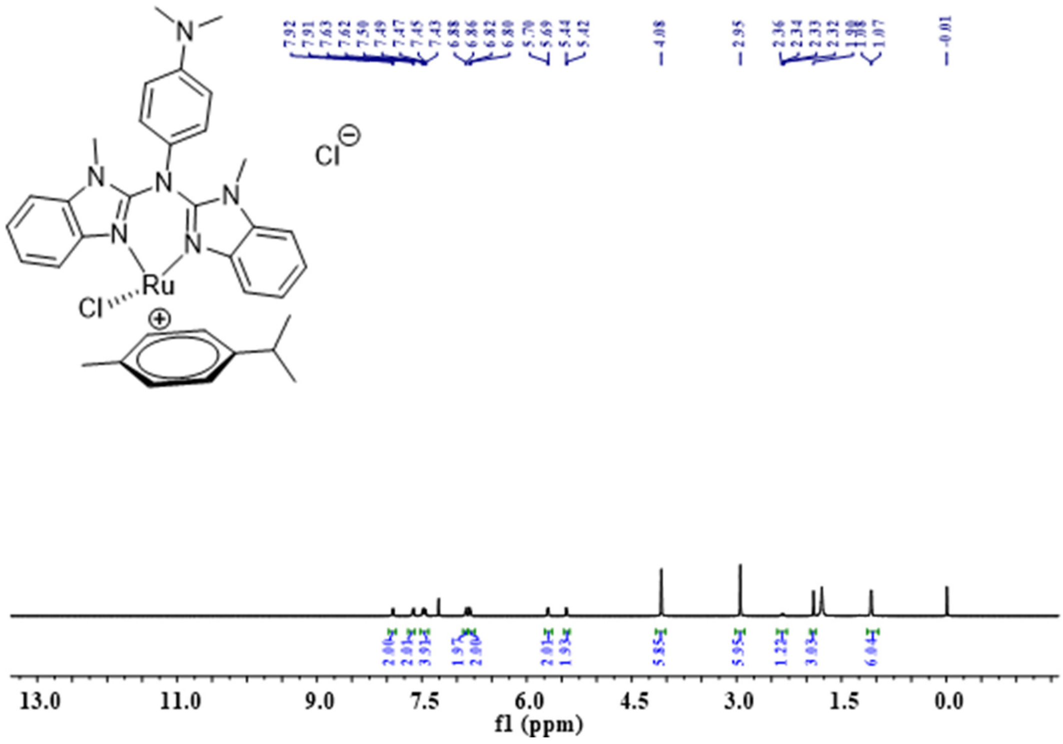

Figure S17 ^{1}H-NMR spectrum of **[Ru-2]**

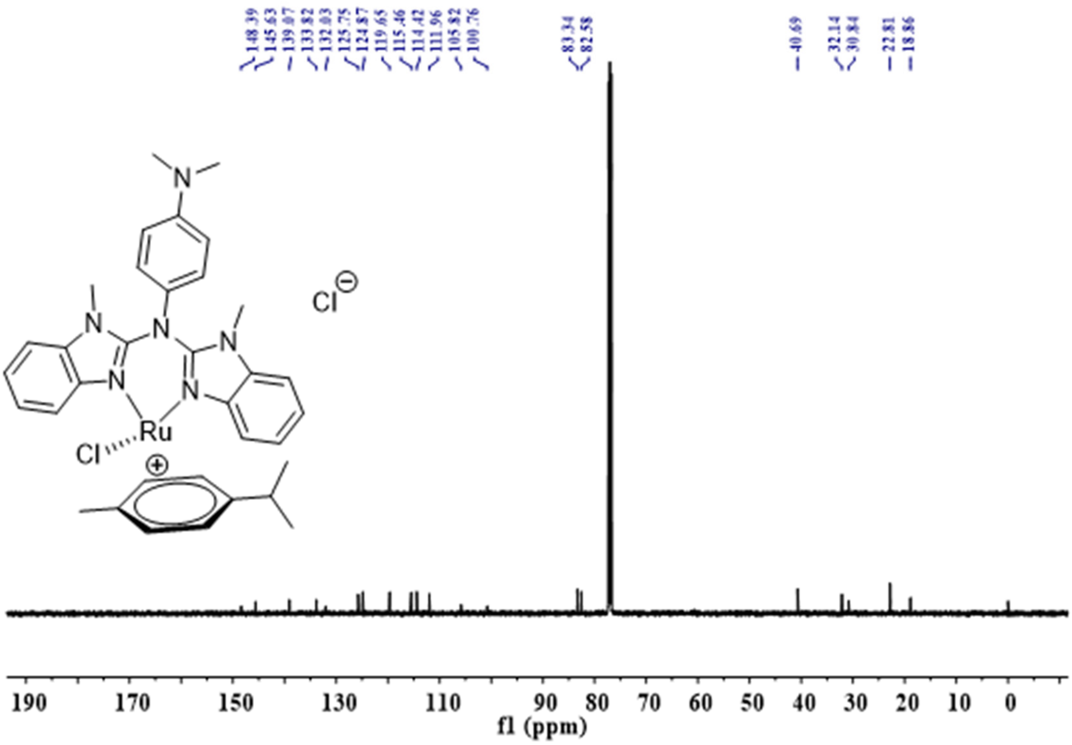

Figure S18 ^{13}C-NMR spectrum of **[Ru-2]**

2.2.3 [Ru-3]

^{1}H NMR (500 MHz, $CDCl_3$) δ 7.92 (d, *J* = 8.2 Hz, 2H), 7.63 (d, *J* = 8.1 Hz, 2H), 7.56-7.45 (m, 4H), 7.40-7.33 (m, 2H), 7.19-7.12 (m, 2H), 5.77 (d, *J* = 5.7 Hz, 2H), 5.50 (d, *J* = 5.8 Hz, 2H), 4.12 (s, 6H), 2.37-2.28 (m, 1H), 1.94 (s, 3H), 1.09 (d, *J* = 6.9 Hz, 6H).

^{19}F NMR (471 MHz, $CDCl_3$) δ -116.38 (d, *J* = 20.8 Hz).

^{13}C NMR (126MHz, $CDCl_3$) δ 159.7 (d, J=246 6Hz), 144.9, 139.0, 138.4 (d, J=2.6Hz), 133.8, 125.8, 124.9, 119.7, 118.6 (d, J=23.3Hz), 116.3 (d, J=7.9Hz), 112.3, 106.1, 100.4, 82.9, 82.9, 32.5, 30.9, 22.7, 18.8.

HR-MS (ESI positive): m/z calcd. for $C_{34}H_{38}ClFN_5Ru$ $[M]^+$: 642.16 ; found: 642.1375**.**

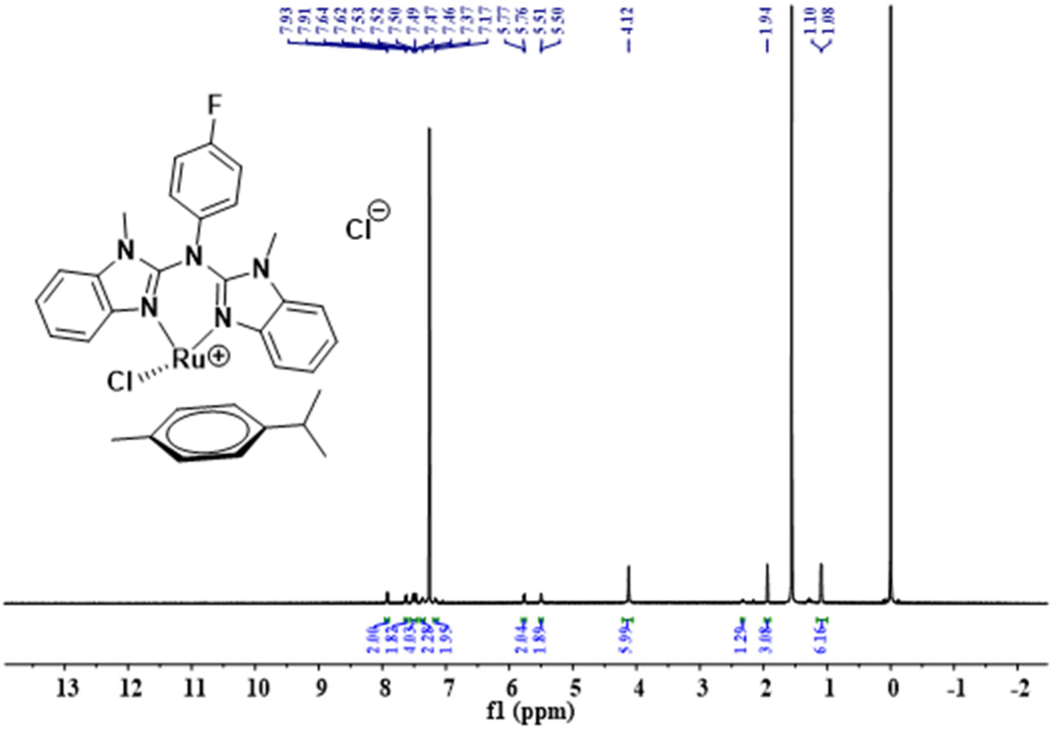

Figure S19 ^{1}H-NMR spectrum of **[Ru-3]**

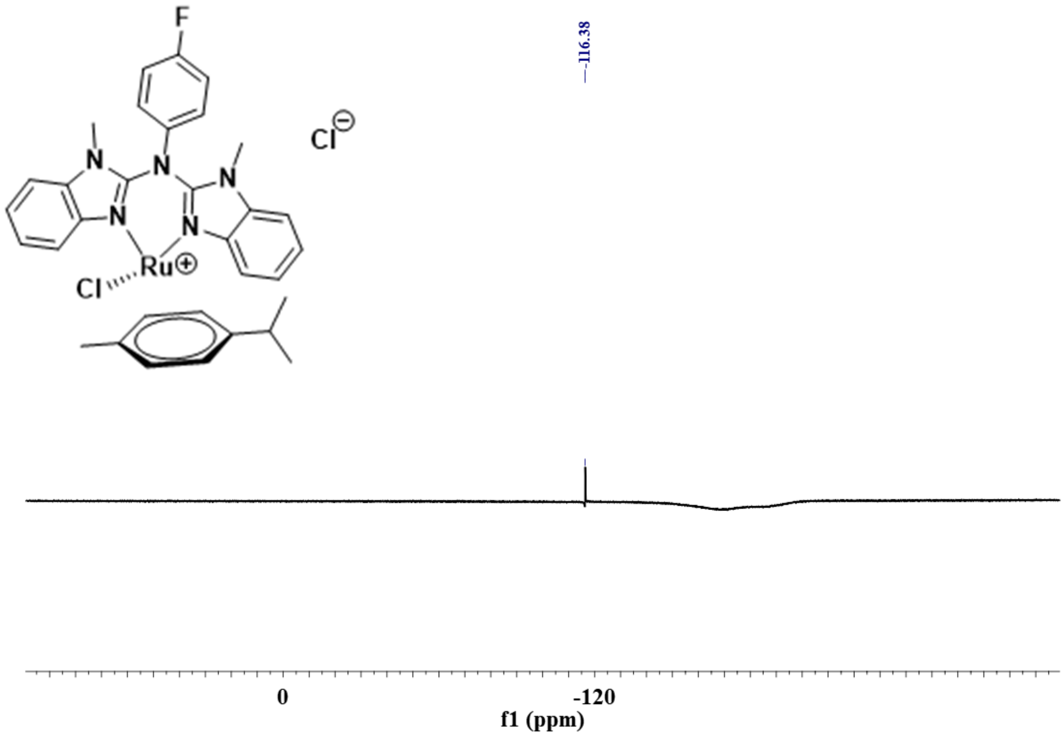

Figure S20 ^{19}F-NMR spectrum of **[Ru-3]**

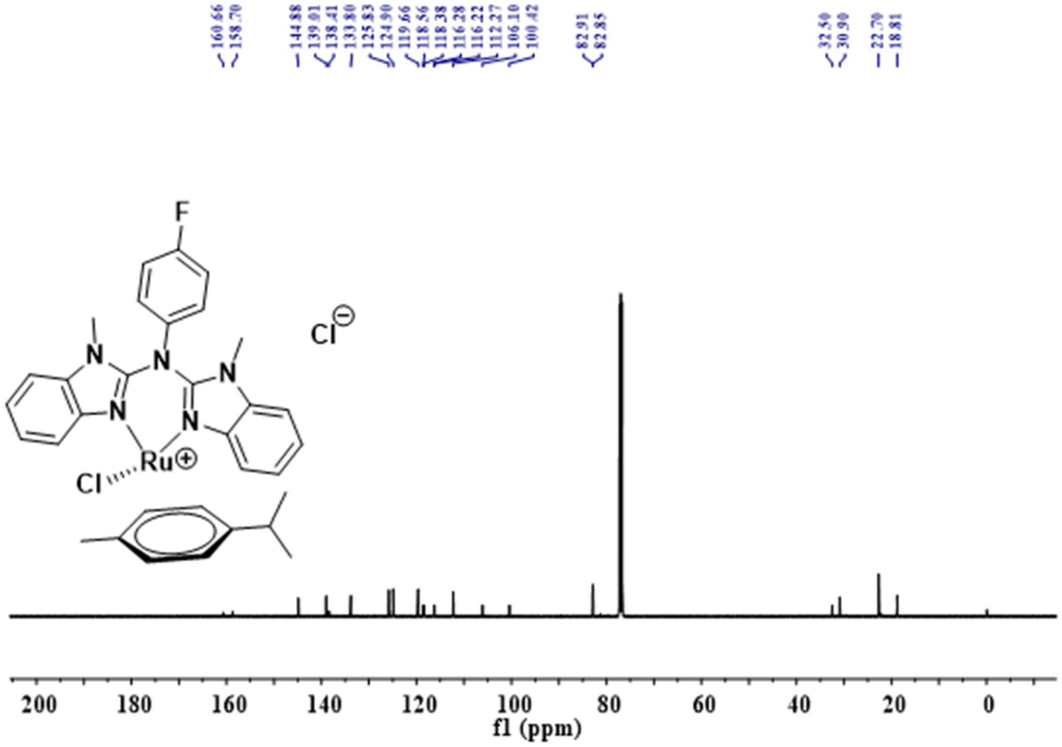

Figure S21 ^{13}C-NMR spectrum of **[Ru-3]**

2.2.4 [Ru-4]

^{1}H NMR (500 MHz, DMSO-d_6) δ 7.92 (d, J = 7.9 Hz, 2H), 7.88 – 7.77 (m, 4H), 7.54 (t, J = 7.3 Hz, 2H), 7.48 (t, J = 7.3 Hz, 2H), 7.30 (d, J = 8.0 Hz, 2H), 5.83 (d, J = 5.2 Hz, 2H), 5.62 (d, J = 5.2 Hz, 2H), 3.99 (s, 6H), 2.08 – 1.95 (m, 1H), 1.91 (s, 3H), 0.94 (d, J = 6.4 Hz, 6H).

^{19}F NMR (471 MHz, $CDCl_3$) δ -62.11.

^{13}C NMR (126MHz, $CDCl_3$) δ 145.0, 144.2, 139.1, 133.9, 128.9 (q,J=3.6Hz),1 27.6 (q, J=33.7Hz), 126.1, 125.1, 123.5 (q, J=272.1Hz), 119.8, 115.1, 112.2, 106.2, 100.4, 83.0, 82.8, 32.2, 30.9, 22.7, 18.8.

HR-MS (ESI positive): m/z calcd. for $C_{33}H_{32}ClF_3N_5Ru$ $[M]^+$: 692.17 ; found: 692.13449.

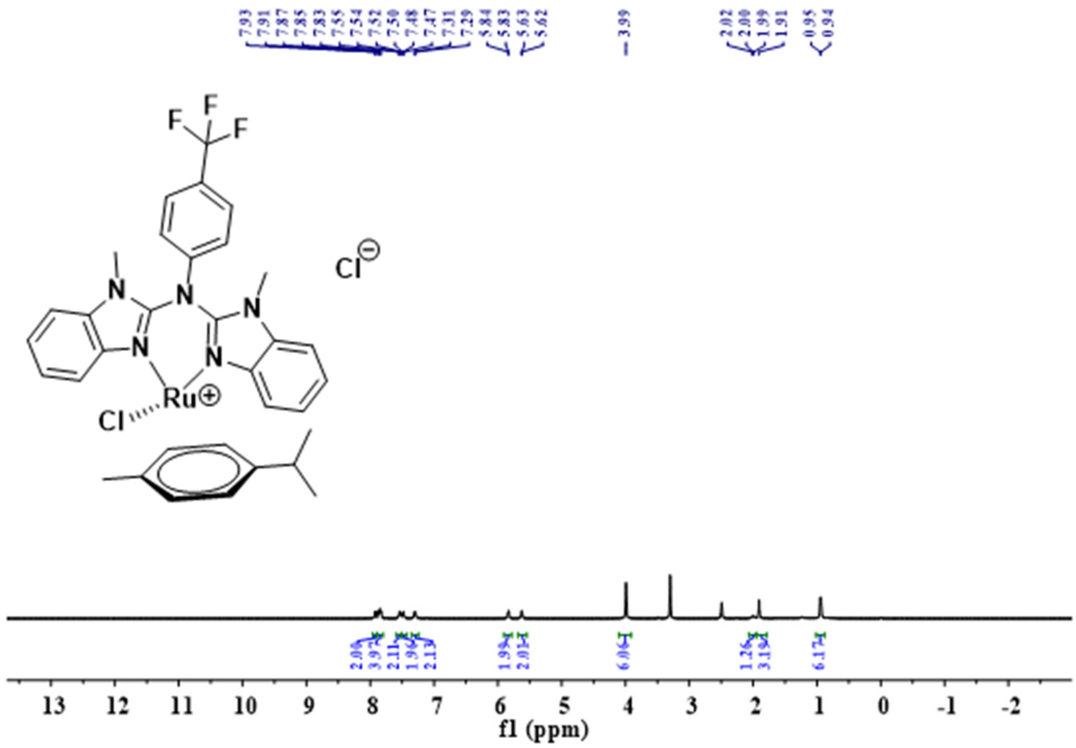

Figure S22 ^{1}H-NMR spectrum of **[Ru-4]**

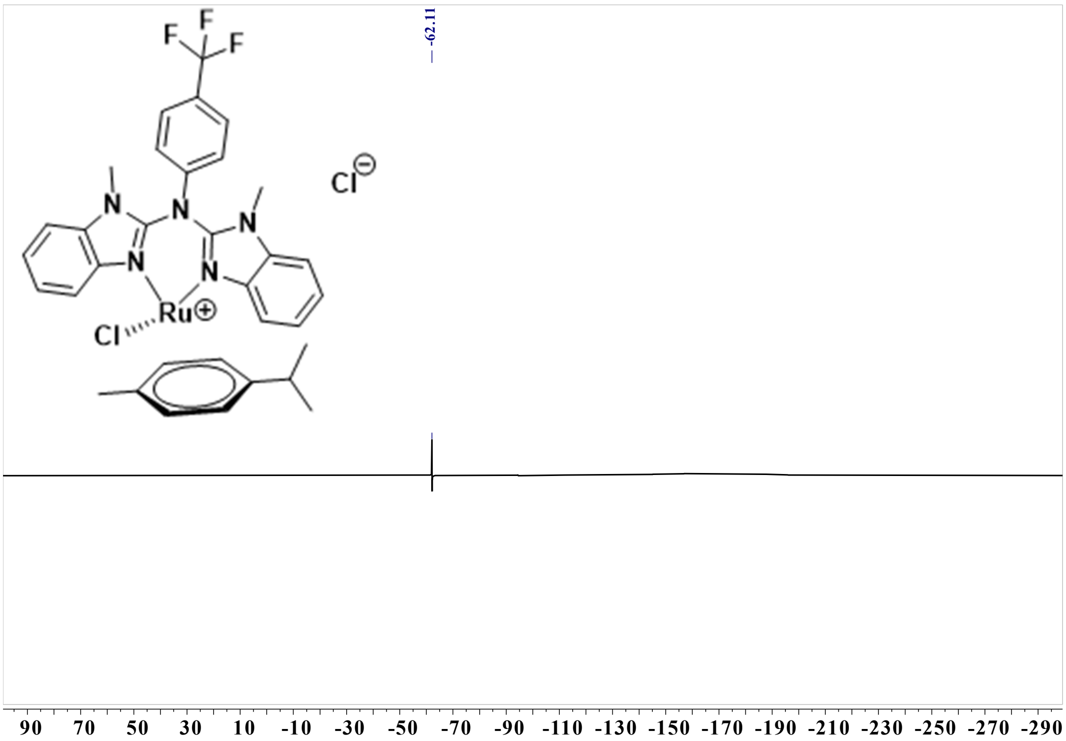

Figure S23 ^{19}F-NMR spectrum of **[Ru-4]**

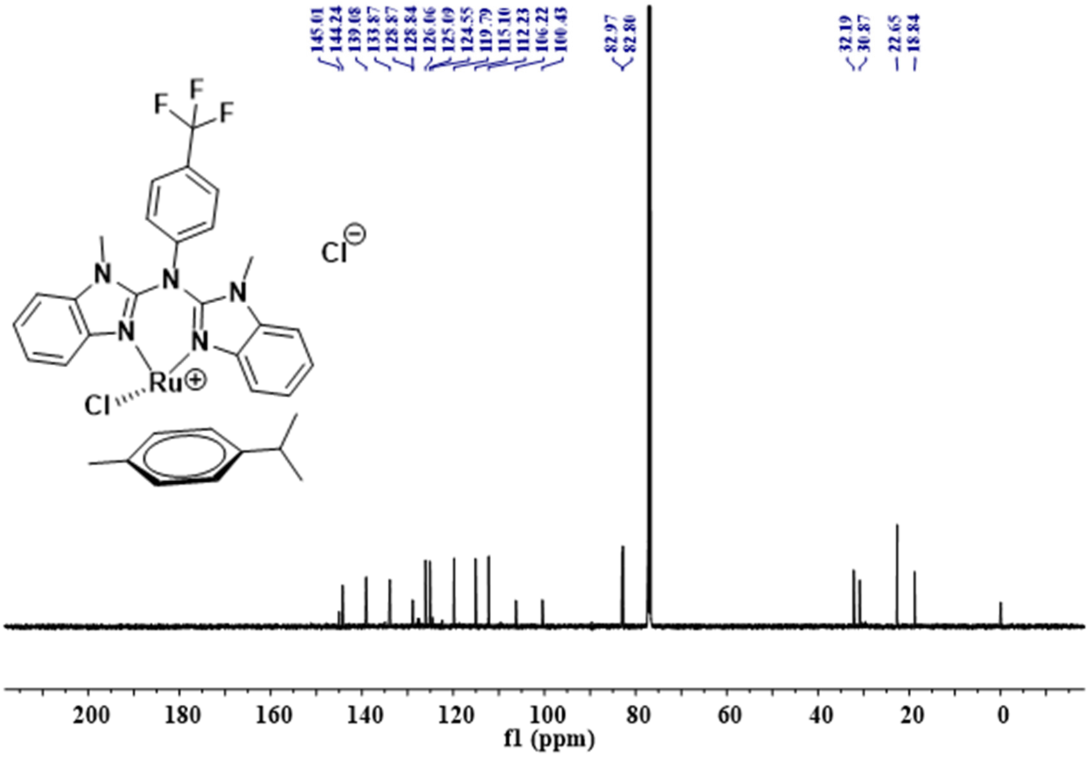

Figure S24 ^{13}C-NMR spectrum of **[Ru-4]**

2.2.5 [Ru-5]

^{1}H NMR (500MHz, $CDCl_3$,) δ 7.96 (d, *J*=8.0Hz, 2H), 7.69 (d, *J*=7.9Hz, 2H), 7.61 (t, *J*=7.3Hz,2H), 7.51 (t, *J*=7.5Hz,2H),7.49-7.44 (m,2H), 7.30(t, *J*=7.0Hz,1H), 6.93 (d, *J*=7.7Hz,2H), 5.64 (d, *J*=5.5Hz, 2H), 5.32 (d, *J*=5.5Hz, 2H), 4.81-4.68 (m, 2H), 4.56-4.39 (m, 2H), 2.27-2.17 (m,1H), 1.88 (s, 3H), 1.52 (t, *J*=6.8Hz, 6H), 1.05 (d, *J*=6.7Hz, 6H).

^{13}C NMR (126 MHz, $CDCl_3$) δ 144.3, 143.0, 139.5, 132.7, 131.6, 126.0, 125.8, 125.0, 120.1, 114.5, 112.6, 105.8, 101.3, 83.6, 82.4, 41.4, 30.9, 22.9, 19.0, 14.9.

HR-MS (ESI positive): m/z calcd. for $C_{34}H_{37}ClN_5Ru$ $[M]^+$: 652.22 ; found: 652.1758.

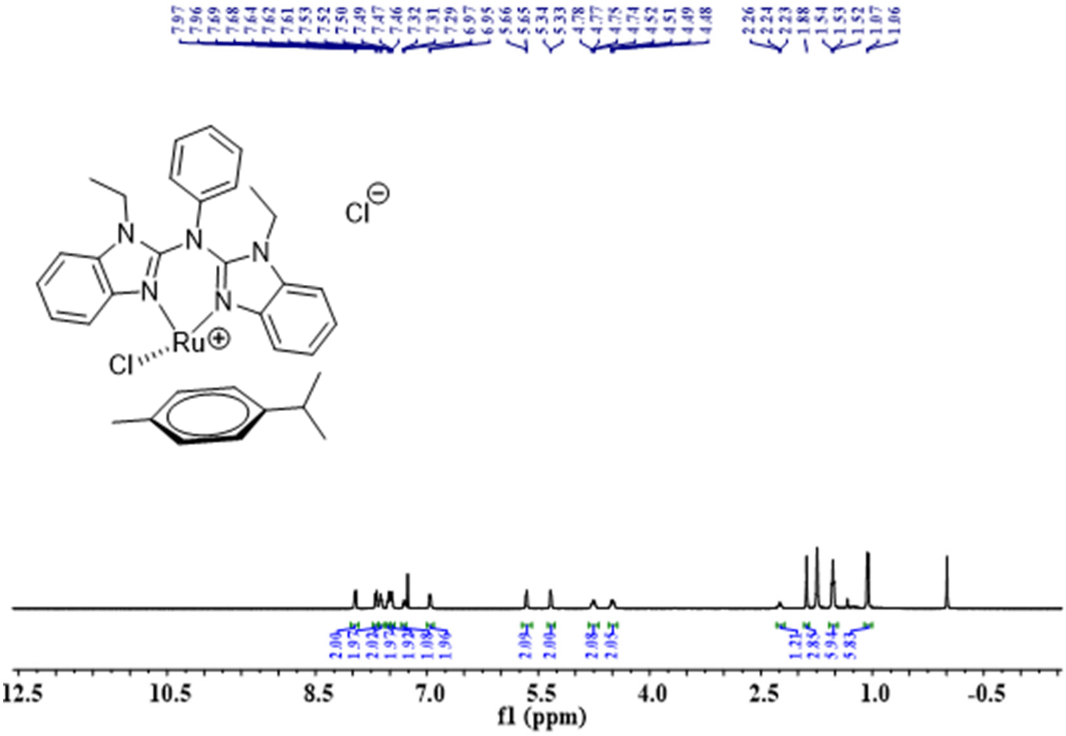

Figure S25 ^{1}H-NMR spectrum of **[Ru-5]**

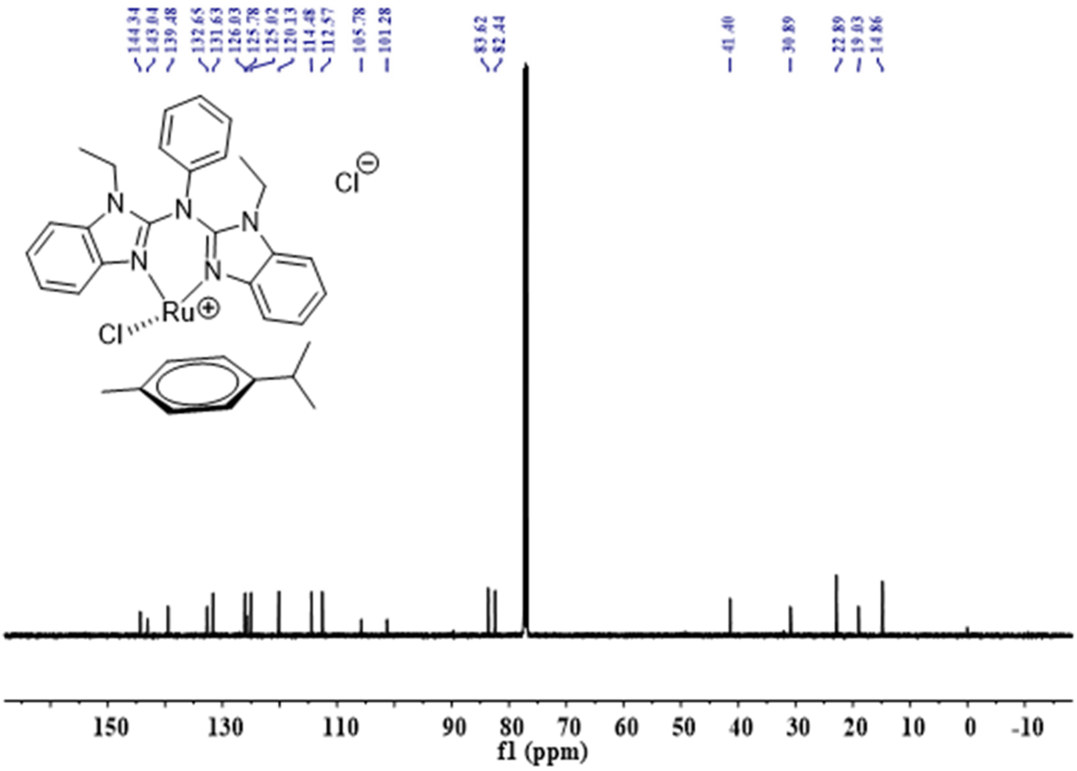

Figure S26 ^{13}C-NMR spectrum of **[Ru-5]**

2.2.6 [Ru-6]

^{1}H NMR(500MHz, $CDCl_3$) δ 88.07-7.92 (m, 2H), 7.76-7.68 (m, 2H), 7.64 (t, *J*=7.7Hz, 2H), 7.52-7.40 (m, 4H), 7.34 (t, *J*=7.3Hz, 1H), 6.90 (d, *J*=8.1Hz,2H), 5.68 (d, *J*=5.7Hz2H), 5.32 (d, *J*=5.7Hz, 2H), 5.12-5.00 (m,2H), 2.38-2.23 (m,1H), 2.01 (d, *J*=6.8Hz, 6H), 1.89 (s, 3H), 1.54 (d, *J*=6.8Hz, 6H), 1.10 (d, *J*=6.8Hz, 6H).

^{13}C NMR (126MHz, $CDCl_3$) δ 8143.8, 143.5, 139.7, 131.4, 131.2, 125.8, 125.6, 124.5, 120.7, 114.1, 113.5, 105.5, 101.4, 83.8, 82.1, 50.8, 30.8, 22.8, 21.9, 21.1, 19.1.

HR-MS (ESI positive): m/z calcd. for $C_{36}H_{41}ClN_5Ru$ $[M]^+$: 680.28 ; found: 680.2097.

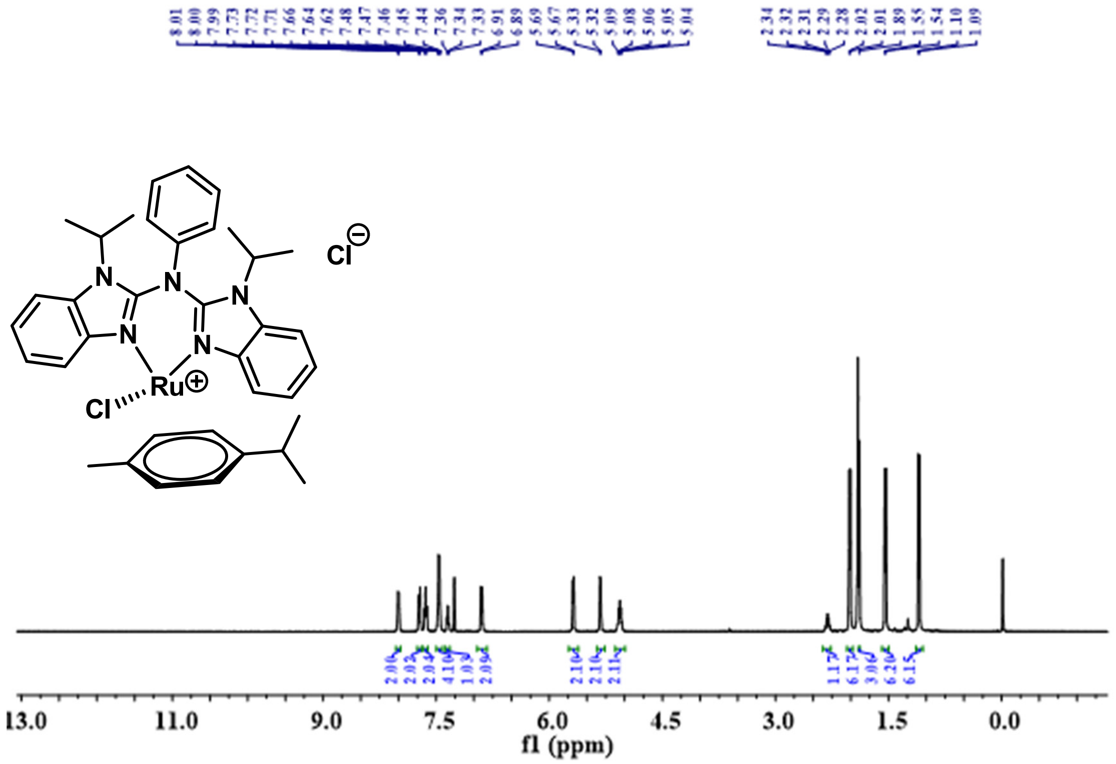

Figure S27 ^{1}H-NMR spectrum of **[Ru-6]**

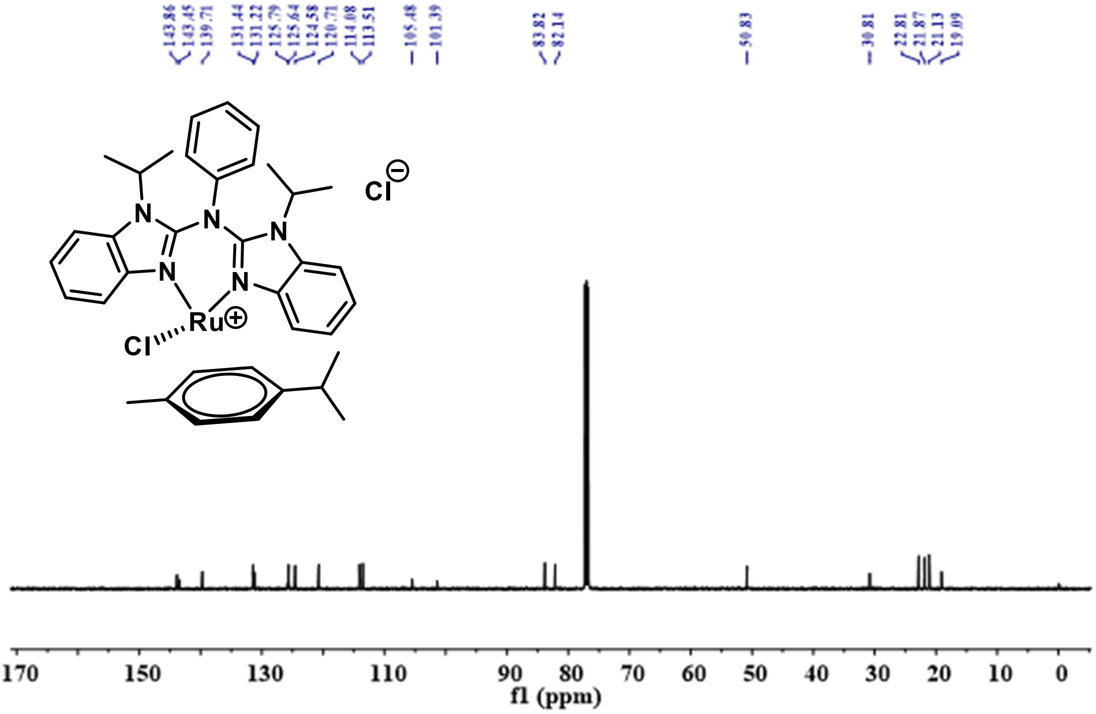

Figure S28 ^{13}C-NMR spectrum of **[Ru-6]**

2.2.7 [Ru-7]

^{1}H NMR(500MHz, $CDCl_3$) δ 7.91 (d, *J*=8.0Hz, 2H), 7.66 (d, *J*=7.8Hz, 2H), 7.49 (t, *J*=7.5Hz, 2H), 7.45 (t, *J*=7.6Hz, 2H), 7.37 (d, *J*=7.5Hz, 2H), 6.80 (d, *J*=7.6Hz, 2H), 5.66 (d, *J*=5.4Hz, 2H), 5.39 (d, *J*=5.3Hz, 2H), 4.10 (s, 6H), 2.36 (s, 3H), 2.32-2.24 (m, 1H), 1.88 (s, 3H), 1.06 (d, *J*=6.7Hz, 6H).

^{13}C NMR (126MHz, $CDCl_3$) δ 145.1, 140.0, 139.0, 135.4, 133.8, 131.9, 125.9, 124.9, 119.7, 114.1, 112.2, 105.8, 100.8, 83.3, 82.5, 32.3, 30.8, 22.7, 20.6, 18.9.

HR-MS (ESI positive): m/z calcd. for $C_{33}H_{35}ClN_5Ru$ $[M]^+$: 638.20 ; found: 638.16265.

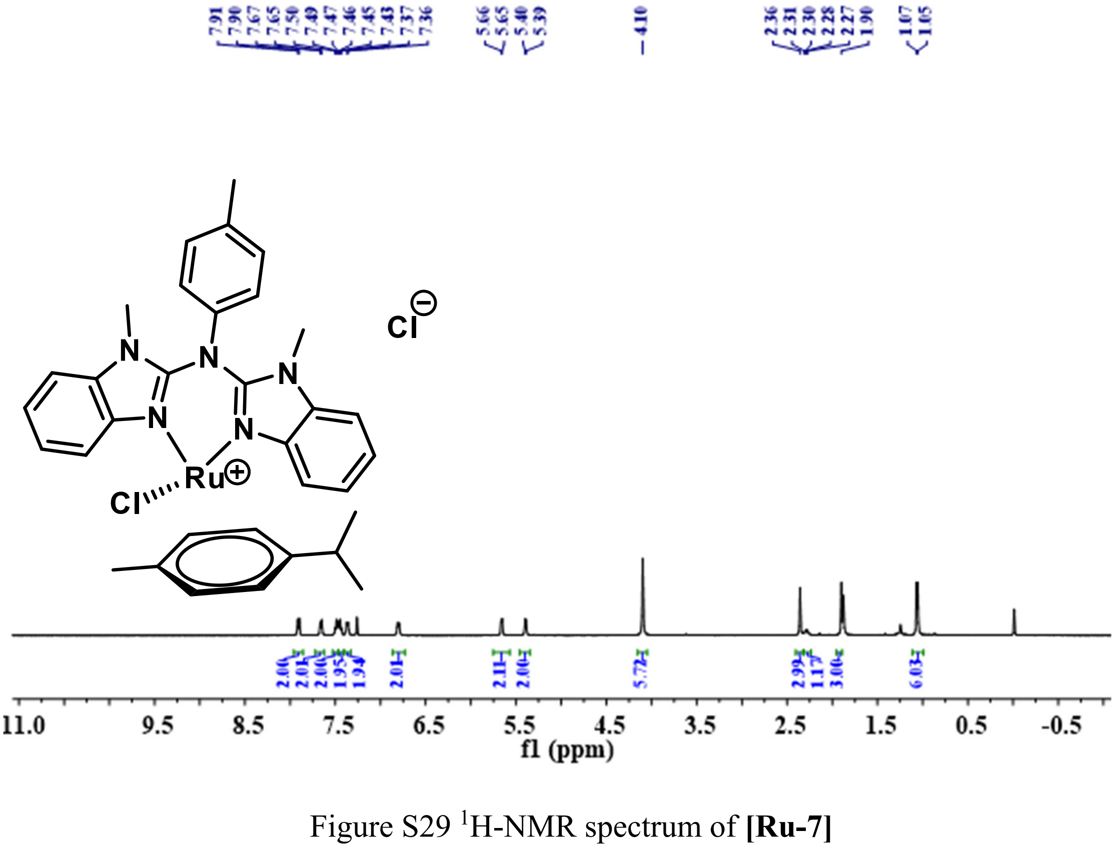

Figure S29 ^{1}H-NMR spectrum of **[Ru-7]**

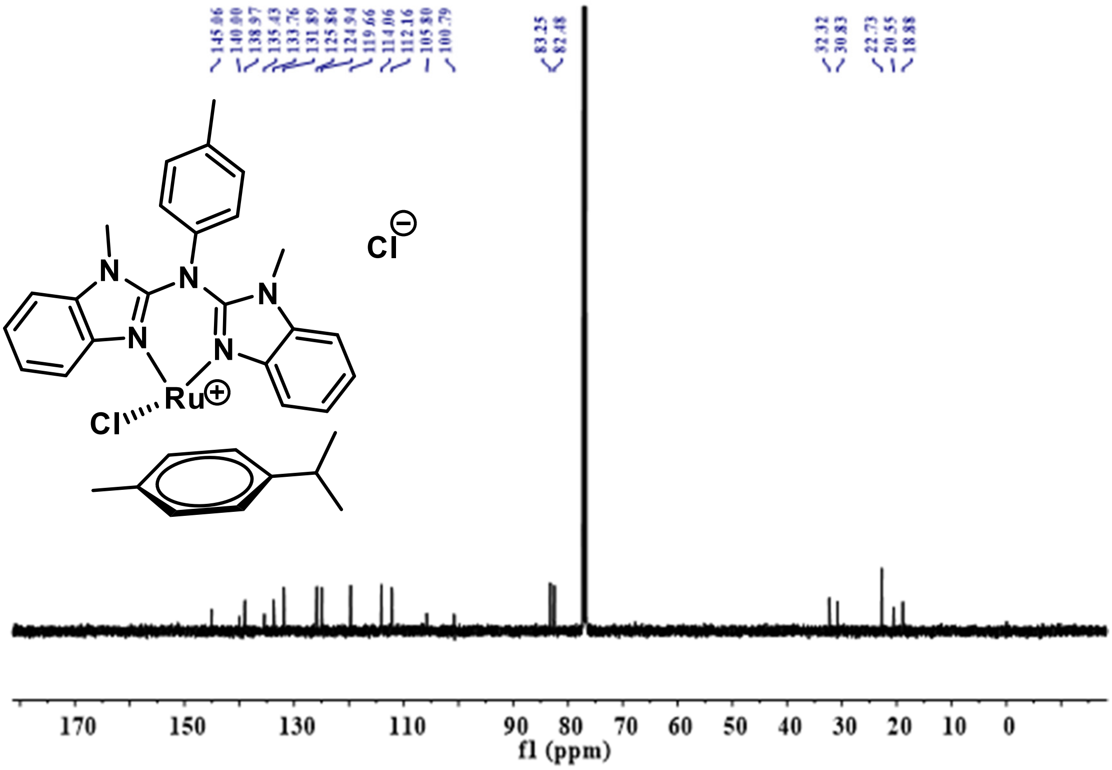

Figure S30 ^{13}C-NMR spectrum of **[Ru-7]**

2.2.8 [Ru-8]

^{1}H NMR (500 MHz, $CDCl_3$) δ 7.71 (d, *J*=8.1Hz, 2H), 7.67-7.55 (m, 4H), 7.46 (t, *J*=7.5Hz, 2H), 7.41 (t, *J*=7.6Hz, 2H), 7.30 (t, *J*=7.4Hz, 1H), 7.02 (d, *J*=8.0Hz, 2H), 4.02 (s, 6H), 1.96 (s, 18H).

^{13}C NMR (126 MHz, $CDCl_3$) δ 144.3, 139.9, 137.5, 134.2, 130.9, 125.6, 125.0, 124.2, 120.2, 114.0, 112.1, 93.6, 89.5, 32.6, 16.4, 15.8.

HR-MS (ESI positive): m/z calcd. for $C_{28}H_{25}ClN_5Ru$ $[M]^+$: 568.06 ; found: 568.0413.

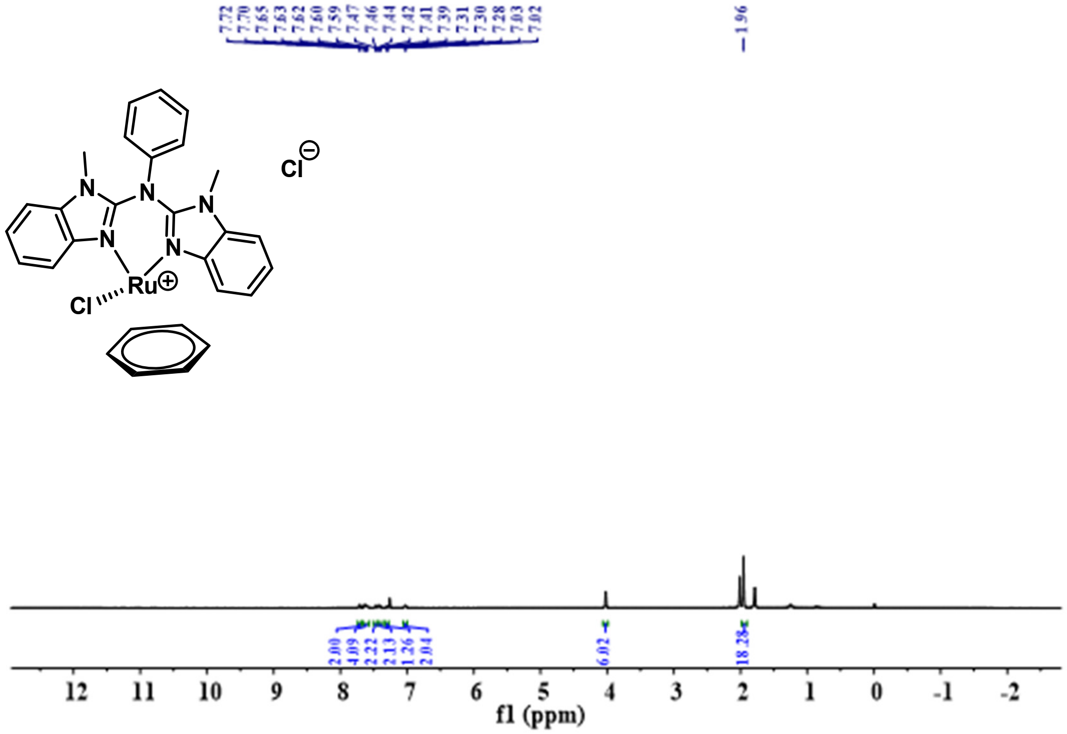

Figure S31 ^{1}H-NMR spectrum of **[Ru-8]**

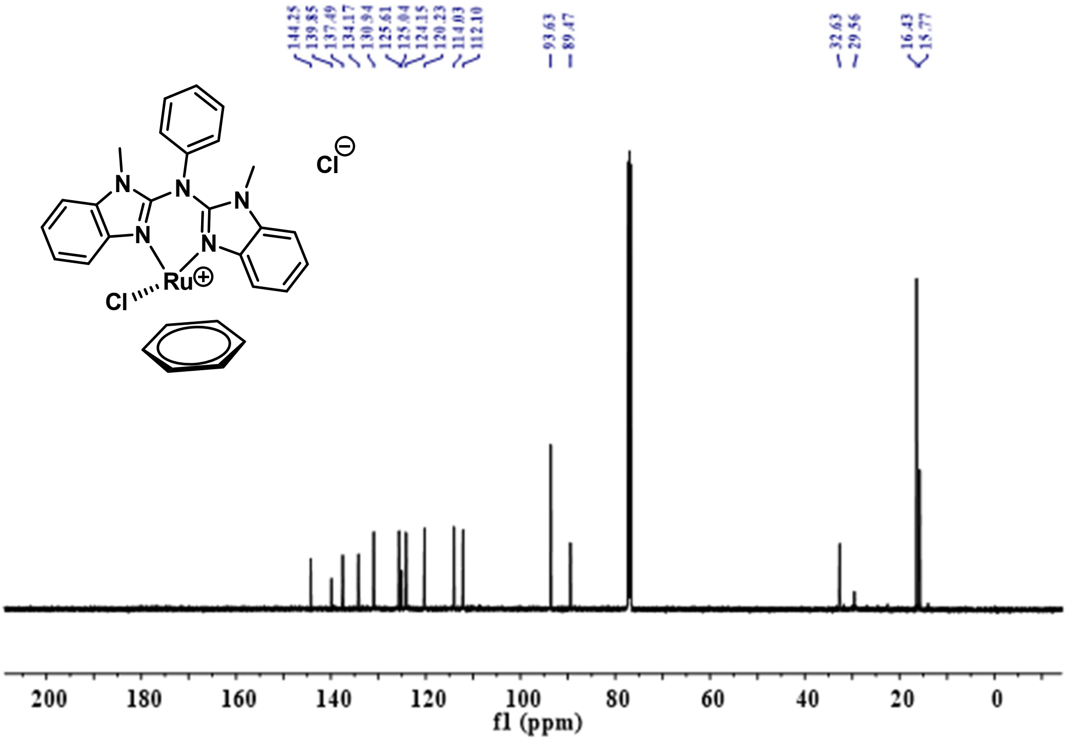

Figure S32 ^{13}C-NMR spectrum of **[Ru-8]**

2.2.9 [Ru-9]

^{1}H NMR (500 MHz, DMSO-d_6) δ 7.98 (d, *J* = 8.2 Hz, 2H), 7.83 (d, *J* = 8.1 Hz, 2H), 7.52(t, *J* = 7.6 Hz, 2H), 7.48-7.44 (m, 4H), 7.26 (t, *J* = 7.3 Hz, 1H), 6.87 (d, *J* = 8.3 Hz, 2H), 5.74 (s, 6H), 4.01 (s,6H).

^{13}C NMR (126 MHz, DMSO-d_6) δ 146.1, 143.7, 139.7, 134.2, 131.3, 125.2, 124.4, 120.6, 114.7, 112.7, 85.3, 31.9.

HR-MS (ESI positive): m/z calcd. for $C_{34}H_{37}ClN_5Ru$ $[M]^+$: 652.22 ; found: 652.1783.

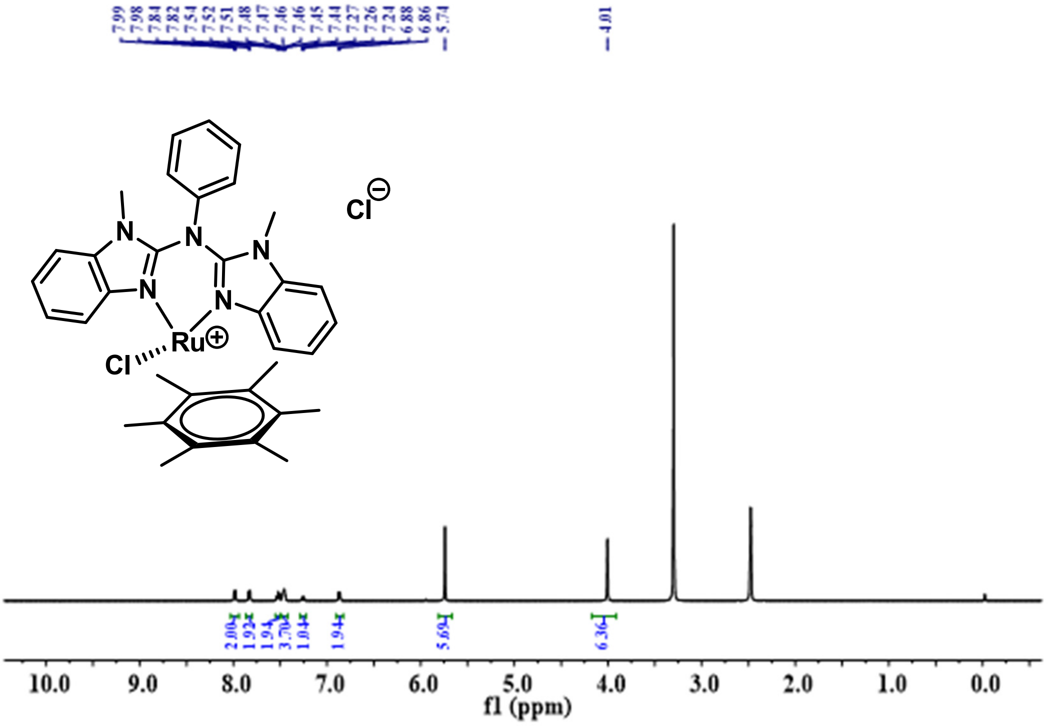

Figure S33 ^{13}C-NMR spectrum of **[Ru-9]**

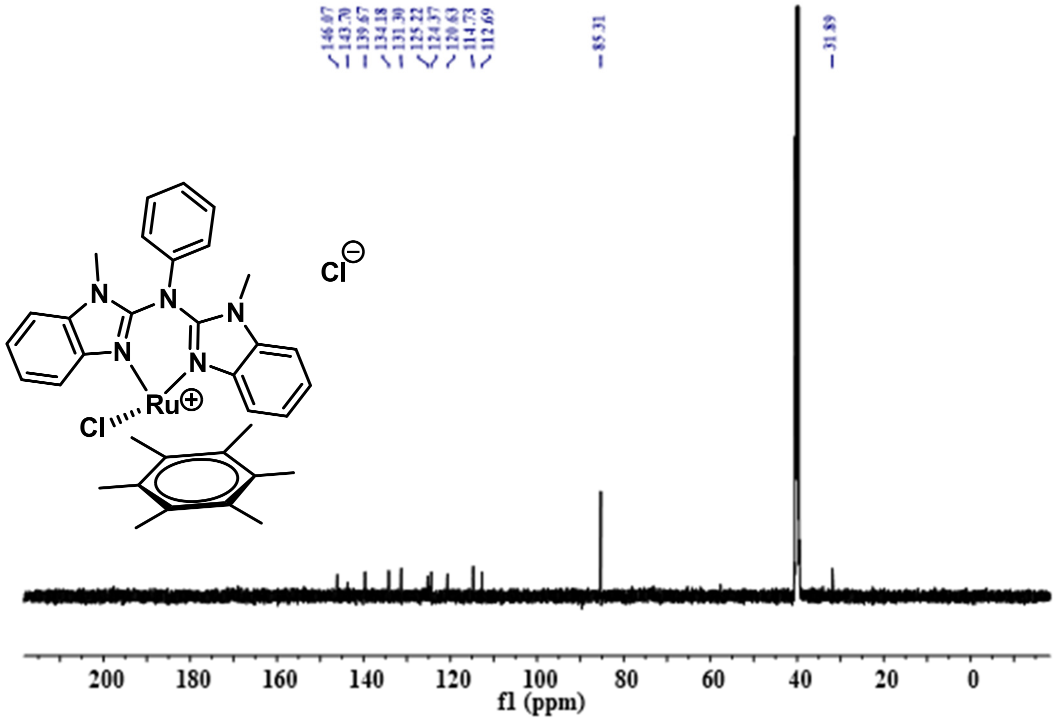

Figure S34 ^{13}C-NMR spectrum of **[Ru-9]**

3 Purified products ^{1}H and ^{13}C NMR spectra

3.1 1a

^{1}H NMR (500 MHz, $CDCl_3$) δ 12.38 (s, 1H), 8.17 (d, *J* = 7.8 Hz, 2H), 7.65 (t, *J* = 7.2 Hz, 1H), 7.52 (t, *J* = 7.5 Hz, 2H).

^{13}C NMR (126 MHz, $CDCl_3$) δ 172.4, 133.8, 130.2, 129.4, 128.5.

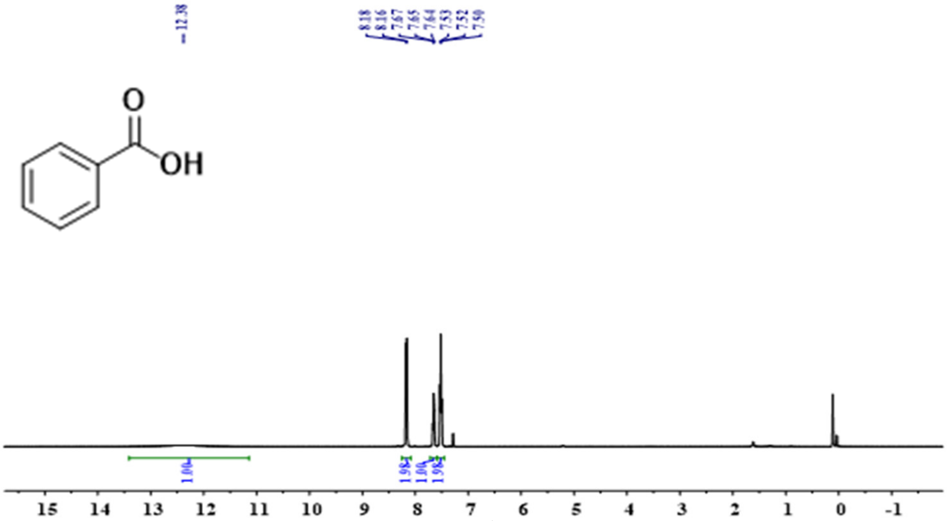

Figure S35 ^{1}H-NMR spectrum of **1a**

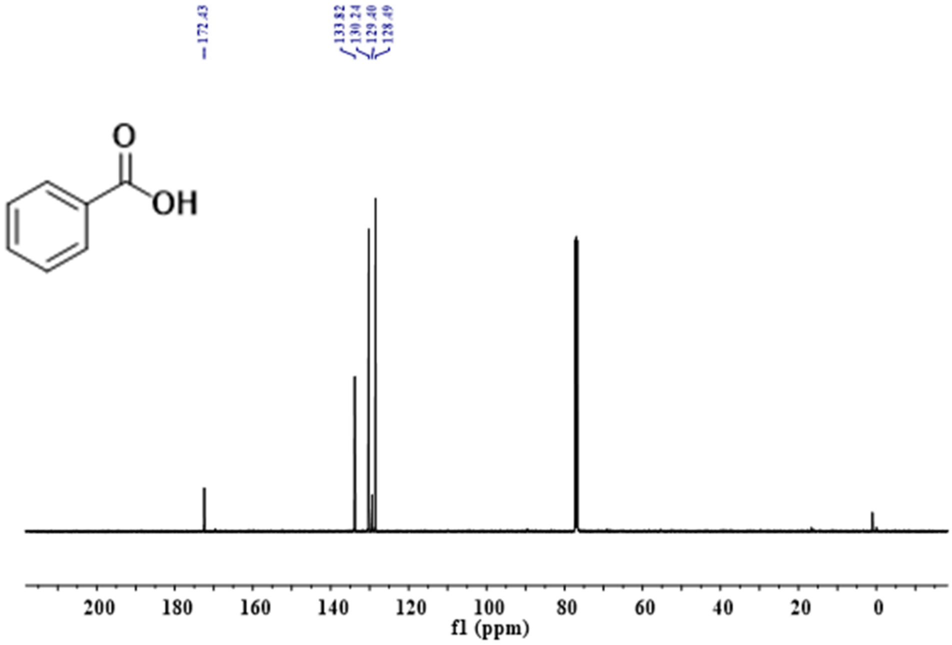

Figure S36 ^{13}C-NMR spectrum of **1a**

3.2 1b

^{1}H NMR (500 MHz, DMSO-d$_6$) δ 12.77 (s, 1H), 7.84 (d, *J* = 8.0 Hz, 2H), 7.31 (d, *J* = 7.9 Hz, 2H), 2.38 (s, 3H).

^{13}C NMR (126 MHz, DMSO-d$_6$) δ 167.8, 143.5, 129.8, 129.6, 128.5, 21.6.

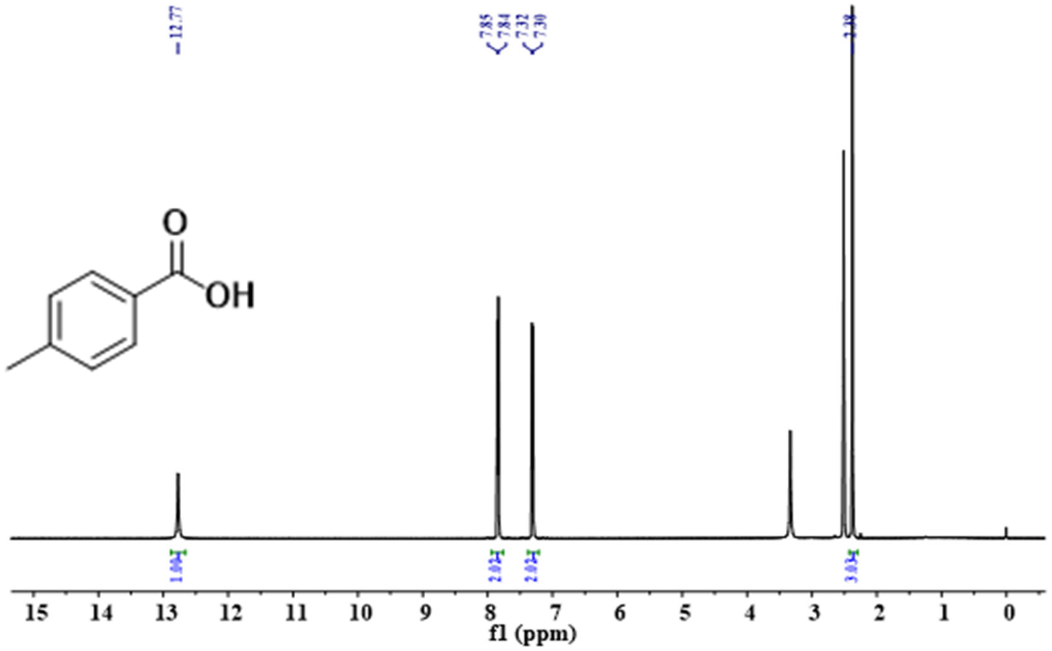

Figure S37 ^{1}H-NMR spectrum of **1b**

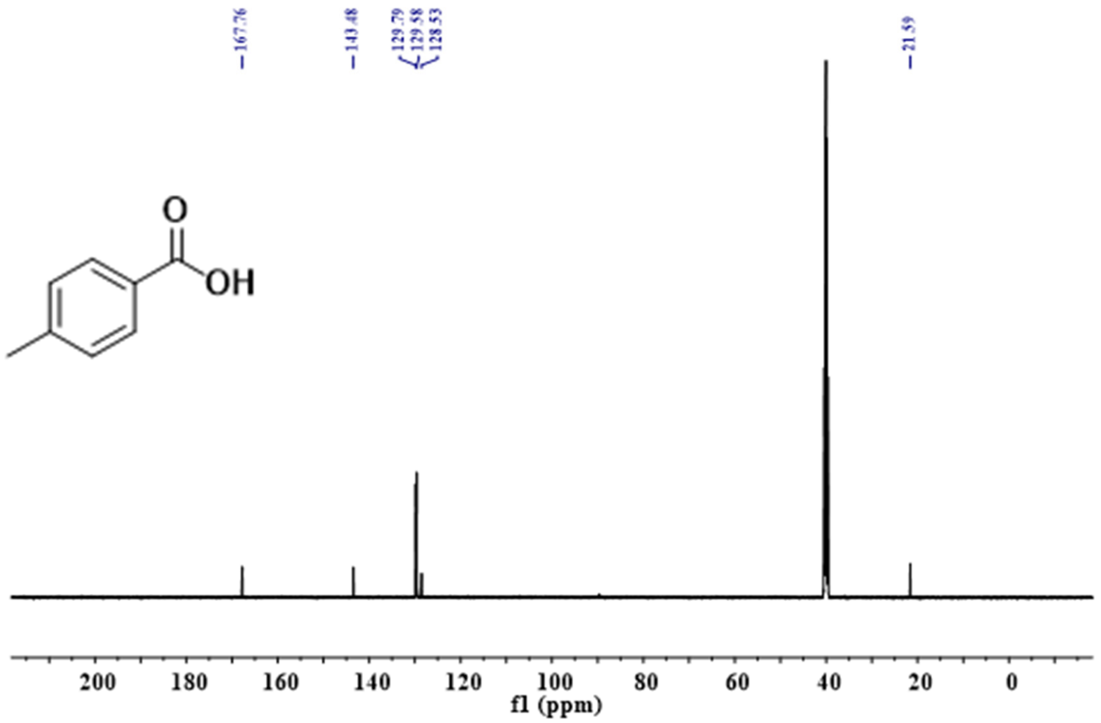

Figure S38 ^{13}C-NMR spectrum of **1b**

3.3 1c

^{1}H NMR (500 MHz, DMSO-d$_6$) δ 12.84 (s, 1H), 7.89 (s, 2H), 7.39 (s, 1H), 2.97 (s, 1H), 1.23 (s, 4H).

^{13}C NMR (126 MHz, DMSO-d$_6$) δ 167.7 (s), 154.0 (s), 129.9 (s), 126.9 (s), 126.0 (s), 33.9 (s), 24.0 (s).

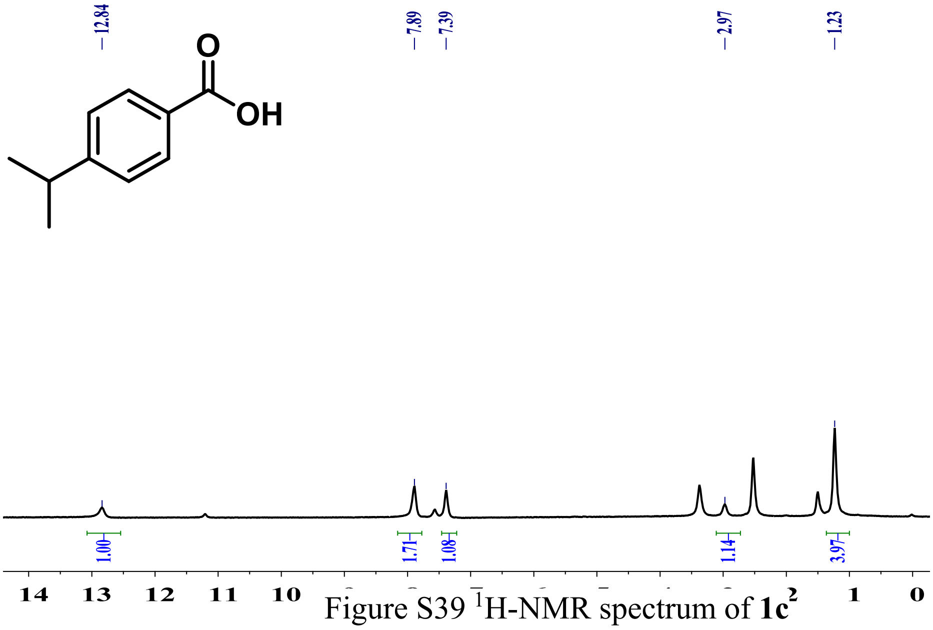

Figure S39 ^{1}H-NMR spectrum of **1c**

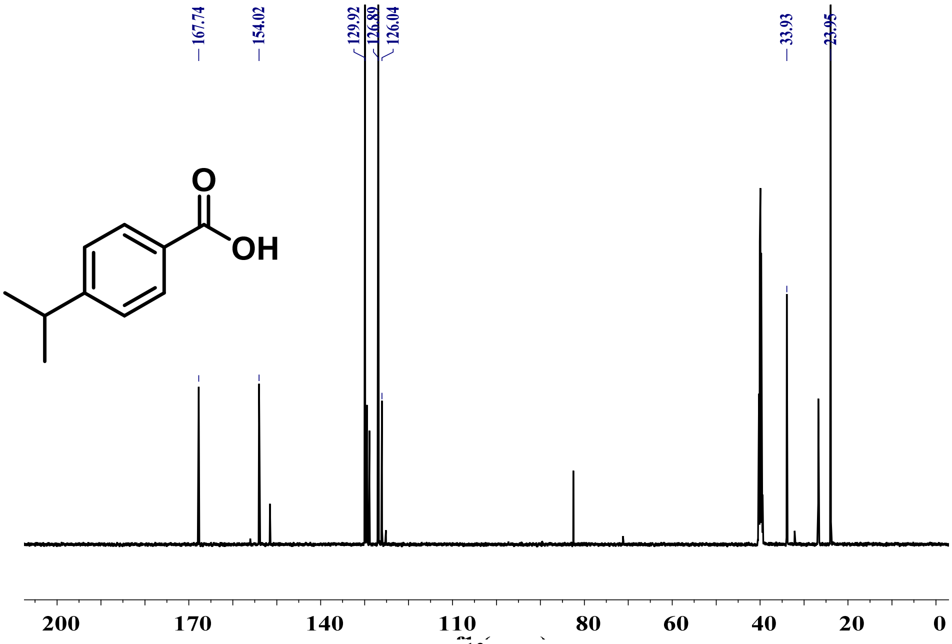

Figure S40 ^{13}C-NMR spectrum of **1c**

3.4 1d

^{1}H NMR (500 MHz, DMSO-d$_6$) δ 13.19 (s, 1H), 7.95 (d, *J* = 8.3 Hz, 2H), 7.58 (d, *J* = 8.3 Hz, 2H).

^{13}C NMR (126 MHz, DMSO-d$_6$) δ 166.9 (s), 138.2 (s), 131.6 (s), 130.1 (s), 129.2 (s).

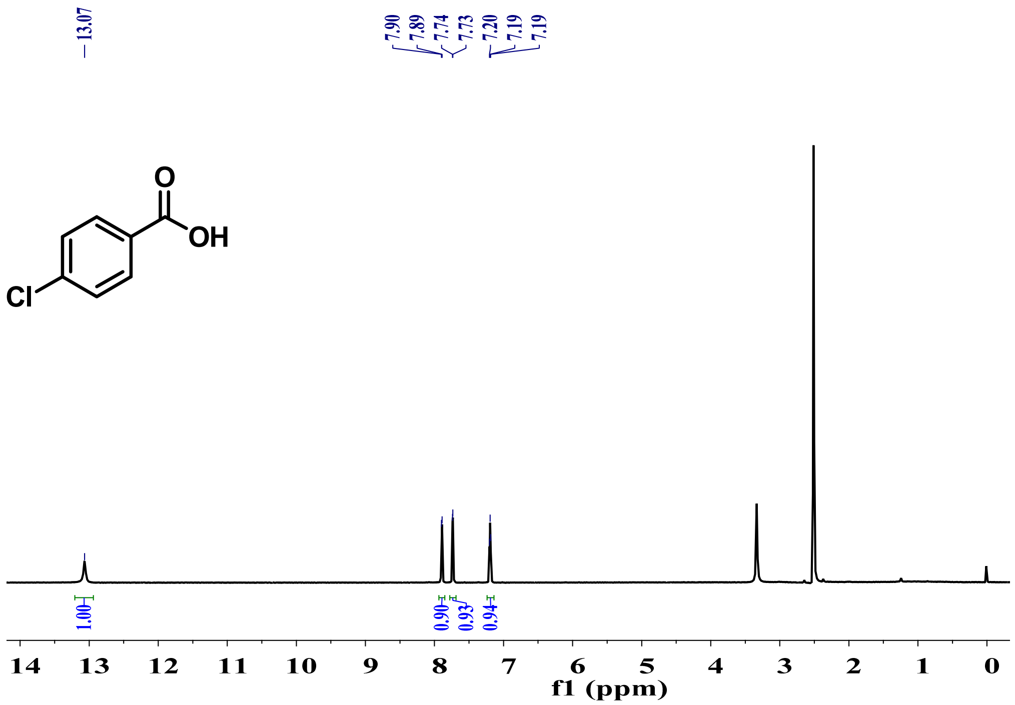

Figure S41 ^{1}H-NMR spectrum of **1d**

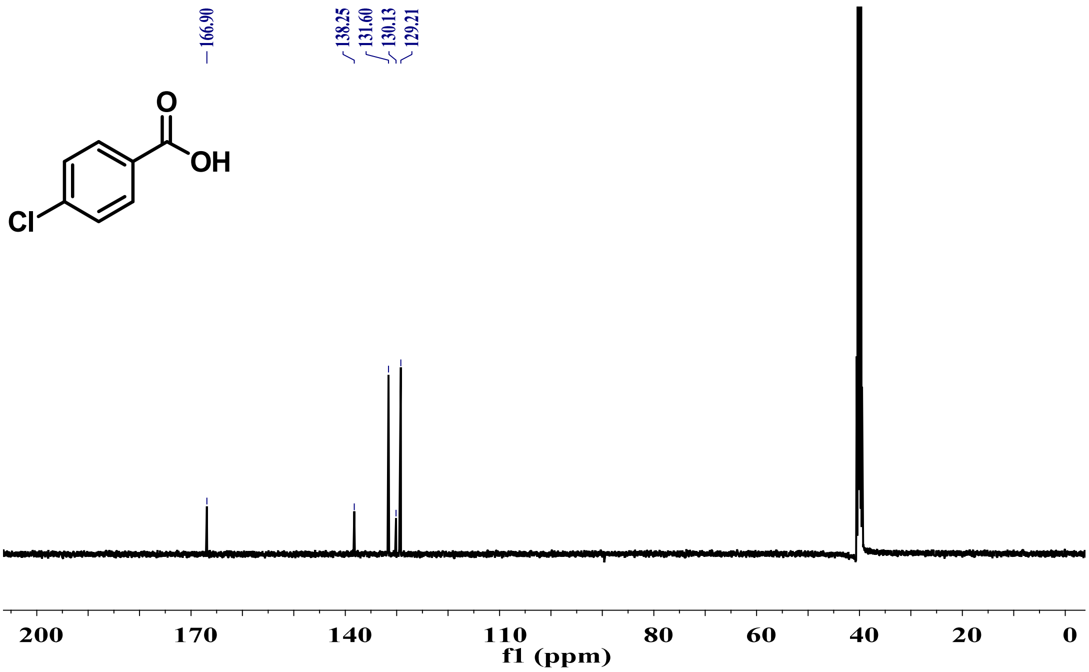

Figure S42 ^{13}C-NMR spectrum of **1d**

3.5 1e

^{1}H NMR (500 MHz, DMSO-d6) δ 13.19 (s, 1H), 7.87 (d, *J* = 8.3 Hz, 2H), 7.71 (d, *J* = 8.3 Hz, 2H).

^{13}C NMR (126 MHz, DMSO-d6) δ 167.1 (s), 132.2 (s), 131.7 (s), 130.5 (s), 127.3 (s).

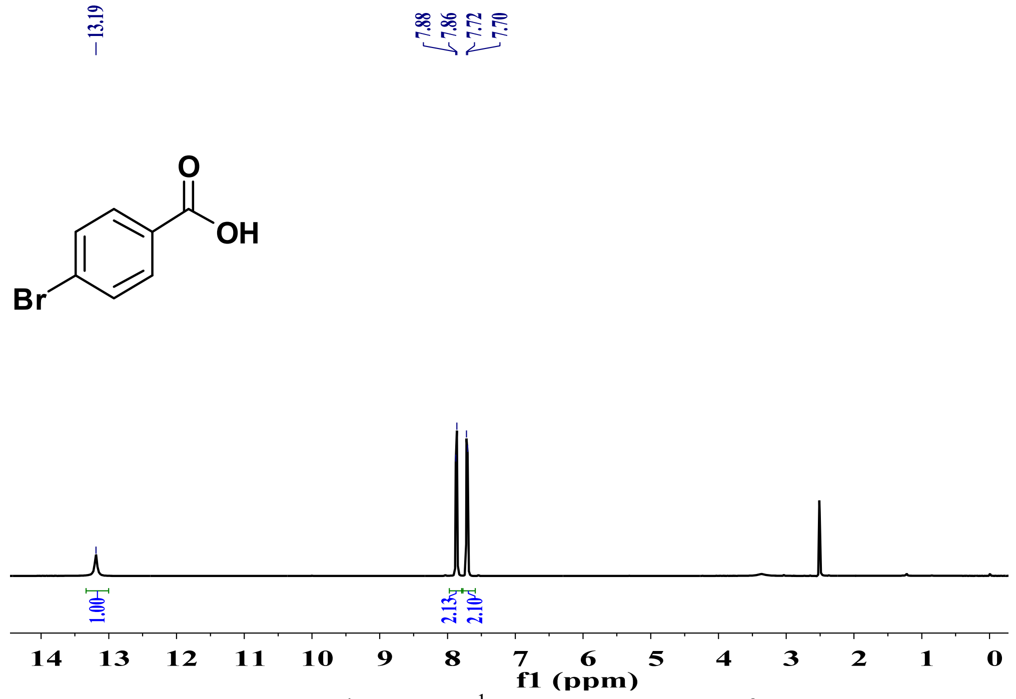

Figure S43 ^{1}H-NMR spectrum of **1e**

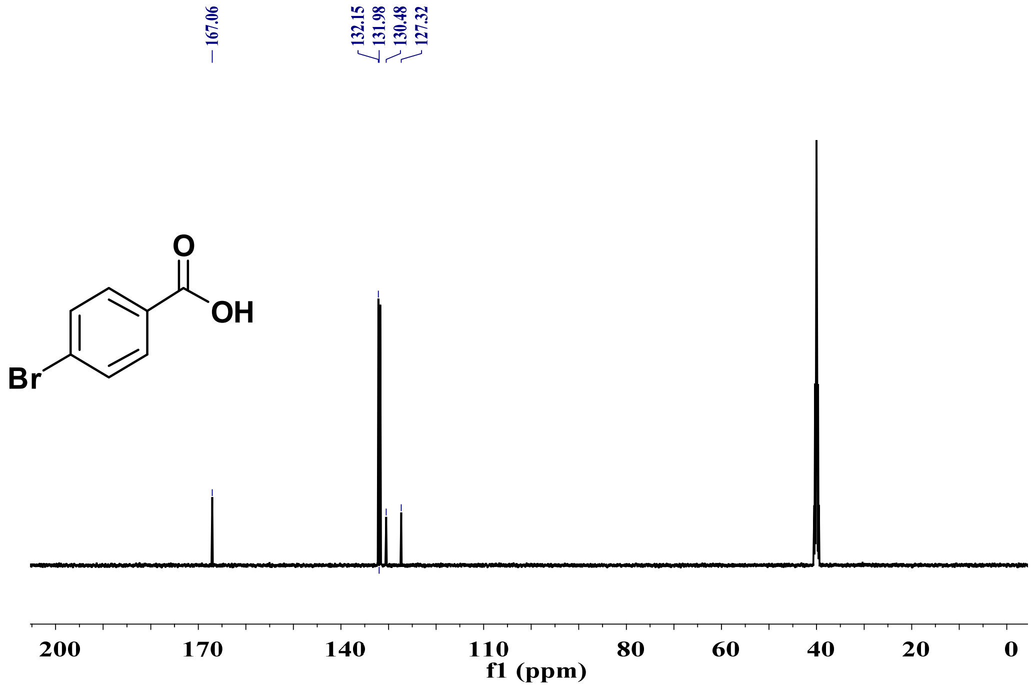

Figure S44 ^{13}C-NMR spectrum of **1e**

3.6 1f

^{1}H NMR (500 MHz, DMSO-d6) δ 12.42 (s, 1H), 10.22 (s, 1H), 7.84 (t, *J* = 39.5 Hz, 2H), 6.83 (d, *J* = 8.5 Hz, 2H).

^{13}C NMR (126 MHz, DMSO-d6) δ 167.6 (s), 162.1 (s), 132.0 (s), 121.8 (s), 115.6 (s).

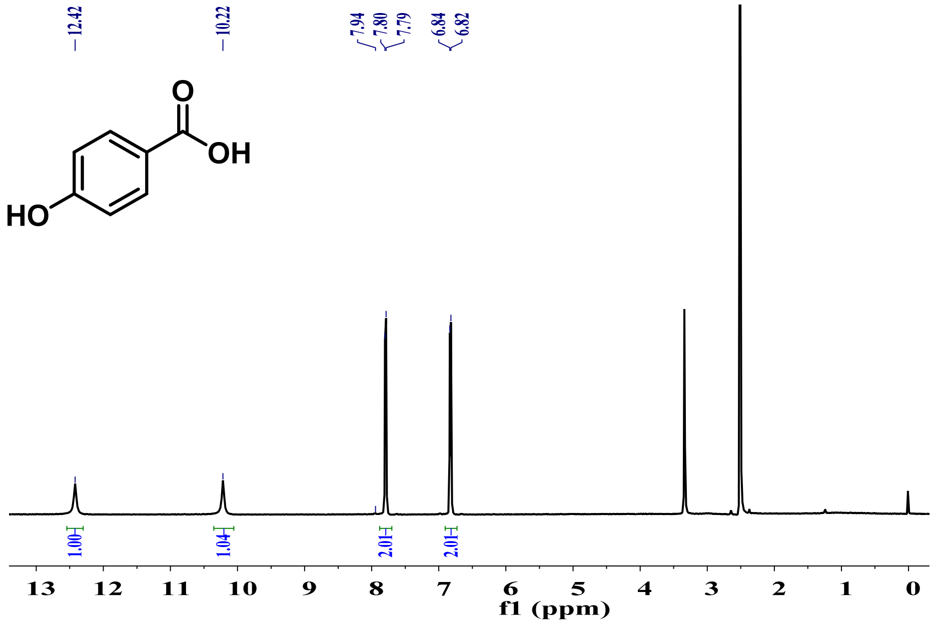

Figure S45 ^{1}H-NMR spectrum of **1e**

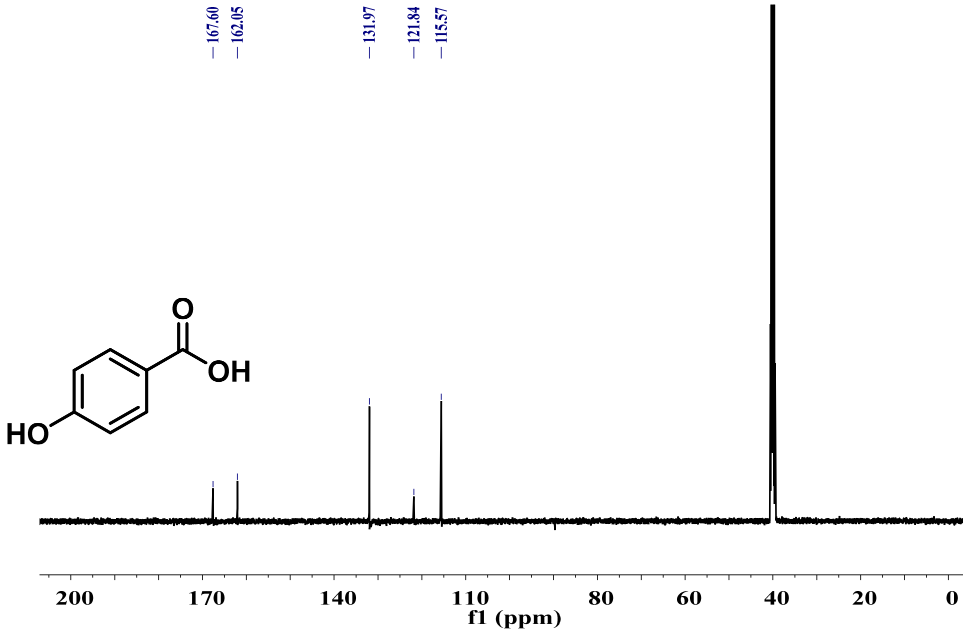

Figure S46 ^{13}C-NMR spectrum of **1f**

3.7 1g

^{1}H NMR (500 MHz, $CDCl_3$) δ 12.09 (s, 1H), 8.10 (d, J = 7.9 Hz, 1H), 7.49 (t, J = 7.4 Hz, 1H), 7.33-7.29 (m,2H), 2.70 (s, 3H).
^{13}C NMR (126 MHz, $CDCl_3$) δ 172.9, 141.3, 132.9, 131.9, 131.6, 128.3, 125.9, 22.1.

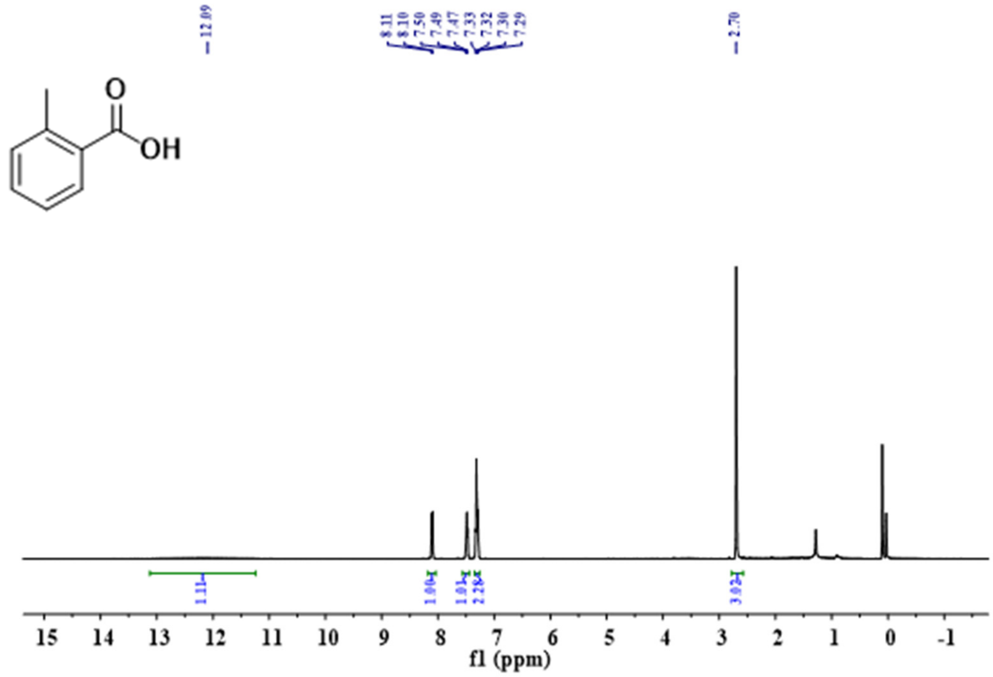

Figure S47 ^{1}H-NMR spectrum of **1g**

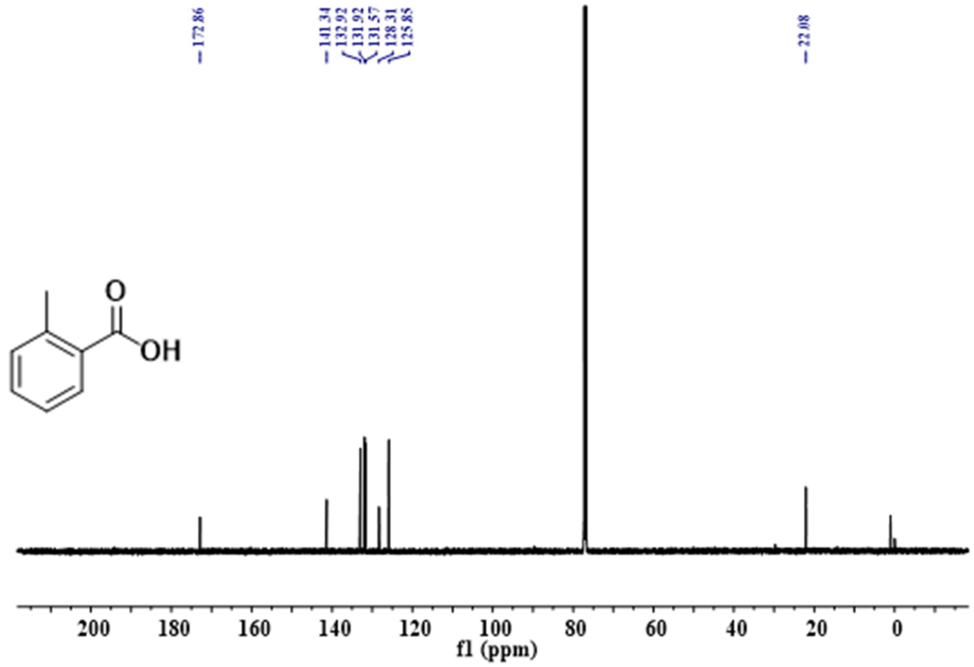

Figure S48 ^{13}C-NMR spectrum of **1g**

3.8 1h

^{1}H NMR (500 MHz, DMSO-d$_6$) δ 12.87 (s, 1H), 7.89 – 7.68 (m, 2H), 7.57 – 7.20 (m, 2H), 2.37 (s, 3H).

^{13}C NMR (126 MHz, DMSO-d$_6$) δ 167.9 (s), 138.3 (s), 133.9 (s), 131.2 (s), 130.2 (s), 128.9 (s), 126.9 (s), 21.3 (s).

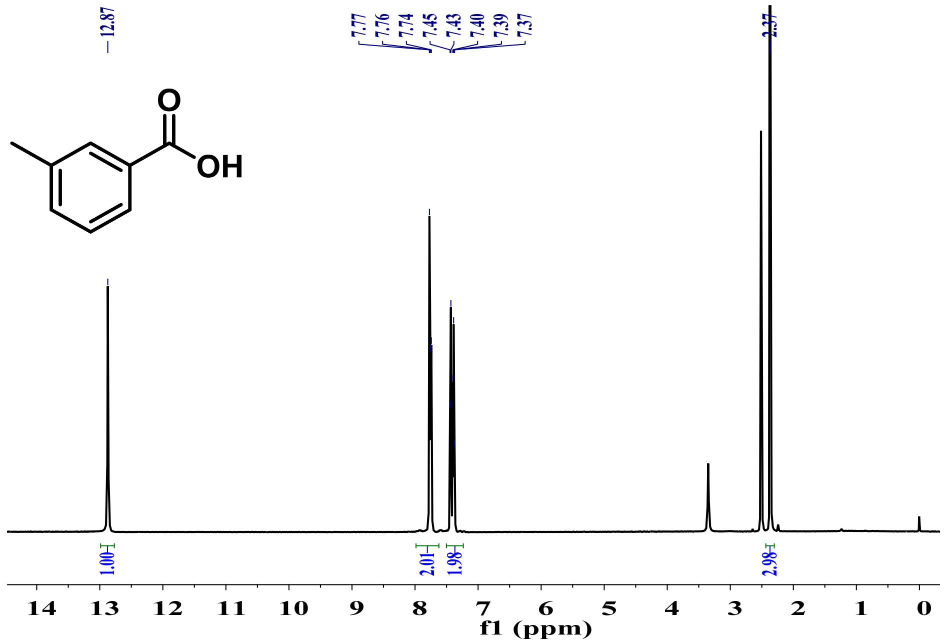

Figure S49 ^{1}H-NMR spectrum of **1h**

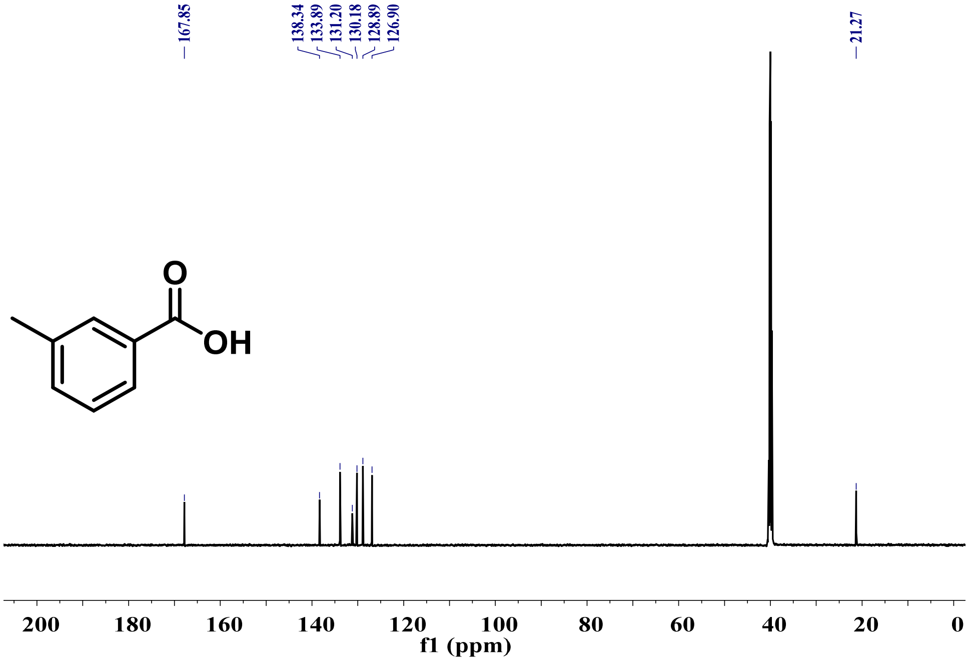

Figure S50 ^{13}C-NMR spectrum of **1h**

3.9 1i

^{1}H NMR (500 MHz, DMSO-d$_6$) δ 13.09 (s, 1H), 8.62 (s, 1H), 8.13 (d, *J* = 8.1 Hz, 1H), 8.06 – 7.94 (m, 3H), 7.67 (t, *J* = 7.5 Hz, 1H), 7.62 (t, *J* = 7.4 Hz, 1H).

^{13}C NMR (126 MHz, DMSO-d$_6$) δ 167.9, 135.4, 132.6, 131.0, 129.7, 128.8, 128.6, 128.6, 128.1, 127.3, 125.6.

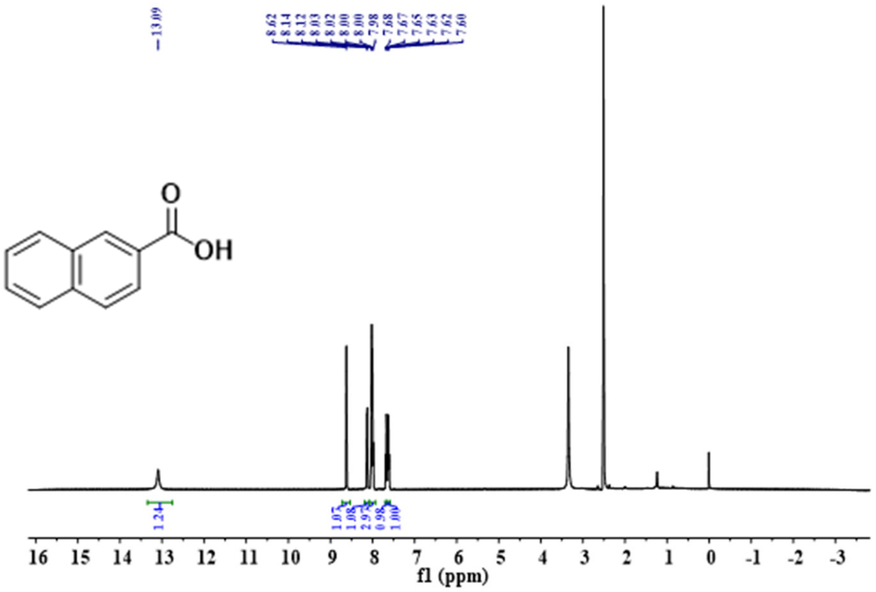

Figure S51 ^{1}H-NMR spectrum of **1i**

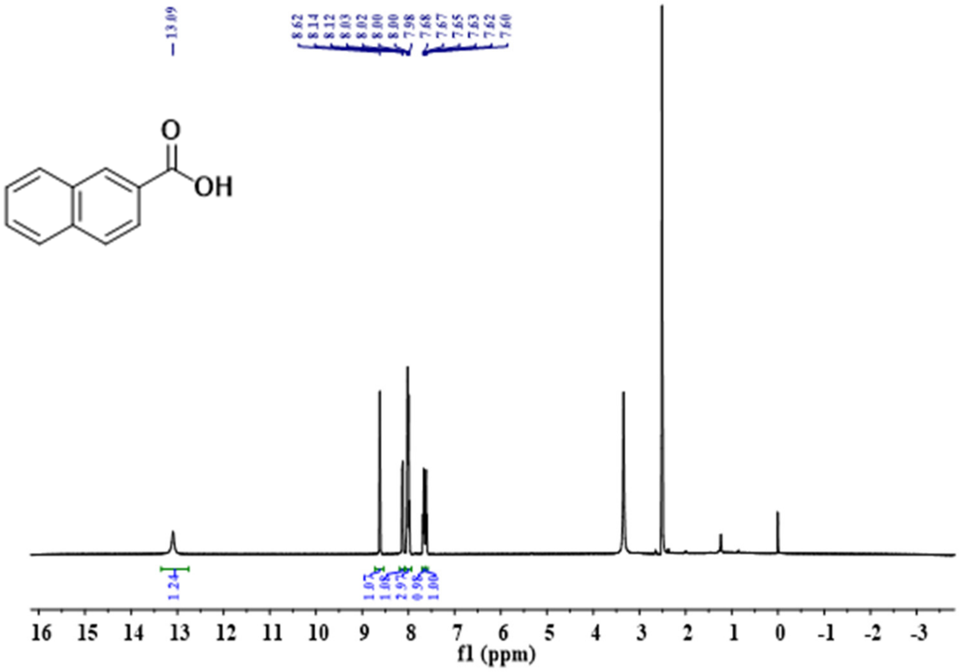

Figure S52 ^{13}C-NMR spectrum of **1i**

3.10 1j

^{1}H NMR (500 MHz, DMSO-d$_6$) δ 13.07 (s, 1H), 7.90 (d, *J* = 4.8 Hz, 1H), 7.74 (d, *J* = 3.3 Hz, 1H), 7.20 (t, *J* = 4.2 Hz, 1H).

^{13}C NMR (126 MHz, DMSO-d$_6$) δ 163.3 (s), 135.1 (s), 133.6 (d, *J* = 8.1 Hz), 128.6 (s).

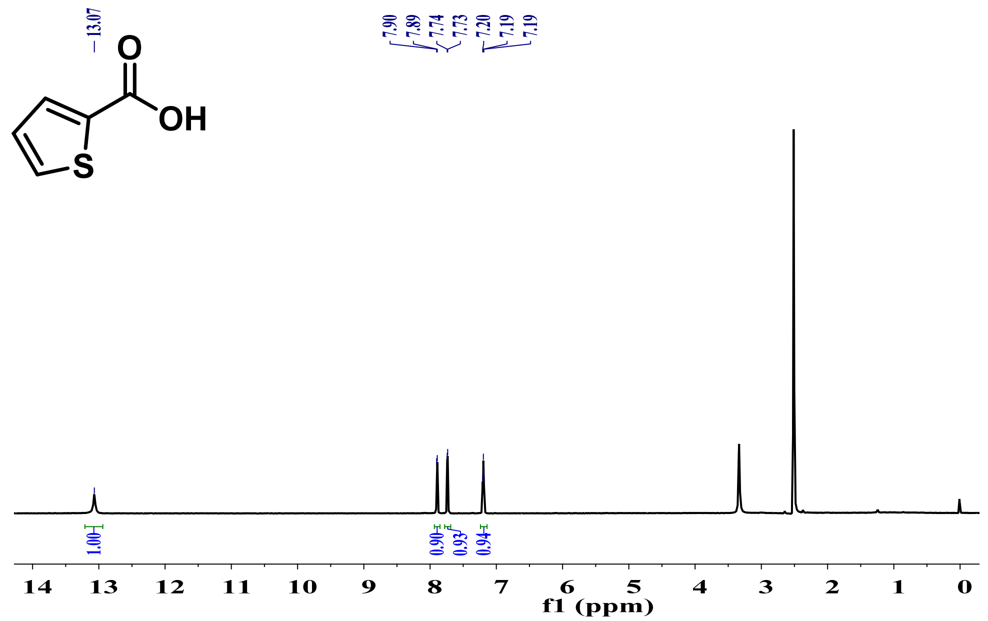

Figure S53 ^{1}H-NMR spectrum of **1j**

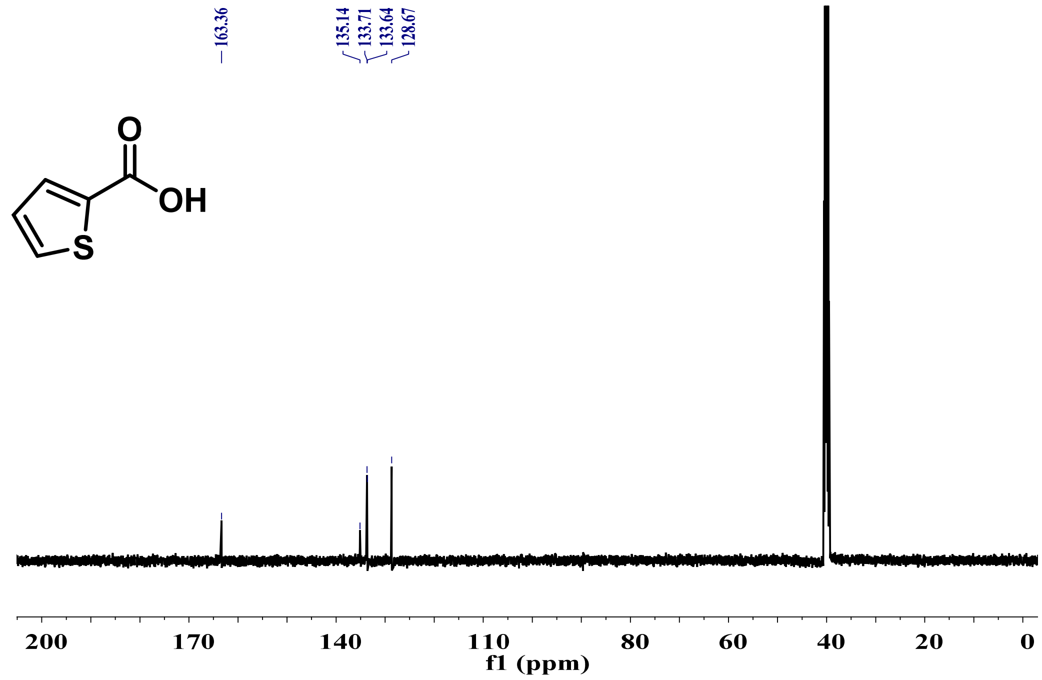

Figure S54 ^{13}C-NMR spectrum of **1j**

3.11 1k

^{1}H NMR (500 MHz, $CDCl_3$) δ 12.17 (s, 1H), 2.42 – 2.16 (m, 1H), 1.91 (d, J = 13.1 Hz, 2H), 1.73 (dd, J = 9.4, 3.3 Hz, 2H), 1.62 (d, J = 10.1 Hz, 1H), 1.50 – 1.37 (m, 2H), 1.32 – 1.14 (m, 3H).

^{13}C NMR (126 MHz, $CDCl_3$) δ 182.9, 42.9, 28.7, 25.7, 25.3.

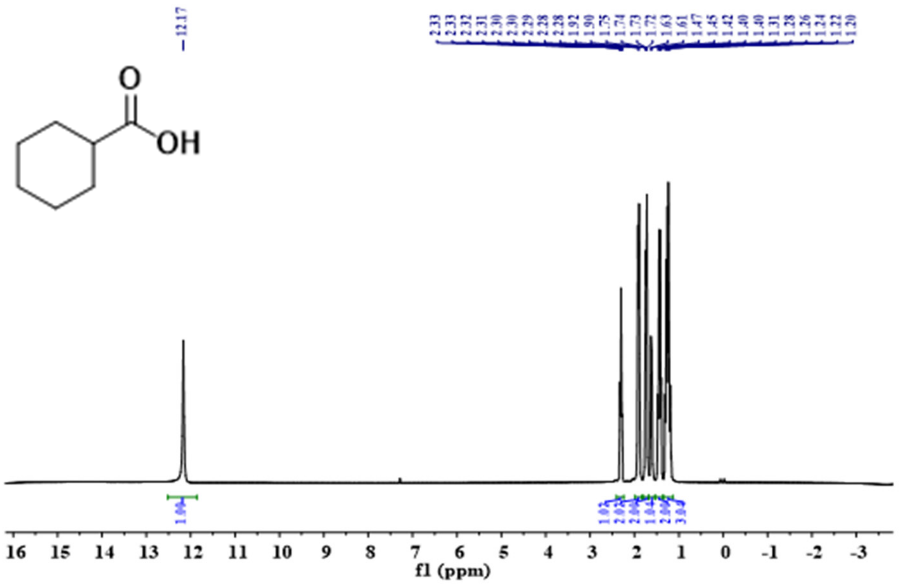

Figure S55 ^{1}H-NMR spectrum of **1k**

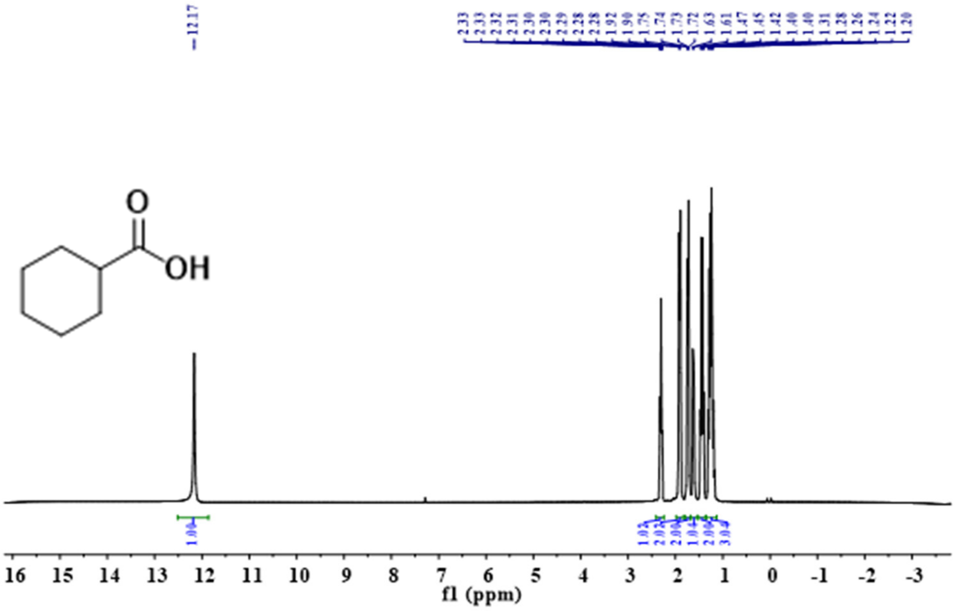

Figure S56 ^{13}C-NMR spectrum of **1k**

3.12 1l

^{1}H NMR (500 MHz, $CDCl_3$) δ 11.66 (s, 1H), 2.37 (t, J = 7.5 Hz, 2H), 1.73 – 1.53 (m, 2H), 1.35 (d, J = 7.0 Hz, 4H), 0.92 (s, 3H).

^{13}C NMR (126 MHz, $CDCl_3$) δ 180.6, 180.6, 34.1, 31.2, 24.3, 22.3, 13.8.

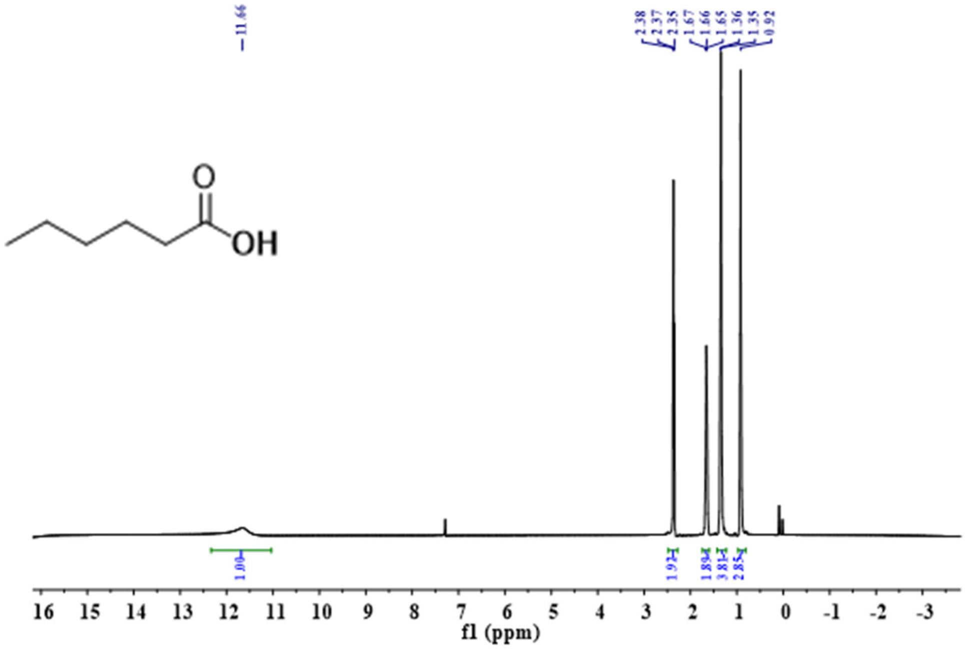

Figure S57 ^{1}H-NMR spectrum of **1l**

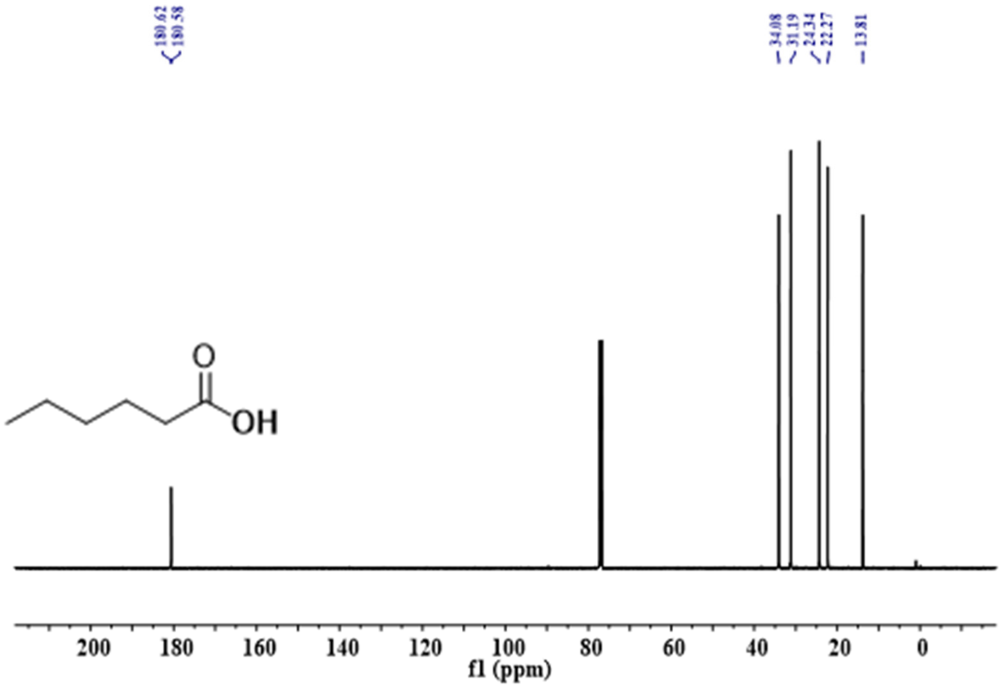

Figure S58 ^{13}C-NMR spectrum of **1l**

3.13 1m

^1H NMR (500 MHz, DMSO-d6) δ 11.94 (s, 1H), 2.25 – 2.00 (m, 2H), 1.50 (s, 2H), 1.26 (s, 9H), 0.96 – 0.75 (m, 3H).

^{13}C NMR (126 MHz, DMSO-d6) δ 174.8, 34.1, 31.6, 29.0, 28.9, 25.0, 22.5, 14.3.

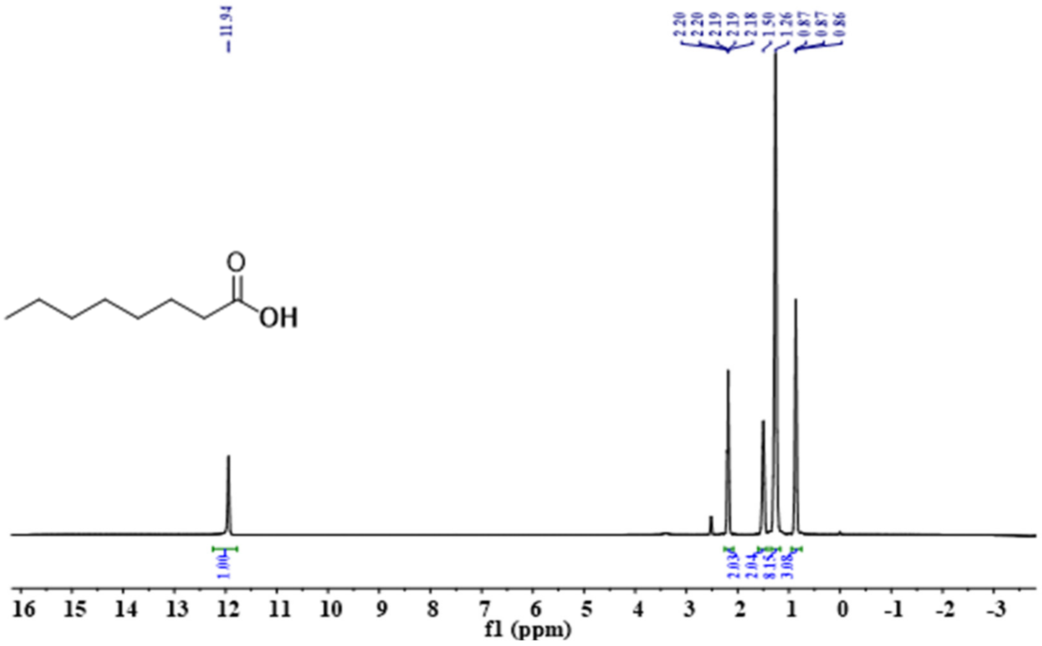

Figure S59 ^1H-NMR spectrum of **1m**

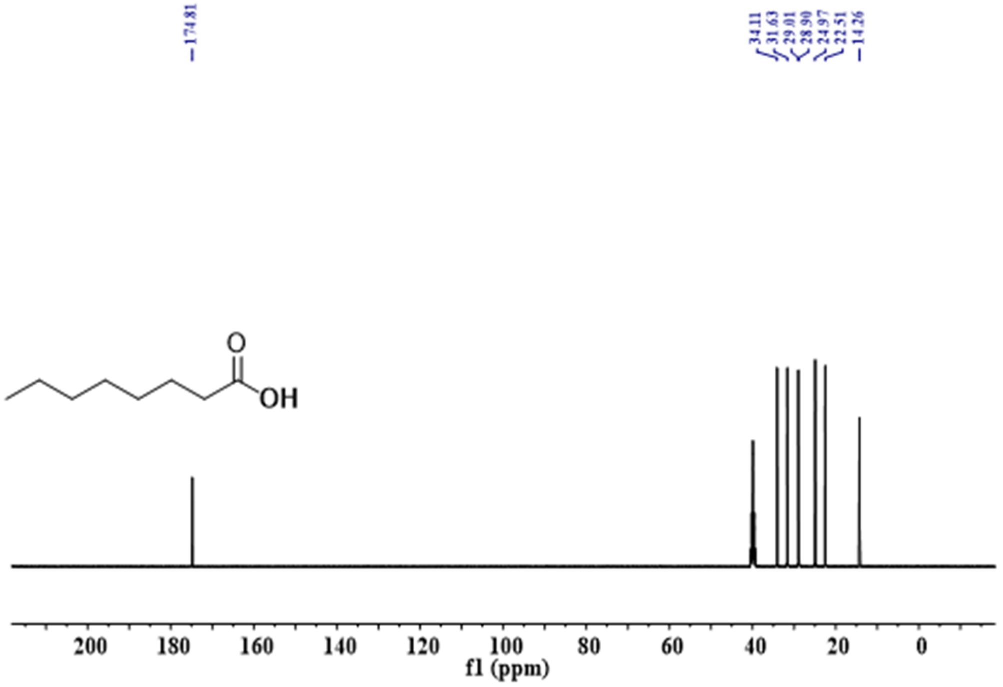

Figure S60 ^{13}C-NMR spectrum of **1m**

3.14 1n

^{1}H NMR (500 MHz, DMSO-d$_6$) δ 13.05 (s, 1H), 7.92 (d, *J* = 0.6 Hz, 1H), 7.22 (d, *J* = 2.9 Hz, 1H), 6.66 (dd, *J* = 3.3, 1.6 Hz, 1H).

^{13}C NMR (126 MHz, DMSO-d$_6$) δ 159.74 (s), 147.46 (s), 145.39 (s), 118.13 (s), 112.52 (s).

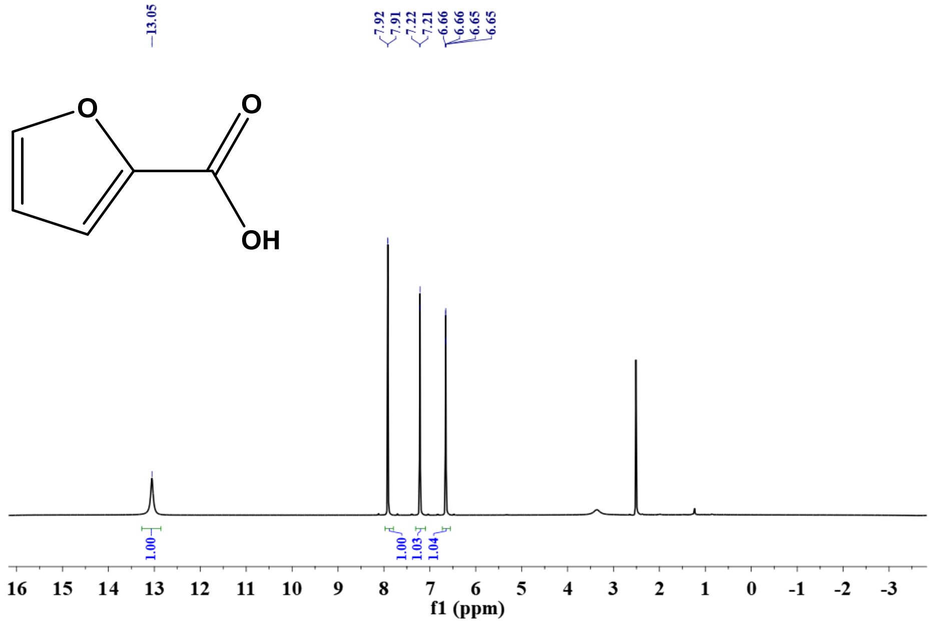

Figure S61 ^{1}H-NMR spectrum of **1n**

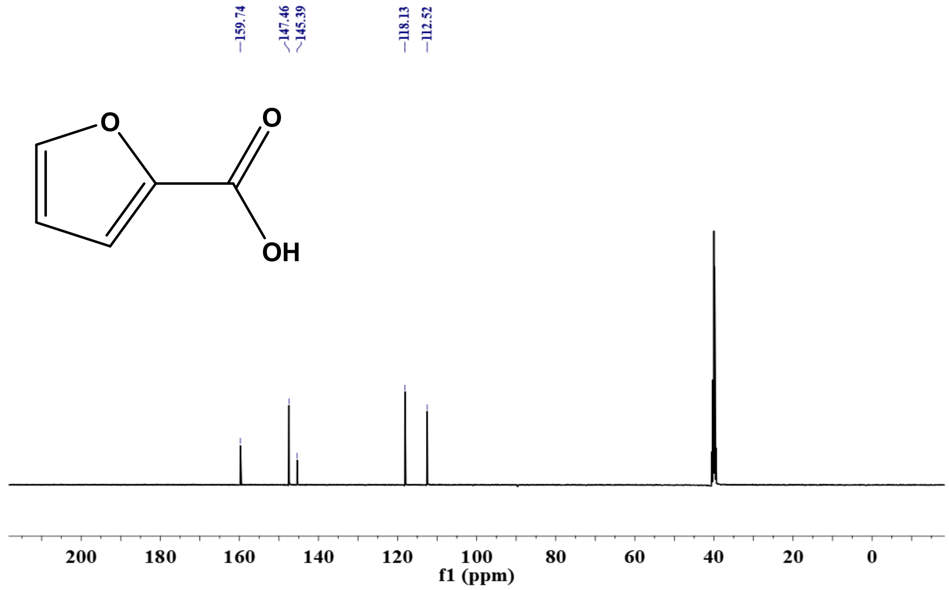

Figure S62 ^{13}C-NMR spectrum of **1n**

3.15 1o

^{1}H NMR (500 MHz, DMSO-d$_6$) δ 13.42 (s, 1H), 9.08 (d, *J* = 1.2 Hz, 1H), 8.84 – 8.75 (m, 1H), 8.38 – 8.16 (m, 1H), 7.55 (dd, *J* = 7.6, 4.8 Hz, 1H).

^{13}C NMR (126 MHz, DMSO-d$_6$) δ 166.7, 153.7, 150.7, 137.4, 127.0, 124.2.

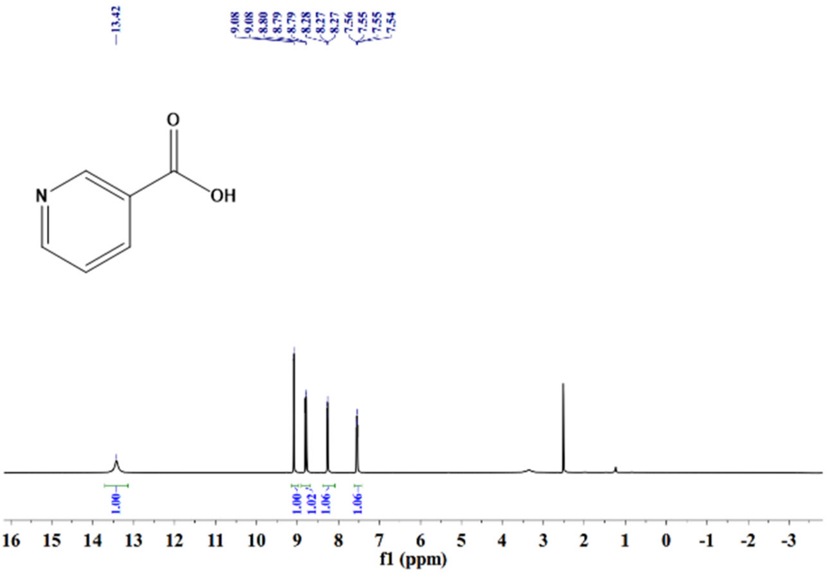

Figure S63 ^{1}H-NMR spectrum of **1o**

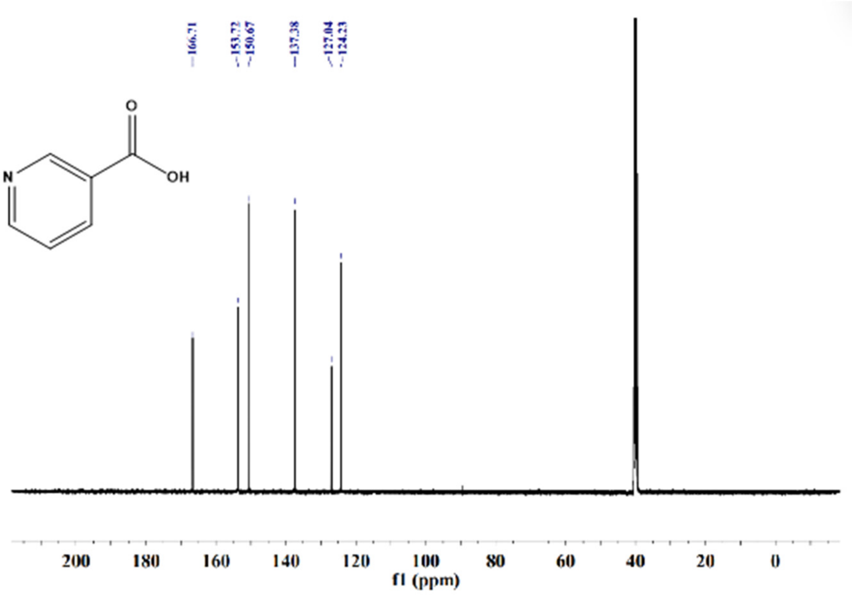

Figure S64 ^{13}C-NMR spectrum of **1o**

3.16 **1p**

^{1}H NMR (500 MHz, DMSO-d$_6$) δ 13.11 (s, 1H), 8.71 (d, *J* = 3.9 Hz, 1H), 8.06 (d, *J* = 7.7 Hz, 1H), 7.99 (td, *J* = 7.7, 1.6 Hz, 1H), 7.63 (dd, *J* = 6.3, 4.8 Hz, 1H).

^{13}C NMR (126 MHz, DMSO-d$_6$) δ 166.6, 149.9, 148.8, 138.0, 127.5, 125.1.

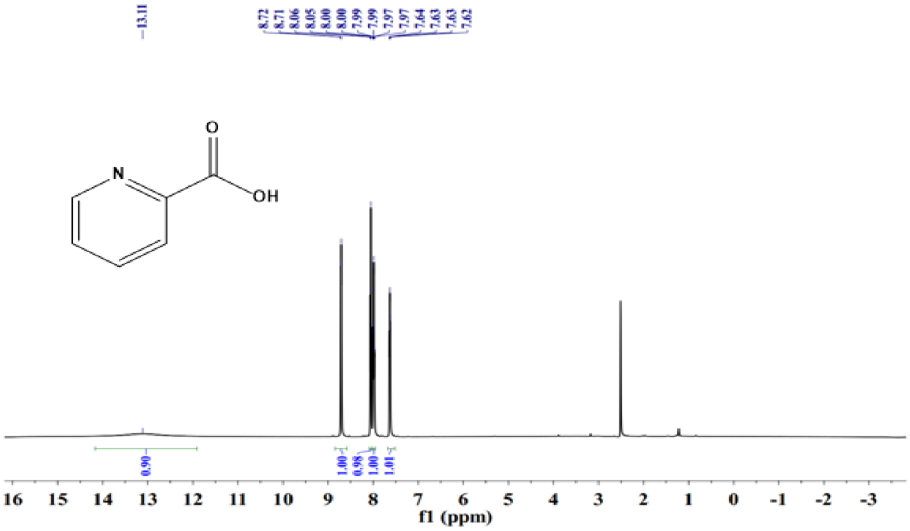

Figure S65 ^{1}H-NMR spectrum of **1p**

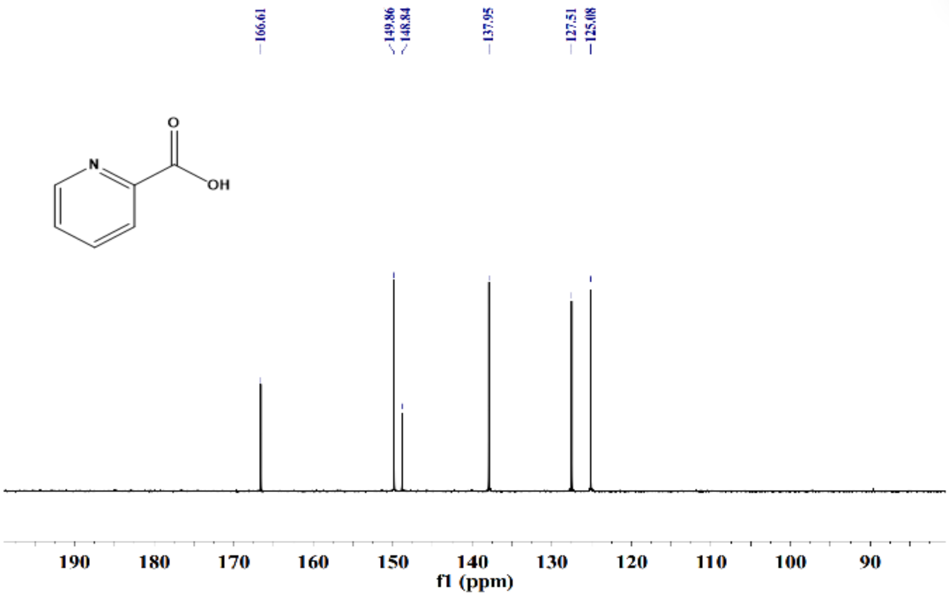

Figure S66 ^{13}C-NMR spectrum of **1p**

Table S1. Comparison table of different catalysts for alcohol dehydrogenation.

Entry	Catalyst	[Cat.] (mol%)	Base (equiv.)	Time (h)	Tem. (℃)	Solvent (mL)	Yield (%)	Reference
1	1a	0.002	KOH (2)	12	120	Toluene (1.5)	96	[1]
2	2	0.001	KOH (1.1)	12	120	Toluene (6)	99	[2]
3	1	0.01	KOH (1.2)	6	120	Toluene (5)	79	[3]
4	3	0.01	KOH (1.1)	20	120	degassed water (2)	85	[4]
5	1	0.01	NaOH (2)	14	100	water, dioxane (1)	90	[5]
6	Ru-1	2	KOH (1.1)	6	110	toluene (2)	92	[6]
7	Ru-2	0.025	KOH (1.2)	6	140	m-xylene (1.5)	94	[7]
8	[Ru-2]	0.003	KOH (1)	16	120	Toluene (0.4)	91.2	**This work**

[1] Yin, S.; Zheng, Q.; Chen, J.; Tu, T. Acceptorless Dehydrogenation of Primary Alcohols to Carboxylic Acids by Self-Supported NHC-Ru Single-Site Catalysts. *J Catal* **2022**, *408*, 165–172, doi:10.1016/j.jcat.2022.02.018.

[2] Chen, Z.W.; Ma, F.; Liu, Y.; Mo, X.F.; Chen, G.; Peng, X.; Yi, X.Y. Geometrical Isomerization and Acceptorless Dehydrogenative Alcohol Oxidation Based on Pyrrole-Based Ru(Ⅱ) Complexes. *Inorg Chim Acta* **2022**, *541*, doi:10.1016/j.ica.2022.121034.

[3] Wang, W.Q.; Chen, C.; [illegible] Sang, W.; Chen, C.; Verpoort, F. Highly Efficient N-Heterocyclic Carbene/Ruthenium Catalytic Systems for the Acceptorless Dehydrogenation of Alcohols to Carboxylic Acids: Effects of Ancillary and Additional Ligands. *Catalysts* **2020**, *10*, doi:10.3390/catal10010010.

[4] Choi, J.H.; Heim, L.E.; Ahrens, M.; Prechtl, M.H.G. Selective Conversion of Alcohols in Water to Carboxylic Acids by in Situ Generated Ruthenium Trans Dihydrido Carbonyl PNP Complexes. *Dalton Transactions* **2014**, *43*, 17248–17254, doi:10.1039/c4dt01634c.

[5] Singh, A.; Singh, S.K.; Saini, A.K.; Mobin, S.M.; Mathur, P. Facile Oxidation of Alcohols to Carboxylic Acids in Basic Water Medium by Employing Ruthenium Picolinate Cluster as an Efficient Catalyst. *Appl Organomet Chem* **2018**, *32*, doi:10.1002/aoc.4574.

[6] Awasthi, M.K.; Singh, S.K. Ruthenium Catalyzed Dehydrogenation of Alcohols and Mechanistic Study. *Inorg Chem* **2019**, *58*, 14912–14923, doi:10.1021/acs.inorgchem.9b02691.

[7] Wang, Z.Q.; Tang, X.S.; Yang, Z.Q.; Yu, B.Y.; Wang, H.J.; Sang, W.; Yuan, Y.; Chen, C.; Verpoort, F. Highly Active Bidentate N-Heterocyclic Carbene/Ruthenium Complexes Performing Dehydrogenative Coupling of Alcohols and Hydroxides in Open Air. *Chemical Communications* **2019**, *55*, 8591–8594, doi:10.1039/c9cc03519b.
